# A Novel Synthetic Dual Agonistic Liposomal TLR4/7 Adjuvant Promotes Broad Immune Responses in an Influenza Vaccine With Minimal Reactogenicity

**DOI:** 10.3389/fimmu.2020.01207

**Published:** 2020-06-19

**Authors:** Fumi Sato-Kaneko, Shiyin Yao, Fitzgerald S. Lao, Jonathan Shpigelman, Karen Messer, Minya Pu, Nikunj M. Shukla, Howard B. Cottam, Michael Chan, Paul J. Chu, David Burkhart, Roman Schoener, Takaji Matsutani, Dennis A. Carson, Maripat Corr, Tomoko Hayashi

**Affiliations:** ^1^Moores Cancer Center, University of California, San Diego, La Jolla, CA, United States; ^2^Division of Biostatistics, University of California, San Diego, La Jolla, CA, United States; ^3^Inimmune Corp., Missoula, MT, United States; ^4^Repertoire Genesis Inc., Saito-Asagai, Osaka, Japan; ^5^Department of Medicine, University of California, San Diego, La Jolla, CA, United States

**Keywords:** vaccine, combination adjuvant, synthetic TLR4 agonist, synthetic TLR7 agonists, small molecule, influenza virus infection

## Abstract

The limited efficacy of seasonal influenza vaccines is usually attributed to ongoing variation in the major antigenic targets for protective antibody responses including hemagglutinin (HA) and neuraminidase (NA). Hence, vaccine development has largely focused on broadening antigenic epitopes to generate cross-reactive protection. However, the vaccine adjuvant components which can accelerate, enhance and prolong antigenic immune responses, can also increase the breadth of these responses. We previously demonstrated that the combination of synthetic small-molecule Toll-like receptor 4 (TLR4) and TLR7 ligands is a potent adjuvant for recombinant influenza virus HA, inducing rapid, and sustained antibody responses that are protective against influenza viruses in homologous and heterologous murine challenge models. To further enhance adjuvant efficacy, we performed a structure-activity relationship study for the TLR4 ligand, *N*-cyclohexyl-2-((5-methyl-4-oxo-3-phenyl-4,5-dihydro-3H-pyrimido[5,4-*b*]indol-2-yl)thio)acetamide (C_25_H_26_N_4_O_2_S; **1Z105**), and identified the 8-(furan-2-yl) substituted pyrimido[5,4-*b*]indole analog (C_29_H_28_N_4_O_3_S; **2B182C**) as a derivative with higher potency in activating both human and mouse TLR4-NF-κB reporter cells and primary cells. In a prime-boost immunization model using inactivated influenza A virus [IIAV; A/California/04/2009 (H1N1)pdm09], **2B182C** used as adjuvant induced higher serum anti-HA and anti-NA IgG1 levels compared to **1Z105**, and also increased the anti-NA IgG2a responses. In combination with a TLR7 ligand, **1V270**, **2B182C** induced equivalent levels of anti-NA and anti-HA IgG1 to **1V270+1Z105**. However, the combination of **1V270+2B182C** induced 10-fold higher anti-HA and anti-NA IgG2a levels compared to **1V270+1Z105**. A stable liposomal formulation of **1V270+2B182C** was developed, which synergistically enhanced anti-HA and anti-NA IgG1 and IgG2a responses without demonstrable reactogenicity after intramuscular injection. Notably, vaccination with IIAV plus the liposomal formulation of **1V270+2B182C** protected mice against lethal homologous influenza virus (H1N1)pdm09 challenge and reduced lung viral titers and cytokine levels. The combination adjuvant induced a greater diversity in B cell clonotypes of immunoglobulin heavy chain (IGH) genes in the draining lymph nodes and antibodies against a broad spectrum of HA epitopes encompassing HA head and stalk domains and with cross-reactivity against different subtypes of HA and NA. This novel combination liposomal adjuvant contributes to a more broadly protective vaccine while demonstrating an attractive safety profile.

## Introduction

Global public health emergencies from zoonotic infections stress the imperative need for vaccines with rapid protective immune responses (1, 2). Lasting protection against infectious diseases induced by vaccines largely depends on adjuvants as well as antigen selection (3–5). For example, past epidemics and pandemics have demonstrated that influenza viruses are continuously evolving, and antigenic drift and shift of surface glycoproteins, including hemagglutinin (HA) and neuraminidase (NA), cause mismatches between vaccine containing strains and circulating strains (1, 2). Typically, existing licensed seasonal vaccines contain 3 or 4 strains of inactivated influenza virus including H1N1, H3N2 and influenza B viruses, and afford only a limited protection (1, 2, 6, 7). However, the gap in protection cannot be exclusively attributed to the antigenic component as most vaccines only contain aluminum salts, such as Al(OH)_3_ and AlPO_4_, as the adjuvant and preferentially enhance humoral responses to the major surface protein HA (3, 8). These adjuvants also skew away from balanced T helper (Th) 1 and 2 responses to a Th2 predominant response that is mediated by induction of antigen specific IgG1 (9), Furthermore, the thermostability of aluminum adjuvant may not be robust (10). Newer vaccines with greater potency like FLUAD, which uses a squalene-based adjuvant microfluidized emulsion 59 (MF59) (11, 12), induced long term and broadly reactive antibodies against HAs (11, 13). Adjuvant System 04 (ASO4) and AS01 containing 3-O-desacyl-4′-monophosphoryl lipid A (MPL) are used in the vaccine against human paplillomavirus and varicella zoster virus infections, respectively, and are reported to induce long lasting effective protection (14, 15). Although these adjuvants have been demonstrated to be useful in high risk groups such as the elderly (11), mild to moderate adverse effects were reported, including pain and bruising at the injection site, as well as muscular ache (13). Also, MPL is chemically modified from biologically derived lipid A from *Salmonella minnesota* R595 lipopolysaccharide, which results in high production cost and variability (16). In addition, many other adjuvants have failed to advance into clinical trials for safety concerns from reactogenicity (17). Hence, it is still of major importance to develop vaccine adjuvants that do not cause adverse effects and that are easy to access at low cost.

Our approach to improve the protective efficacy of vaccines is to activate innate immune cells through multiple receptors thereby simultaneously heightening the responses of antigen presenting cells (APCs) like dendritic cells. In prior work we generated a phospholipid conjugated small molecule TLR7 ligand (TLR-L), **1V270**, which induced robust IgG2a responses in mice (18). In a separate study using a high throughput screen (HTS), pyrimido[5,4-*b*]indoles were identified as NF-κB activators and an initial structure-activity relationship (SAR) study identified a first-in-class small molecule TLR4 agonist, **1Z105** (19). In combination, **1Z105** and **1V270** worked additively as a potent adjuvant for recombinant influenza virus HA protein, inducing rapid and sustained immunity that was protective against influenza viruses in homologous, heterologous, and heterosubtypic murine challenge models (20, 21).

In this study, to further enhance adjuvanticity, we performed further SAR analysis on **1Z105** and identified an 8-(furan-2-yl) substituted pyrimido[5,4-*b*]indole analog (**2B182C**) with greater potency in stimulating mouse and human cells compared to **1Z105**. In a prime-boost preclinical model using inactivated influenza A virus [IIAV; A/California/04/2009 (H1N1)pdm09], and **2B182C** as an adjuvant, increased IgG1 levels against both HA and NA were observed compared to **1Z105**. A liposomal formulation of **1V270** and **2B182C** with IIAV protected mice against lethal homologous virus (H1N1)pdm09 challenge and reduced lung viral titers and cytokine levels. In addition, the liposomal combined adjuvant increased populations of T follicular helper (Tfh) cells, germinal center (GC) B cells, plasmablasts and plasma cells, and B cell receptor diversity in the draining lymph nodes. This combination also induced humoral responses against a broader spectrum of epitopes encompassing HA head and stalk regions and with cross-reactivity against different subtypes of HA and NA with minimal *in vivo* reactogenicity.

## Results

### Structure-Activity Relationship Study of 1Z105 Yields 2B182C

**Figure 1:**
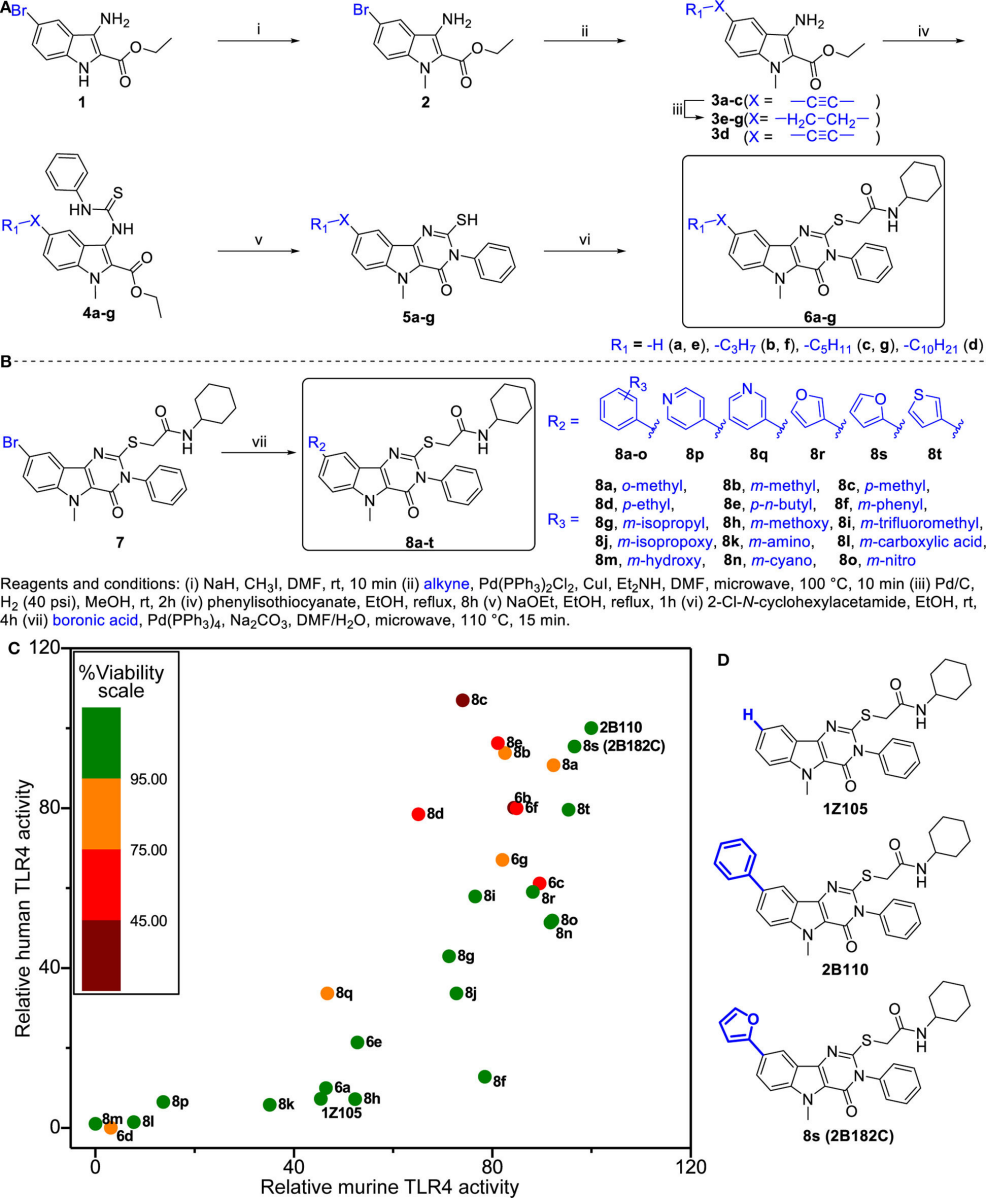
Structure-activity relationship studies in TLR4 agonistic pyrimidoindoles. **(A)** Syntheses of C8-alkynyl and C8-alkyl analogs. **(B)** Syntheses of C8-aryl analogs. **(C)** Scatter plot of hTLR4 and mTLR4 agonistic activity in TLR4-NF-κB HEK reporter cells (HEK-Blue™ hTLR4 and HEK-Blue™ mTLR4, respectively). NF-κB inducible NF-κB SEAP levels in culture supernatants were measured according to manufacturer's protocol. The TLR4 agonistic potency was evaluated as area under the curve (AUC) calculated for dose response curve using 2-fold serial dilutions from 10 μM, and normalized to the AUC for **2B110** as 100% on both axes. AUC of **2B110** for mTLR4 and hTLR4 reporter cells were 28.43 ± 5.49 units and 14.95 ± 1.95, respectively. The color of each point is based on cell viability in TLR4-NF-κB reporter cells for compounds evaluated at 5 μM concentration. Viability data was normalized to vehicle control (Vehicle OD_570−650_ = 0.58 ± 0.01). **(D)** Structures of the lead compounds with differences at C8 substitution highlighted in blue.

We previously synthesized and characterized **1Z105**, *N*-cyclohexyl-2-((5-methyl-4-oxo-3-phenyl-4,5-dihydro-3H-pyrimido[5,4-*b*]indol-2-yl)thio)acetamide (C_25_H_26_N_4_O_2_S), a fully synthetic TLR4 ligand with *in vivo* efficacy as a vaccine adjuvant (19). To further improve the potency of **1Z105**, additional SAR was performed probing the C8 position on the pyrimidoindole scaffold, which was previously found to be tolerant of variation with retained activity as seen with a C8-phenyl substitution (**2B110**) (22). The chemical space was explored with a series of substitutions including various alkyl, alkynyl, aryl, and heteroaryl substitutions (Figure 1A). First, we explored alkyl and alkynyl groups at this position. We introduced a *N*5-methyl initially by methylation of indole analog compound **2** (Figure 1A) as this substituent was previously found to reduce cytotoxicity (22). A “Sonogashira” reaction was then performed to introduce the alkyne on the indole ring (**3a**-**d**). These analogs were then reacted with phenylisothiocyanate to obtain thiourea analogs (**4a**-**d)**, followed by ring closing condensation using sodium ethoxide to obtain pyrimidoindole ring compounds (**5a**-**d**). The S-alkylation of these compounds provided compounds (**6a**-**d**). In parallel, alkynes **3a**-**c** were reduced using hydrogenation to obtain alkyl substituted compounds (**3e**-**g**). These alkyl bearing analogs were further processed as discussed earlier to obtain C8-alkyl analogs **6e**-**g** (Figure 1A). For the next set of compounds, the advanced intermediate **7** (22) was reacted with several different aryl boronic acids via a “Suzuki” coupling reaction to obtain a series of C8-aryl compounds (**8a**-**t**) (Figure 1B).

Titration curves of **1Z105** and derivatives were used to assess their potency in stimulating human and mouse TLR4 response using HEK NF-κB reporter cells (HEK-Blue™ hTLR4 and mTLR4, respectively). The areas under the curve for dose response curves relative to vehicle (0.5% DMSO) were normalized to the greatest activity as 100% of **2B110** for both assays (Figure 1C). The compounds were also tested for *in vitro* toxicity by MTT assay (Figure 1C, Supplementary Figure 1). Among the C8-alkynyl compounds, the 6-carbon chain analog **6b** showed the highest level of TLR4 activation, whereas compounds with shorter chain length (2 carbon chain, **6a**) or compounds with greater chain lengths (8-carbon chain, **6c**) showed a reduction in TLR4 activity, and the 12-carbon chain analog **6d** was completely inactive. These compounds had a relatively high toxicity in cell-based assay suggesting C8-alkynyl substitution was not ideal. The C8-alkyl substituted compounds (**6e-g**) showed a similar trend in TLR4 activity and the toxicity of this set of compounds improved compared to that of the corresponding C8-alkynyl compounds.

Moving on to the C8-aryl substituted compounds, we began first with small modifications to the structure of **2B110** starting with *ortho, meta*, and *para*-methyl substituted compounds **8a**-**c**, respectively. While all these compounds showed potent TLR4 agonistic activities in both mTLR4 and hTLR4 reporter cells, these compounds were relatively toxic. We continued probing the length of alkyl substitution at the *para* position to obtain *p*-ethyl and *p*-*n*-butyl compounds **8d** and **8e**, respectively. However, both these compounds showed relatively high toxicity, while maintaining TLR4 activity. These results on the *para*-substituted compounds prompted us to further explore the *meta* substituted compounds as availability of *ortho*-substituted boronic acids were limited likely due to steric hindrance. The first set of compounds included non-ionic hydrophobic substituents such as *m*-phenyl, *m*-isopropyl, and *m*-trifluoromethyl substituted analogs **8f**, **8g**, and **8i**, respectively. In parallel, we also synthesized *m*-methoxy (**8h**) and *m*-isopropoxy (**8j**) analogs. Most of these compounds retained murine TLR4 activity. However, bulky *m*-phenyl bearing compound **8f** and hydrogen bond accepting *m*-methoxy substituted compound **8h** showed dramatic loss of human TLR4 activity.

Continuing the SAR, we synthesized polar hydrophilic substituent bearing analogs including *m*-amino (**8k**), *m*-carboxylic acid (**8l**), and *m*-hydroxy (**8m**). However, all of these compounds showed significant loss of both human and murine TLR4 activity, suggesting that the binding pocket in TLR4-MD2 does not tolerate hydrophilic substituents at the C8 position. We then introduced hydrogen bond-accepting hydrophobic substituents to obtain the *m*-cyano and *m*-nitro substituted analogs **8n** and **8o**, respectively. While these compounds retained murine TLR4 activity, they were less potent than **2B110** in hTLR4 reporter cells (Figure 1C). The final set of C-8 aryl substituted compounds included bioisosteric replacements of C8-phenyl with 4-pyridyl (**8p**), 3-pyridyl (**8q**), 3-furyl (**8r**), 2-furyl (**8s**), and 3-thienyl (**8t**). While the pyridyl substituted compounds **8p**-**q** were either inactive or toxic, the five membered ring analogs showed potent TLR4 activity in both reporter cells with compound **8s** showing potency equivalent to that of compound **2B110** (Figure 1C). Thus, these SAR studies pointed us to our lead compound **8s** (herein designated as **2B182C**) along with previous lead **1Z105** and **2B110**, the structures of which are shown in Figure 1D. Among the SAR compounds, **2B182C** and **2B110** derivatives had high TLR4 stimulatory potency in both mTLR4 and hTLR4 reporter cells. However, **2B110** had slightly higher toxicity in MTT assays than did **2B182C** (Supplementary Figure 1); hence **2B182C** was chosen as a lead compound for further analyses and **2B110** was held in reserve as an alternate.

**Figure 2:**
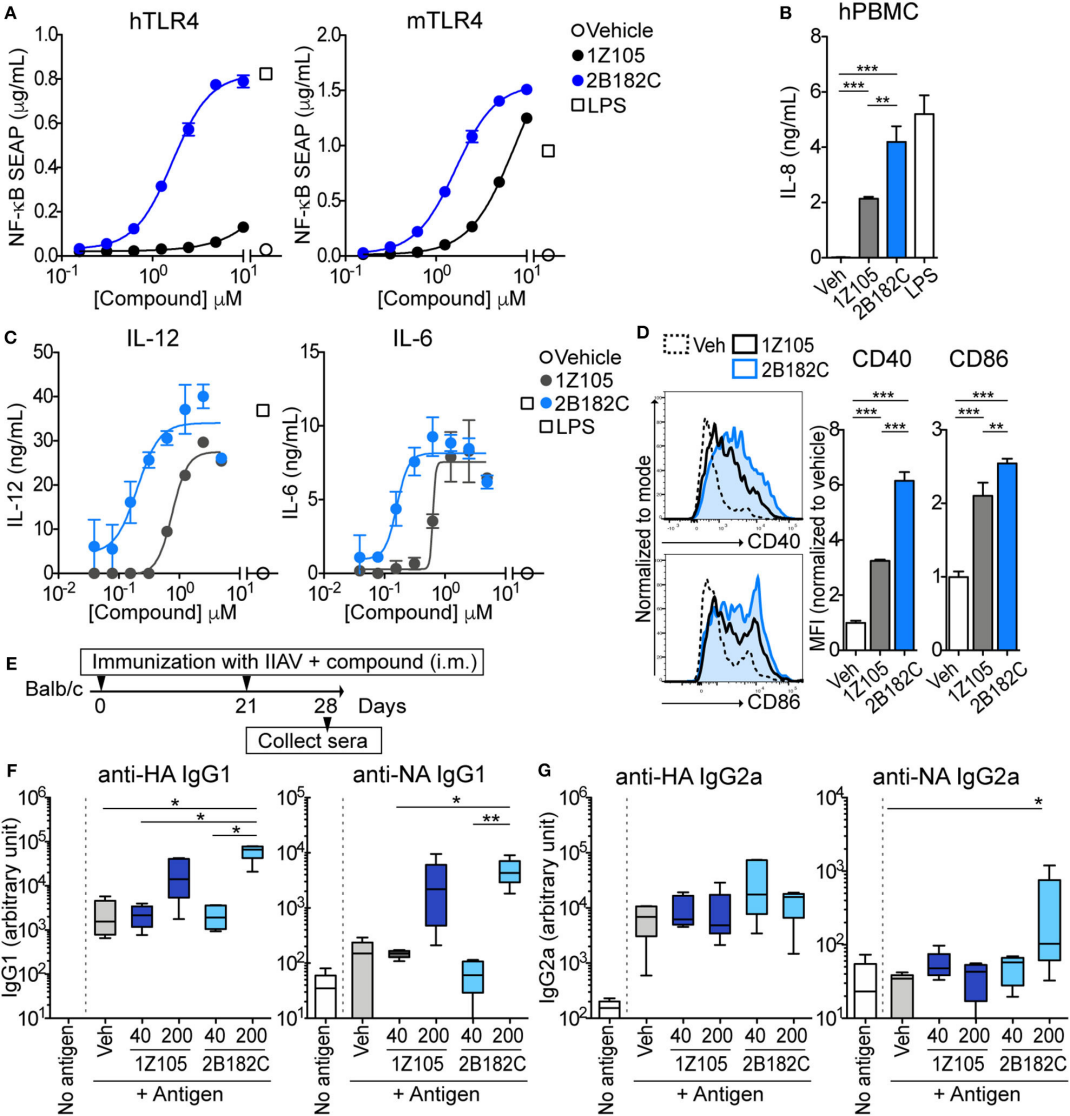
**2B182C** is more potent than **1Z105** in both human and mouse cells and induced higher levels of anti-NA IgG2a. **(A)** TLR4-NF-κB HEK reporter cells (HEK-Blue™ hTLR4 and HEK-Blue™ mTLR4) were treated with compounds **1Z105** and **2B182C** (2-fold serial dilutions from 10 μM) for 20 h. NF-κB inducible NF-κB SEAP levels in culture supernatants were measured according to manufacturer's protocol. EC_50_ of **1Z105** and **2B182C** were 1,445 and 1.66 μM, respectively, on hTLR4 reporter cells. EC_50_ of **1Z105** and **2B182C** were 7.49 and 1.65 μM, respectively on mTLR4 reporter cells. Data represent means ± SD and are representative of two independent experiments with similar results. **(B)** IL-8 release from hPBMC. Data shown are means of triplicates ± SD from one donor and are representative of two donors with similar results. **(C)** IL-12 and IL-6 production levels in BMDCs. EC_50_ of **1Z105** and **2B182C** were 0.77 and 0.20 μM for IL-12, and 0.63 and 0.12 μM for IL-6, respectively. **(D)** Expression levels of costimulatory molecules CD40 and CD86 on BMDCs. Mean fluorescence intensity of CD40 and CD86 were analyzed by flow cytometry. **(B,D)** **P < 0.01, ***P < 0.0001, one-way ANOVA with Tukey's *post-hoc* test. **(A–D)** Data shown are means ± SD. **(E)** Experimental protocol for comparison of two TLR agonists **1Z105** and **2B182C**. BALB/c mice (*n* = 5/group) were i.m. immunized with IIAV (10 μg/injection) plus a TLR4 agonist **1Z105** or **2B182C** (40 and 200 nmol/injection) on days 0 and 21, and were bled on day 28. The sera were evaluated for IgG1 **(F)** and IgG2a **(G)** against hemagglutinin (HA) and neuraminidase (NA) by ELISA. Ten percentage DMSO was used as vehicle. In each box plot, the line within the box represents the median, the bounds are the upper and lower quartiles and the bars indicate minimum and maximum values. Data shown are means ± SEM. *P < 0.05, **P < 0.01, Kruskal-Wallis test with Dunn's *post-hoc* test.

The relative potency of **2B182C** was compared to that of **1Z105** in a series of *in vitro* tests with TLR4 reporter cells and primary human and mouse cells (Figure 2). Notably **2B182C** was 4-fold more potent than **1Z105** (EC_50_ =1.65 μM and EC_50_ =7.49 μM, respectively) in stimulating a mTLR4 reporter line. However, **2B182C** was approximately 800-fold more potent in stimulating the hTLR4 reporter cells than **1Z105** (Figure 2A). This activity was confirmed in human and mouse primary cells, peripheral blood mononuclear cells (hPBMCs) and mouse bone marrow derived dendritic cells (mBMDCs). **2B182C** induced higher level of IL-8 secretion by hPBMCs compared to **1Z105** (Figure 2B). **2B182C** effectively induced cytokine production in primary mBMDCs at relatively low concentrations: IL-12 (EC_50_ = 0.20 μM) and IL-6 (EC_50_ = 0.16 μM; Figure 2C).

As a vaccine adjuvant, stimulating dendritic cells to mature into antigen-presenting cells is critical. Hence, we tested expression levels of co-stimulatory molecules CD40 and CD86 on BMDC using flow cytometry as measures of APC maturation. In mBMDCs, 1 μM **2B182C** induced significantly higher expression levels of costimulatory molecules CD40 and CD86 compared to **1Z105** (Figure 2D). These data confirmed that the SAR study successfully yielded a derivative, **2B182C**, with higher TLR4 stimulatory potency, especially for human TLR4.

### TLR4 Agonist 2B182C Enhances Antigen Specific IgG1 Production

Our previous report demonstrated that TLR4-L **1Z105** and TLR7-L **1V270** separately induced antigen specific Th2-mediated IgG1, and Th1-mediated IgG2a levels when administered as an adjuvant with antigen in mice (20, 21). Since **2B182C** demonstrated higher potency compared to **1Z105** *in vitro*, we first tested whether a vaccine formulated in 10% DMSO and adjuvanted with **2B182C** induced higher levels of antigen specific IgG secretion compared to that induced by a **1Z105** adjuvanted vaccine. BALB/c mice were intramuscularly (i.m.) immunized with IIAV [A/California/04/2009 (H1N1)pdm09] mixed with **1Z105** or **2B182C** at 40 or 200 nmol/injection on days 0 and 21. The mice were bled on day 28, and sera were evaluated by ELISA for antibodies (IgG1 and IgG2a) against two glycoproteins on the surface of the virus, hemagglutinin (HA) and neuraminidase (NA; Figure 2E). At a dose of 200 nmol/injection **2B182C** significantly increased IgG1 antibody production against both HA and NA compared to the vehicle control group (Figure 2F). Interestingly, 200 nmol/injection of **2B182C**, but not **1Z105**, enhanced anti-NA specific IgG2a compared to the vehicle control (Figure 2G).

### 2B182C Enhances Antigen Specific IgG2a Production Induced by 1V270

**Figure 3:**
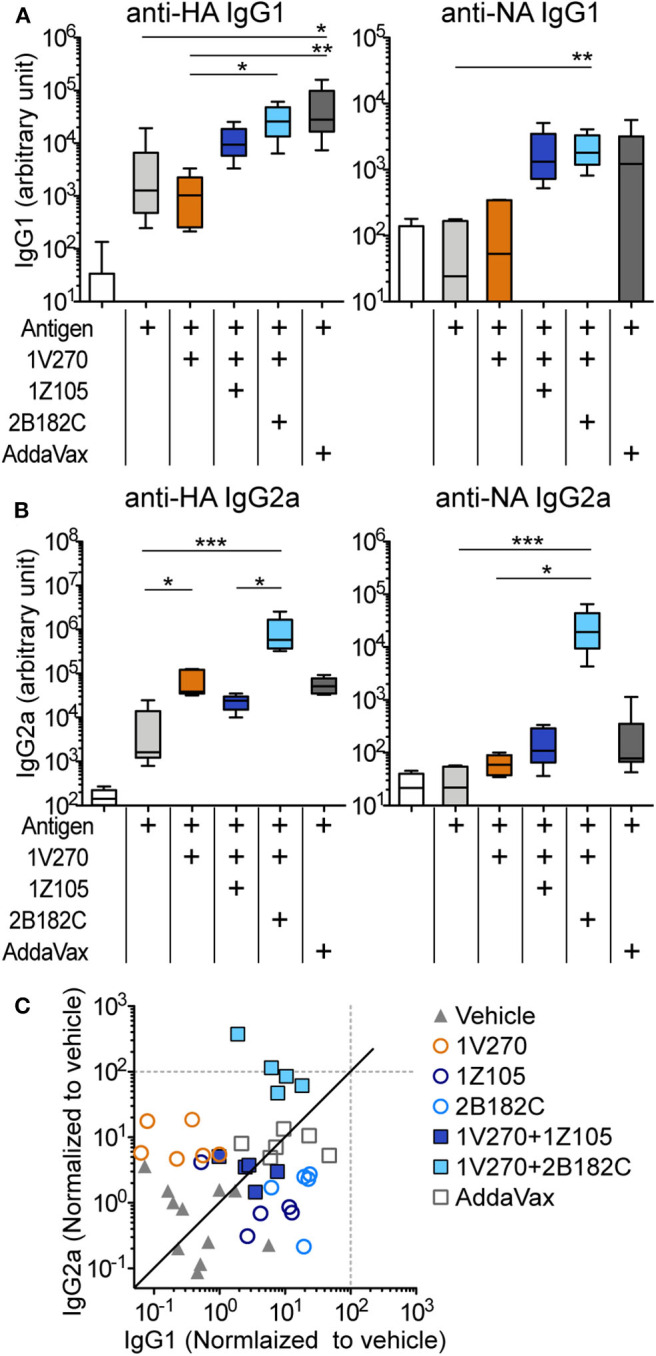
**2B182C** (TLR4-L) enhanced antigen specific IgG2a production induced by **1V270** (TLR7-L). **(A–C)** BALB/c mice (*n* = 5–6) were immunized with IIAV (10 μg/injection) and adjuvants (1 nmol/injection **1V270**, 200 nmol/injection **1Z105** or 200 nmol/injection **2B182C**) and bled on day 28 as shown in Figure 2E. AddaVax™ was used as a positive control. Sera anti-HA and anti-NA IgG1 **(A)**, and IgG2a **(B)** were determined by ELISA. In each box plot, the line within the box represents the median, the bounds are the upper and lower quartiles and the bars indicate minimum and maximum values. *P < 0.05, **P < 0.01, ***P < 0.001, Kruskal-Wallis test with Dunn's *post-hoc* test. **(C)** Anti-HA IgG1 and IgG2a levels induced by all combination treatments (normalized to vehicle) are shown. Data for **1Z105**, **2B182C** alone (200 nmol/injection) and vehicle from Figure 2 are also shown in **(C)**. Each dot indicates an individual mouse. Identity line in solid black.

In our previous studies, the combination adjuvant with the TLR4-L **1Z105** and the TLR7-L **1V270** worked additively with a recombinant HA to induce rapid, long-lasting, and balanced Th1- and Th2-type immunity (20, 21). Thus, we next compared effects of the two TLR4 ligands as co-adjuvants in combination with **1V270** on antibody production using IIAV as an antigen (Figure 3). BALB/c mice were i.m. immunized with IIAV mixed with **1V270** (1 nmol/injection) alone or mixed with **1V270** and **2B182C** (200 nmol/injection) or **1Z105** (200 nmol/injection) on days 0 and day 21. Immunization with **1V270+2B182C** increased IgG1 specific for HA and NA to a similar degree as **1V270+1Z105**, when compared to **1V270** alone (Figure 3A). In contrast, **1V270+2B182C** notably enhanced induction of anti-HA and anti-NA IgG2a compared to **1V270** alone and **1V270+1Z105** (Figure 3B). Hence, immunization with **1V270+1Z105** produced both Th1 and Th2 associated IgG2a and IgG1 responses, respectively, as previously reported (20). However, **1V270+2B182C** augmented both Th1 and Th2 associated humoral responses resulting in an overall skewing toward a relatively Th1 biased response (Figure 3C).

### Liposomal Formulation Enhances Immunostimulatory Effects of the Combination 1V270+2B182C Adjuvant With Minimal Reactogenicity

**Figure 4:**
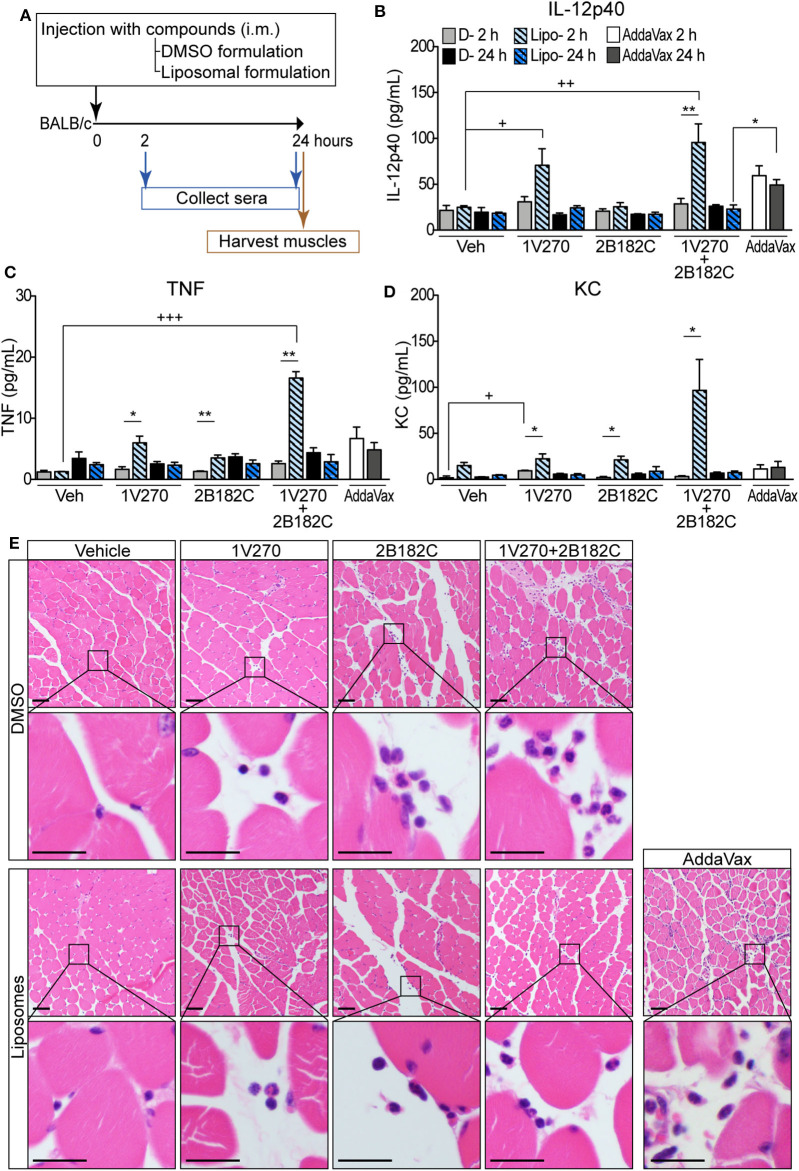
Liposomal formulation enhanced immunostimulatory effects of combination adjuvant with reduced reactogenicity. **(A)** BALB/c mice (*n* = 5/group) were i.m. injected with vehicle, **1V270**, **2B182C**, **1V270+2B182C** with DMSO formulation or liposomal formulation (1 nmol/injection **1V270** and 200 nmol/injection **2B182C** in a volume of 50 μL). AddaVax™ (25 μL/injection) was used as a positive control. Two and 24 h following the injection, sera were collected and examined for **(B)** IL-12p40, **(C)** TNFα, and **(D)** KC levels by Luminex multiplex cytokine assay. Data shown are means ± SEM. *P < 0.05, **P < 0.01, two-tailed Mann-Whitney *U*-test. +*P* < 0.05, ++*P* < 0.01, +++*P* < 0.0001, Kruskal-Wallis with Dunn's *post-hoc* test to compare 4 groups (vehicle, **1V270**, **2B182C**, **1V270+2B182C** in the same formulation). **(E)** Micrographs of H&E stained histological sections of muscles at injected sites (scale bar 50 μm) where squares indicate areas captured at higher magnification in the rows below (scale bar 20 μm).

In order to avoid unwanted cytotoxicity and reactogenicity, while maintaining a robust immune response from a vaccine, adjusting the formulation is an important step in the development of vaccine adjuvants (23). The adjuvant formulation has significant effects on the induction of humoral and cell mediated immune responses in many vaccines (24–26). Therefore, **1V270** and **2B182C** were co-encapsulated in liposomes by Inimmune Corp (Missoula, MT). These liposomal formulations were prepared by the lipid film rehydration method using DOPC:cholesterol in a molar ratio of 2:1, respectively. In the studies above using DMSO formulations, we determined that the optimal ratio of **1V270**:**2B182C** was 1:200 (1 nmol/injection of **1V270** and 200 nmol/injection of **2B182C** in a volume of 50 μL). To evaluate whether the liposomal formulation affected immunostimulatory potency, we examined the *in vivo* systemic and local effects. BALB/c mice were i.m. injected with **1V270** and **2B182C** in DMSO as a control formulation, or in the liposomal (Lipo-) formulation and bled at 2 and 24 h after administration (Figure 4A). Intramuscular injections with Lipo-**1V270**, Lipo-**2B182C**, and Lipo-(**1V270+2B182C**) induced measurable increases of IL-12p40, TNF, and KC levels in serum at 2 h, but these values returned to basal levels by 24 h post injection (Figures 4B–D). Histological analyses of muscles at the injected sites indicated that DMSO-**2B182C** and DMSO-(**1V270+2B182C**) augmented immune cell infiltration, whereas Lipo-**2B182C**, and Lipo-(**1V270+2B182C**) induced minimal cell infiltration (Figure 4E).

### Co-encapsulated Combination Adjuvant Lipo-(1V270+2B182C) Synergistically Enhances Anti-HA and Anti-NA IgG1 and IgG2a Production

**Figure 5:**
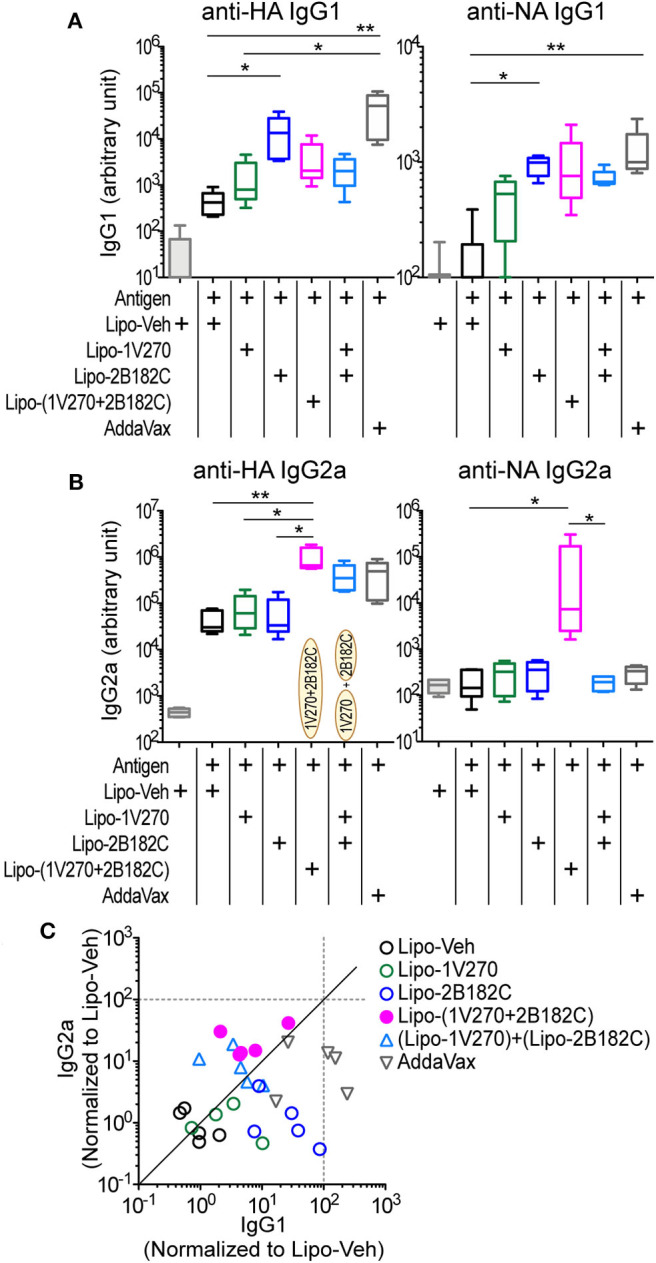
Liposomal **1V270** and **2B182C** synergistically enhanced anti-HA and anti-NA IgG1 and IgG2a production. **(A–C)** BALB/c mice (*n* = 5/group) were i.m. immunized on days 0 and 21 with IIAV (10 μg/injection) with formulated adjuvants as shown in Figure 2E. Liposomal **1V270** (Lipo-**1V270**, 1 nmol/injection), liposomal **2B182C** (Lipo-**2B182C**, 200 nmol/injection), and co-encapsulated liposomal with **1V270** and **2B182C** [Lipo-(**1V270+2B182C**)], and admixed combination with **1V270** and **2B182C** [(Lipo-**1V270**) + (Lipo-**2B182C**)] were injected. Blank liposomes (Lipo-Veh) was used as a control. AddaVax™ was used as a positive control. Sera were collected on day 28 and anti-HA or anti-NA IgG1 **(A)** and IgG2a **(B)** were determined by ELISA. Data shown are means ± SEM and representative of two independent experiments with similar results. *P < 0.05, **P < 0.01, Kruskal-Wallis test with Dunn's *post*-*hoc* test. **(C)** Anti-HA IgG1 and IgG2a levels induced by all combination treatments (normalized to vehicle) are shown. Each dot indicates an individual mouse. Identity line is shown in solid black.

We next evaluated the activity of the combination adjuvant of liposomal **1V270+2B182C** *in vivo* using a prime-boost regimen as depicted in Figure 2E. Two types of combinations of **1V270** and **2B182C** were assessed; a co-encapsulation with **1V270** and **2B182C** in the same liposomes [Lipo-(**1V270+2B182C**)], and an admixed combination of liposomes with individual compounds [(Lipo-**1V270**) + (Lipo-**2B182C**)]. In the prime-boost model, sera harvested on day 28 were assessed for anti-HA and anti-NA antibodies by ELISA (Figure 5). Liposomal **2B182C** (Lipo-**2B182C**) induced higher levels of IgG1, which was consistent with DMSO-**2B182C** (Figure 5A). Unlike DMSO-**1V270**, liposomal **1V270** (Lipo-**1V270**) alone did not enhance IgG2a production (Figure 5B). When the two compounds were combined in the same liposome [Lipo-(**1V270+2B182C**)], anti-NA IgG1, and anti-HA, and anti-NA IgG2a levels were greater than those for mice immunized with Lipo-**1V270**, Lipo-**2B182C**, or control liposome (Lipo-Veh) (Figures 5A,B). Vaccination with the co-encapsulated combination developed antigen specific Th1-biased immune responses (Figure 5C), consistent with the trend observed with the DMSO formulation.

### Lipo-2B182C and Lipo-(1V270+2B182C) Protect Mice Against Homologous Influenza Viral Challenge

**Figure 6:**
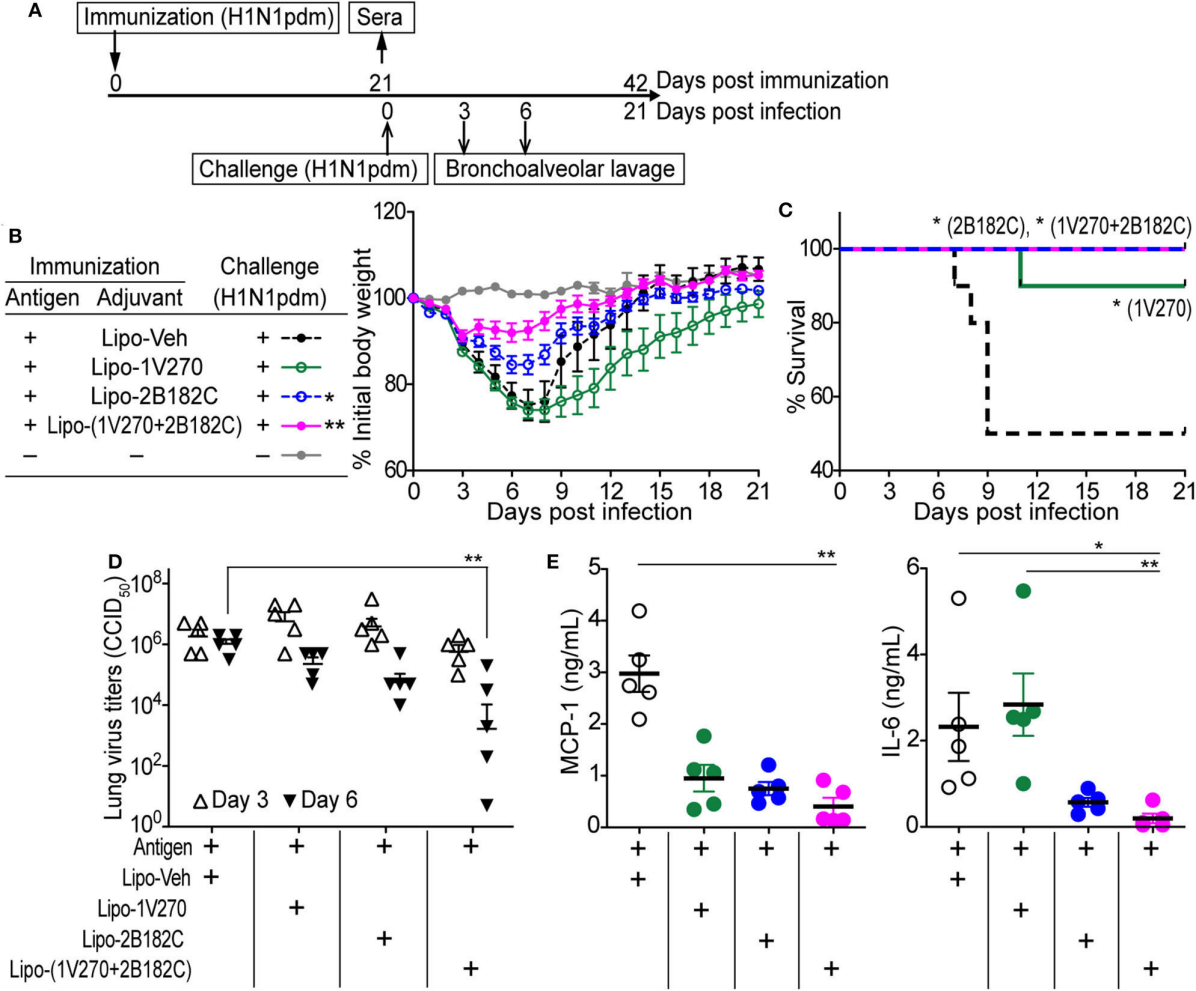
Lipo-**2B182C** and Lipo-**(1V270+2B182C)** protected mice against homologous influenza virus. **(A)** Experimental schedule of homologous influenza virus challenge. BALB/c mice were immunized with IIAV adjuvanted with Lipo-Veh, Lipo-**1V270**, Lipo-**2B182C**, and Lipo-(**1V270+2B182C**) on day 0 and were infected with homologous influenza virus (H1N1)pdm09 on day 21. Body weights and survival were monitored. Three and 6 days after viral challenge, bronchioalveolar lavage samples were collected for lung virus titers and cytokine levels. **(B)** Mean body weight changes after challenge are indicated by % initial body weight (100% = 19.75 ± 0.12 g). *P < 0.05, **P < 0.01, one-way ANOVA with Dunnett's *post-hoc* test compared to Lipo-Veh. **(C)** Survival rates of mice post challenge with homologous virus (H1N1)pdm09. Kaplan-Meier curves with Log-rank test are shown (*P < 0.05). Lung virus titer **(D)** and cytokine levels **(E)** in lung fluids on day 6 were evaluated. *P < 0.05, **P < 0.01, Kruskal-Wallis with Dunn's *post-hoc* test.

To test whether the augmented antibody responses to HA and NA seen with the co-encapsulated combination adjuvant Lipo-(**1V270+2B182C**) could provide immunologic protection against infection, the adjuvant was tested in a mouse adapted influenza lethal challenge model (Figure 6). BALB/c mice were i.m. vaccinated on day 0 with IIAV plus Lipo-**1V270**, Lipo-**2B182C**, or Lipo-(**1V270+2B182C**) and were intranasally challenged with homologous influenza virus (H1N1)pdm09 on day 21 post vaccination (Figure 6A). Body weights and survival were monitored over the next 21 days (Figures 6B,C). Lipo-**2B182C** and Lipo-(**1V270+2B182C**) significantly limited body weight loss after viral challenge (Figure 6B). Furthermore, Lipo-**1V270** showed 90% protection, and Lipo-**2B182C** and Lipo-(**1V270+2B182C**) completely protected mice against homologous influenza virus challenge (Figure 6C).

To evaluate if survival correlates with viral titers in the lung, bronchoalveolar lavage (BAL) was performed on days 3 and 6. Lipo-(**1V270+2B182C**) effectively reduced virus titers in the lungs on day 6 (Figure 6D). Since the levels of cytokine and chemokine in airway epithelial cells (e.g., MCP-1, IL-6, etc) have been correlated with lethal lung injury and pneumonia (27, 28), we evaluated IL-6 and MCP-1 levels in BAL fluids collected on day 6 using the Quansys multiplex ELISA. Use of lipo-(**1V270+2B182C**) as adjuvant significantly reduced MCP-1 and IL-6 production in the airways of infected mice (Figure 6E), suggesting lipo-(**1V270+2B182C**) adjuvanted vaccine prevented lung inflammation due to the reduced viral titers.

### Lipo-(1V270+2B182C) Promotes B Cell Responses in Draining Lymph Nodes

**Figure 7:**
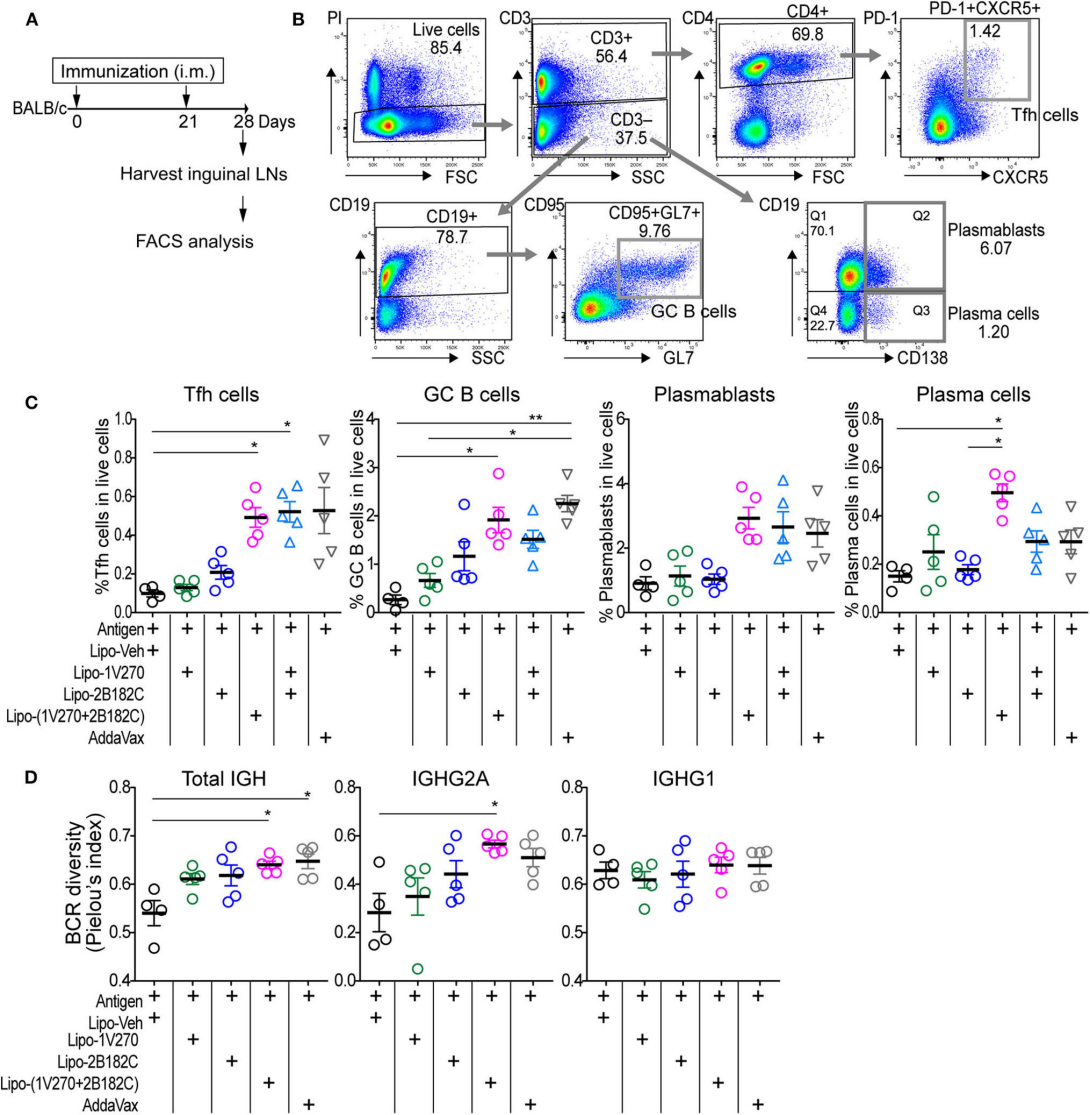
Co-encapsulated combination adjuvant Lipo-**(1V270+2B182C)** increased populations of Tfh cells, antibody secreting cells, and BCR diversity. **(A)** Scheme of immunization strategy. **(B)** BALB/c mice (*n* = 4–5/group) were vaccinated on days 0 and 21 with IIAV (10 μg/injection) with **1V270** (1 nmol/injection) and/or **2B182C** (200 nmol/injection) in a total volume of 50 μL. Twenty-eight days later, lymphocytes in inguinal lymph nodes were harvested for FACS analysis. Gating strategy for Tfh cells (CD3^+^CD4^+^PD-1^+^CXCR5^+^), GC B cells (CD3^−^CD19^+^CD95^+^GL7^+^), plasmablasts (CD3^−^CD19^+^CD138^+^), and plasma cells (CD3^−^CD19^−^CD138^+^) are shown. **(C)** %Tfh cells, GC B cells, plasmablasts, and plasma cells of total live cells. Bars indicate means ± SEM. *P < 0.05, **P < 0.01, Kruskal-Wallis with Dunn's *post-hoc* test. Data are representative of two independent experiment with similar results. **(D)** BALB/c mice (*n* = 4–5/groups) were immunized on days 0 and 21 with IIAV (10 μg /injection) with liposomal adjuvants [**1V270** (1 nmol/injection), **2B182C** (200 nmol/injection) and **1V270+2B182C** (1nmo/injection + 200 nmol/injection)] and draining (inguinal) lymph nodes were harvested on day 28 for BCR repertoire analysis. BCR diversity of total IGH, IGHG1, and IGHG2A are shown by Pielou's index. Bars indicate means ± SEM. *P < 0.05, Kruskal-Wallis with Dunn's post-*hoc test*.

The liposomal combined adjuvant increased the levels of anti-HA and anti-NA antibodies (Figure 5) and protected mice from a lethal challenge by influenza (Figure 6). We further characterized the mechanisms contributing to these beneficial responses. Formation of germinal centers (GC) is essential for long lasting humoral immune responses (29, 30). Naïve B cells differentiate into memory B cells and antibody-secreting plasma cells in GCs in draining lymph nodes. Within the GCs, T follicular helper (Tfh) cells are specialized helper cells that support B cell functions (31, 32). Thus, we investigated whether the liposomal combined adjuvant had an effect on the populations of Tfh cells, GC B cells, plasmablasts, and plasma cells in draining lymph nodes. In the prime-boost immunization protocol described above, lymphocytes in the draining inguinal lymph nodes were harvested on day 28 (1 week after the boost) for analysis by flow cytometry (Figures 7A,B). The percentage of Tfh cells (CD3^+^ CD4^+^ PD-1^+^ CXCR5^+^) was significantly increased in mice vaccinated with Lipo-(**1V270+2B182C**) compared to Lipo-Veh or Lipo-**1V270** (Figure 7C). Additionally, Lipo-(**1V270+2B182C**) injection significantly increased the populations of GC B cells (CD3^−^ CD19^+^ CD95^+^ GL7^+^), plasmablasts (CD3^−^ CD19^+^ CD138^+^), and plasma cells (CD3^−^ CD19^+^ CD138^−^) in the draining lymph nodes compared to Lipo-Veh injected mice (Figure 7C).

### B Cell Receptor Diversities Are Increased After Immunization With 1V270 Plus 2B182C

To examine whether the cellular expansion in the lymph nodes after Lipo-(**1V270+2B182C**) injection was associated with a change in the diversity of the B cell receptors (BCR), we performed next generation sequencing analysis for immunoglobulin heavy chain gene (IGH). Mice were immunized in the prime-boost protocol and lymphocytes in the draining lymph nodes were collected on day 28 (Figure 7A). BCR sequencing analyses showed that the Shannon diversity of total IGH and IGHG2A normalized to total number of clonotypes (Pielou's index) was significantly increased by Lipo-(**1V270+2B182C**), compared to Lipo-Veh (Figure 7D).

### Liposomal Combined Adjuvant, Lipo-(1V270+2B182C), Extends Cross Reactivity of Sera Specific to HA and NA

**Figure 8:**
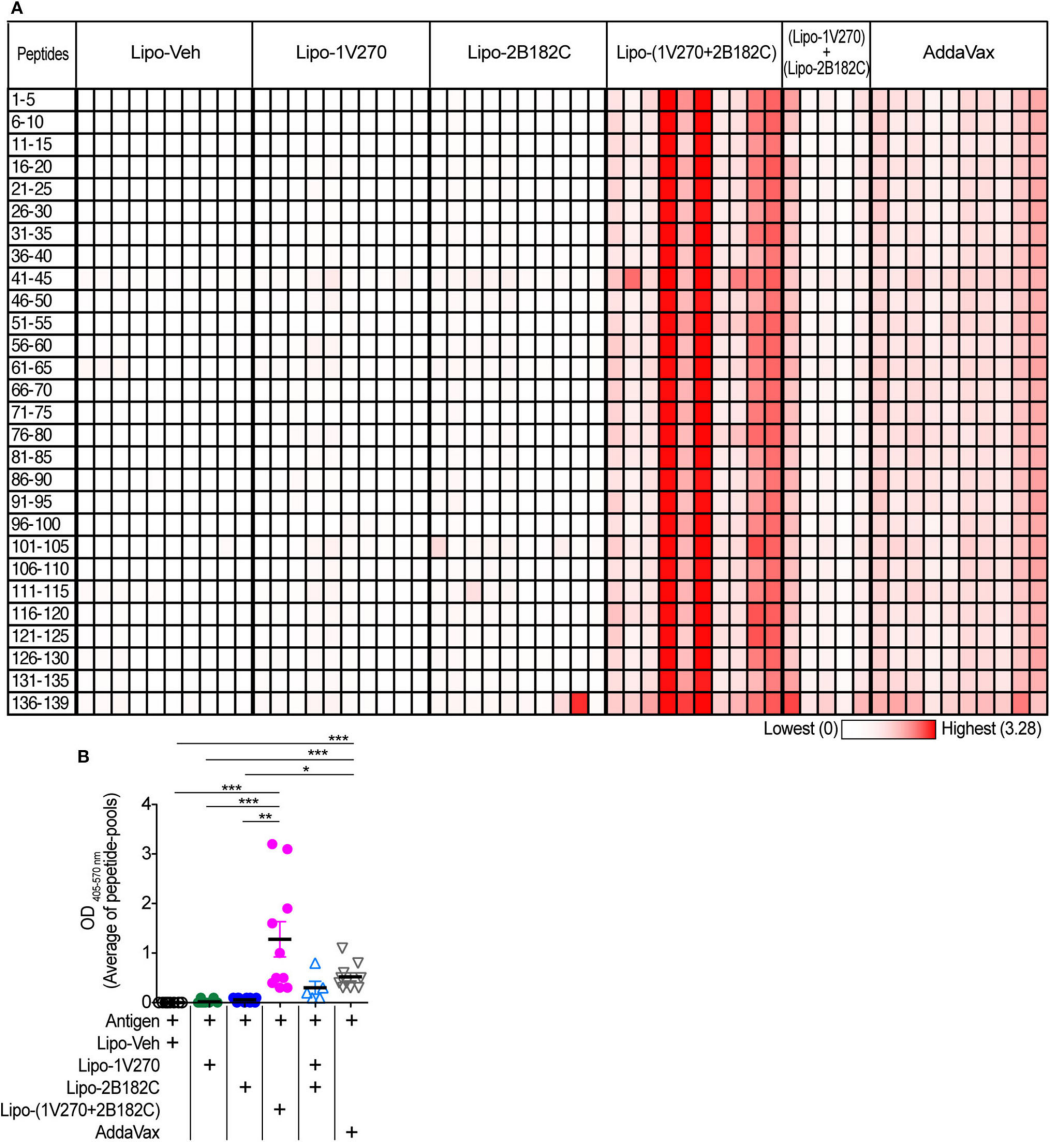
Sera binding profile to A/California/04/2009 (H1N1)pdm09 HA peptide array. **(A,B)** BALB/c mice [*n* = 5 in a group of (Lipo-**1V270**) + (Lipo-**2B182C**), *n* = 10 in other groups)] were immunized with IIAV plus Lipo-Veh, Lipo-**1V270**, Lipo-**2B182C**, Lipo-(**1V270+2B182C**) (co-encapsulated combination), or (Lipo-**1V270**) + (Lipo-**2B182C**) (admixed combination) on days 0 and 21, and were bled on day 28. Peptide arrays of HA of A/California/04/2009 (H1N1)pdm09 were obtained from BEI resources. Pools of 5 peptides per cluster were generated. **(A)** Heatmap of OD _405−570nm_ summarizing antibody binding patterns. Each row and column indicate each peptide pool and mouse, respectively. **(B)** The average absorbance of antibody binding for the 28 peptide pools for individual mice are shown. *P < 0.05, **P < 0.01, ***P < 0.0001, Kruskal-Wallis with Dunn's *post-hoc* test.

Currently approved seasonal influenza vaccines often do not provide broad humoral protection, limiting their effectiveness. Adjuvants that expand the epitopes recognized by the neutralizing antibody response, including less variable regions like the HA stalk protein would provide greater protection against antigenic drift (33–36). Hence we tested the sera from mice by ELISA against pools of peptides derived from the sequence of HA from (H1N1)pdm09. Vaccination with IIAV plus Lipo-(**1V270+2B182C**) promoted broad reactions to peptides of HA and in aggregate the serologic response was greater than that of the other vaccine groups (Figures 8A,B).

**Figure 9:**
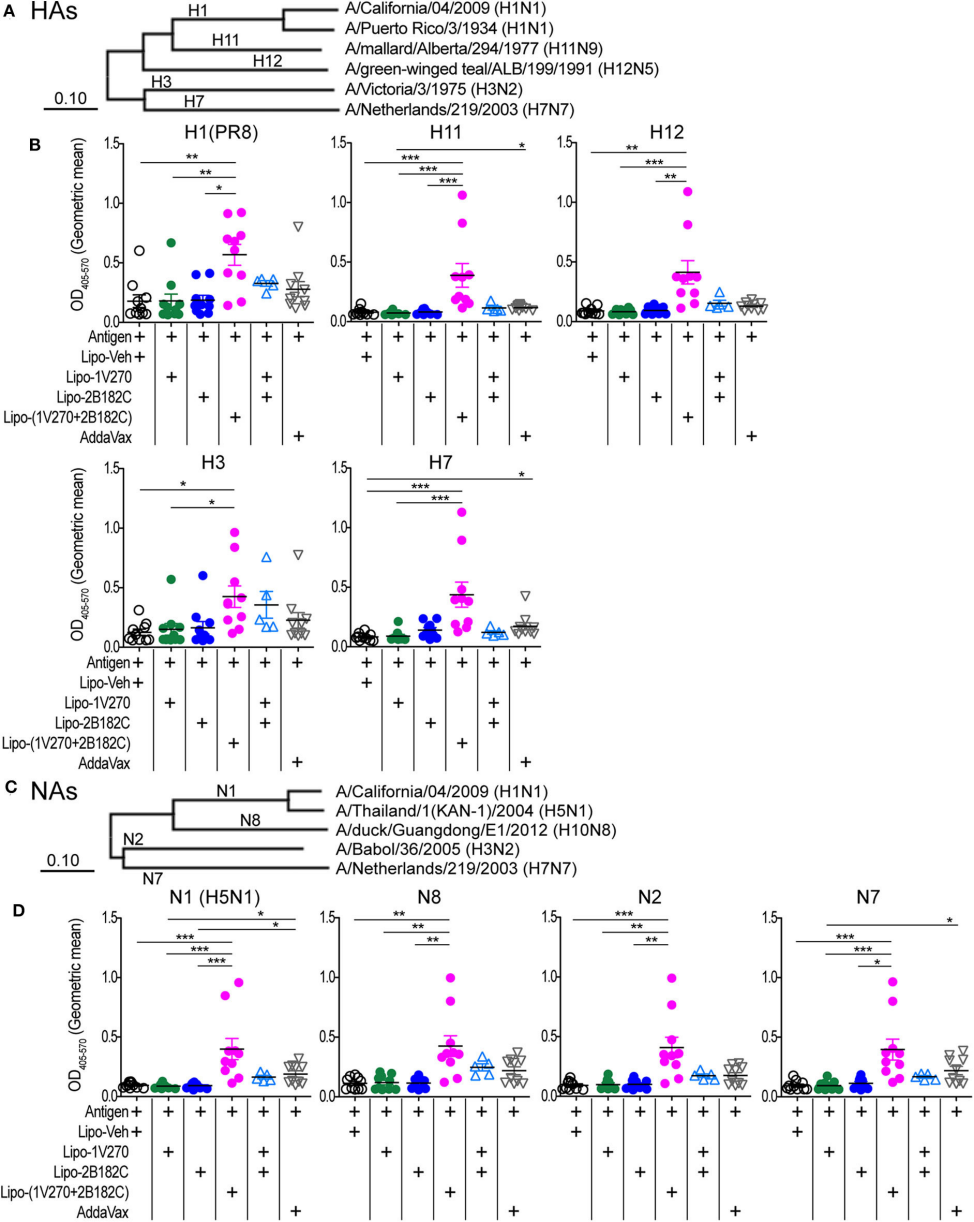
Lipo-(**1V270+2B182C**) induced cross reactive antibodies. **(A–D)** Phylogenetically distinct HAs and NAs of influenza A viruses were tested. **(A,C)** Amino acid sequences of proteins used in ELISA were aligned by the MUSCLE algorithm using the Influenza Research Database. Phylogenetic trees were constructed by the neighbor-joining method using MEGAX software. **(B,D)** BALB/c (*n* = 5–10/group) mice were immunized with IIAV [A/California/04/2009 (H1N1)pdm09] plus Lipo-Veh, Lipo-**1V270**, Lipo-**2B182C**, Lipo-(**1V270+2B182C**), or (Lipo-**1V270**) + (Lipo-**2B182C**) on days 0 and 21 and were bled on day 28. Sera were serially diluted (1:100 to 1:409600) and assessed for total IgG levels against **(B)** HA of PR8 H1N1, H11N9, H12N5, H3N2, and H7N7 and **(D)** NA of H5N1, H10N8, H3N2, and H7N7 by ELISA. Geometric means of total IgG titer curves of individual mice are shown. Total IgG titer curves of HA and NA proteins are shown in Supplementary Figure 2. *P < 0.05, **P < 0.01, ***P < 0.001, Kruskal-Wallis with Dunn's *post-hoc* test.

Given the results above, we further evaluated whether Lipo-(**1V270+2B182C**) could provide broader protection against various subtypes of HA and NA. Cross-reactivity to recombinant HA and NA proteins from antigenically distinct phylogenetic groups were evaluated by ELISA (Figures 9A–D, Supplementary Figures 2A–D). Total IgG levels against the HAs of H1N1, H11N9, H12N5, H3N2, and H7N7 viral subtypes were significantly enhanced by Lipo-(**1V270+2B182C**) compared to liposomes with vehicle (Lipo-Veh) and Lipo-**1V270** (Figure 9B, Supplementary Figure 2B). In addition, serum IgG binding to recombinant NAs of H5N1, H10N8, H3N2, and H7N7 viral subtypes were also increased in mice immunized with Lipo-(**1V270+2B182C**) and the inactivated H1N1 subtype (Figure 9D, Supplementary Figure 2D).

## Discussion

The overall average effectiveness of seasonal influenza vaccines in the past 10 years is ~40% and the highest protection rate reported was 60% in 2010–2011. Furthermore, the effectiveness for high risk groups, such as children (6 months to 18 years old) and elderly subjects, remains low (39 and 25%, respectively) (37–39). Therefore, the development of an adjuvanted influenza vaccine that can provide rapid, broad and sustained protective immunity is still needed. Toll-like receptor ligands (TLRL) have been widely investigated as potential vaccine adjuvants (40) and MPLA, a semi-synthetic TLR4 ligand, has been approved for clinical use (41, 42). However, the use of more than one TLR ligand in combination has been demonstrated to improve the potency of an adjuvant response. Here we demonstrated that further optimization of a vaccine adjuvant with fully synthetic TLR agonists and a co-encapsulated liposomal formulation produced a vaccine that effectively protected mice against lethal homologous influenza virus challenge and reduced lung viral titers and cytokine levels.

The small molecule TLR agonists used in our studies were identified after extensive evaluation of structure-activity relationship (SAR) studies generated for both TLR4 and TLR7 agonists (18, 19, 22). We previously developed a TLR4 agonist, **1Z105**, as a vaccine adjuvant that induced rapid and broad immunity in combination with a TLR7 agonist, **1V270** (20, 21). In this study, we identified a highly potent derivative of this TLR4 agonist, **2B182C**, that strongly enhanced NF-κB activation and cytokine production in both mouse and human primary cells. Both **1V270** and **2B182C** are novel synthetic agonists that are easy to prepare and manufacture in 7-step and 9-step processes, respectively. The TLR4 agonist, **2B182C**, in particular, represents the lead compound identified from a 3rd-generation SAR effort to enhance the potency and broaden the species specificity of the TLR4 activity. The novel finding that substitution at the C8 position of the pyrimidoindole scaffold with an aryl or heteroaryl group provided potent human TLR4 agonistic activity was a critical observation. A major advantage of this TLR4 agonist over other established agonists, such as MPLA, is that it is fully synthetic, easy to prepare and to scale up for clinical use.

In our study the formulation of the combination of synthetic ligands also improved vaccine efficacy. A prime-boost vaccination protocol of IIAV adjuvanted with **2B182C** induced anti-HA and anti-NA IgG1 production and enhanced antigen specific IgG2a production induced by the TLR7 agonist **1V270**. Notably, the co-encapsulation of **1V270** and **2B182C** in a liposome [Lipo-(**1V270+2B182C**)] induced a greater response than that of liposomes with each compound mixed together. In addition, the Lipo-(**1V270+2B182C**) promoted both Th1 and Th2 associated antibody responses against IIAV. The co-encapsulated adjuvant Lipo-(**1V270+2B182C**) also stimulated a germinal center reaction similar to AddaVax, that was associated with increased populations of Tfh cells, GC B cells, plasmablasts, and plasma cells. Therefore, the liposomal combined adjuvant likely enhanced clonal expansion of B cells in the GC.

Antigenic drift of the surface proteins HA and NA allows influenza viruses to escape from the immunosurveillance elicited by annual vaccination. One of the strategies for providing broader protection against viral variation is to create a vaccine that induces humoral responses to relatively conserved stem HA epitopes in the stalk domain (33–36, 43). In this study, immunized IIAV adjuvanted with the co-encapsulated combination Lipo-(**1V270+2B182C**) elevated total IgG levels against multiple clusters of peptides of HA in both head and stalk domains. In contrast, Lipo-**1V270**, Lipo-**2B182C** increased antibody responses to only a few clusters of peptides in each head and stem region. This epitope spreading phenomenon induced by Lipo-(**1V270+B182C**) was consistent with the trend that Lipo-(**1V270+2B182C**) increased the diversity of BCR of B cells in the draining lymph nodes. Since broadly reactive antibodies recognizing HA and NA from multiple viral strains are associated with protection against various influenza virus infection (44–46), this could be one of the critical criteria for a successful vaccine. Therefore, the reactivities of the sera against HA from antigenically distinct phylogenetic groups (H11, H12, H3, and H7) were tested by ELISA. The sera in mice immunized with IIAV (H1N1)pdm09 adjuvanted with the co-encapsulated Lipo-(**1V270+2B182C**) were cross reactive to all the HA subtypes that we tested, suggesting that our novel co-encapsulated adjuvant may effectively support cross-strain protection against lethal infection with this virus.

Since NA is responsible for viral entry and releasing viral particles into cells, inhibition of the NA enzyme has been a primary treatment of influenza [e.g., including oseltamivir (Tamiflu)] (47, 48). Antibodies that bind to NA were suggested to provide durable and broad protection against divergent influenza strains (49–52). It should also be noted that **2B182C** (alone and in combination) enhanced anti-NA IgG2a production, whereas other adjuvants including **1V270** alone and AddaVax did not. Our data demonstrated that the co-encapsulated adjuvant with **1V270** and **2B182C**, Lipo-(**1V270+2B182C**), induced cross reactivity with NAs of other virus strains. A B cell response that targets the major surface glycoprotein NA responses in conjunction with an anti-HA response would broaden host protection (47, 49).

Adjuvants for vaccines often have safety concerns related to solubility and off-target effects of the compounds that may cause reactogenicity and systemic adverse effects (17). Compounds that have strong indications of toxicity or side effects in preclinical studies do not proceed into clinical trials. Thus, a balance of efficacy and reduced reactogenicity of compounds is a key issue in adjuvant development. Liposomes containing DOPC and cholesterol were chosen as both components are biocompatible and have low background immunogenicity. Additionally, this lipid system has been demonstrated to efficiently incorporate various lipidated TLR4 agonists, including MPLA (53), and **1V270**, a lipidated TLR7 agonist (20). Lipo-(**1V270+2B182C**) induced comparable levels of a GC reaction similar to that of AddaVax. There was only a transient increase in IL12, TNF and KC 2 h after injection with Lipo-(**1V270+2B182C**) which resolved back to baseline by 24 h.

In summary, we identified a novel small molecule TLR4 ligand, **2B182C**, which was highly active in both mouse and human APCs. A stable liposomal formulation in combination with a TLR7 ligand (**1V270**) was developed that enhanced anti-HA and anti-NA IgG1 and IgG2a responses after i.m. immunization. The co-encapsulated liposomal formulation of **1V270+2B182C** as an adjuvant for vaccination with IIAV (H1N1)pdm09 protected mice from a homologous viral challenge, reduced lung viral titers and lowered cytokine levels compared to mice that received an unadjuvanted vaccine. Serologic examination showed that the combination adjuvant induced antibodies against a broader spectrum of epitopes encompassing HA head and stalk domains, and with cross-reactivity against different subtypes of HA and NA. These data and the lower reactogenicity suggest that the novel liposomal adjuvant Lipo-(**1V270+2B182C**) might be attractive for development of a universal influenza virus vaccine.

## Methods

### Mice

Female 6–8 week-old BALB/c mice were purchased from Jackson laboratory (Bar Harbor, MA). The mouse experiments using IIAV as an antigen were performed at University of California (UC) San Diego Animal Facility. The influenza challenge studies with live virus were performed by Institute for Antiviral Research, Utah State University using female 6 week-old BALB/c mice (Charles River Laboratories, Wilmington, MA). All animal experiments received prior approval by the Institutional Animal Care and Use Committee (IACUC) for UC San Diego or Utah State University.

### Cells and Reagents

TLR4/NF-κB reporter cell lines HEK-Blue™ humanTLR4 and HEK-Blue™ murineTLR4 cells were purchased from InvivoGen (#hkb-htlr4, # hkb-mtlr4, San Diego, CA). Human PBMC were isolated using Ficoll-Paque Plus (GE Healthcare) from buffy coats. Mouse primary BMDCs were prepared from bone marrow cells harvested from femurs of C57BL/6 mice as previously described (54, 55). Human PBMC and mBMDCs were treated with indicated compounds in RPMI supplemented with 10% FBS (Omega, Tarzana, CA) and penicillin/streptomycin (100 unit/mL/100 μg/mL, Thermo Fisher Scientific, Waltham, MA). AddaVax™ were purchased from InvivoGen (vac-adx-10, San Diego, CA). Inactivated Influenza A virus (IIAV) A/California/04/2009 (H1N1)pdm09 was obtained from BEI resources (# NR-49450, Manassas, VA). TLR7 agonist **1V270** (18), TLR4 agonists **1Z105** (19), and its derivatives including **2B182C** were synthesized in our laboratory. Detailed chemistry is shown in Supplementary Methods.

### Preparation of Liposomal Formulation of 1V270 and 2B182C

**1V270** and **2B182C** were submitted to Inimmune Corp. and liposomal formulation of **1V270** (20 μM), **2B182C** (4 mM), and **1V270+2B182C** (20 μM + 4 mM) was prepared by Inimune corp (Missoula, MT). 1,2-dioleoyl-sn-glycero-3-phosphocholine (DOPC) was purchased from Avanti Polar Lipids (Alabaster, AL), cholesterol was purchased from Sigma (St. Louis, MO). For **2B182C** containing liposomes the adjuvant target concentration was 4 mM and for **1V270** the target concentration was 20 μM. Concentration of each adjuvant was determined by RP-HPLC using a gradient method. The DOPC:cholesterol liposomes were produced with a mass ratio of 60:15 mg/mL, respectively. Liposomes were prepared using the thin-film rehydration method (56). Briefly, lipids were individually dissolved in 9:1 chloroform:methanol to make stocks, and lipid stocks were added to a round bottom flask and mixed. Solvent was evaporated using a Rotavap set to 150 rpm in a water bath at 45–50°C, and residual solvent was removed by storing overnight under reduced pressure at room temperature. Thin films were rehydrated with 10 mM sodium phosphate at pH 7.1. Formulations were sonicated in an Elma 9331 bath sonicator at temperatures below 50°C (above the transition temperature of all compounds) until particle size was reduced below 0.22 μm or the samples appeared opalescent and particle size did not change upon further sonication. Since the presence of endotoxin would cause unwanted TLR4 agonism, all liposomes were made in a BioChemGard biosafety cabinet using aseptic technique, endotoxin-free consumables, and depyrogenated glassware.

### TLR4/NF-κB Reporter Cell Assay

TLR4/NF-κB activation was assessed using HEK-Blue™ hTLR4 and HEK-Blue™ mTLR4 (InvivoGen). The cells were treated with **1Z105** and **2B182C** (2-fold serial dilutions starting from 10 μM) for 20 h. NF-κB inducible secreted embryonic alkaline phosphatase (SEAP) protein in the culture supernatant was measured according to manufacturer's protocol. To evaluate cell viability, 0.5 mg/mL 3-[4,5-dimethylthiazol-2-yl]-2,5-dipheyl tetrazolium bromide (MTT, Thermo Fisher Scientific) solution was added to each well and incubated at 37°C. Six hours later, formazan crystals were lysed with lysis buffer (15% SDS and 0.12% 12 N HCl) and the absorbance was measured at 570 nm using 650 nm as a reference with a plate reader (Tecan, Switzerland).

### Cytokine Assay Using Human and Mouse Primary Cells

Human PBMC (10^6^ cells/mL) were treated with 5 μM **1Z105** and **2B182C** overnight and the culture supernatant assessed for human IL-8 by ELISA. Mouse BMDCs (10^6^ cells/mL) were incubated with serially diluted compounds (2-fold dilution from 5 μM). 0.5% DMSO was used as vehicle. IL-12 and IL-6 levels in the supernatant were measured by ELISA. ELISA was performed as previously described (57). Reagents and dilution factors of antibodies are described in Supplementary Table 1.

### Prime-Boost Vaccination Model

BALB/c mice were i.m. immunized with IIAV (H1N1)pdm09 (10 μg/injection) plus indicated adjuvants in gastrocnemius of hind legs (both legs) on days 0 and 21. IIAV and adjuvants were mixed in a total volume of 50 μL for injection. Details for concentrations of adjuvants and the number of mice in each treatment group are described in each figure legend. Sera were collected on day 28 (1 week after the boost) and evaluated for antigen-specific antibodies. For studies with DMSO formulation, 10% DMSO was used as vehicle. In the experiments using the liposomal-formulated adjuvant, blank liposomes, 1,2-dioleoyl-sn-glycero-3-phosphocholine and cholesterol (DOPC/Chol, control liposomes), was used as vehicle. AddaVax™, which is an oil-in-water adjuvant MF59 (11, 12, 58), was used as a positive control (25 μL/injection).

### Flow Cytometry Analysis

Detailed information for reagents used in flow cytometry analysis are shown in Supplementary Table 2. For co-stimulatory molecules on mBMDCs, mBMDCs (10^6^ cells/mL) were incubated with 1 μM **1Z105** and **2B182C** for 20 h. 0.5% DMSO was uses as vehicle. After removing the supernatant, cells were washed with the stain buffer (BD Biosciences, San Diego, CA) and incubated with anti-mouse CD16/32 antibody for blocking FcR. Cells were then stained with an antibody cocktail with anti-CD40 and anti-CD86 antibodies for 20–30 min at 4°C. For analysis of lymphocytes harvested from draining lymph nodes, two antibody cocktails were prepared; (1) anti-CD3, anti-CD4, anti-PD-1, and anti-CXCR5 antibodies for Tfh cell analysis, (2) anti-CD3, anti-CD19, anti-CD95, anti-CD138, and anti-GL7 antibodies for B cell analysis. After blocking with anti-CD16/CD32 antibody, 2 × 10^6^ cells were stained in each antibody cocktail. For Tfh cell analysis, after staining with primary antibodies, cells were washed with PBS, and incubated with streptavidin-PE. Cells were then washed and stained with propidium iodide to determine live/dead cells. Data were acquired using MACSQuant Analyzer 10 (Miltenyi Biotec, Germany) and analyzed using FlowJo (version 10.6.1, Becton Dickinson, Ashland, OR).

### ELISA for Serum IgG Levels

Reagents used in the ELISA are listed in Supplementary Table 3. ELISA for anti-HA IgG1, anti-NA IgG1, anti-HA IgG2a, and anti-NA IgG2a antibodies (Figures 2–5) were performed as previously described (18) using recombinant HA and NA proteins of A/California/04/2009 (H1N1)pdm09. In brief, plates were coated with HA and NA proteins, blocked, then incubated with sera serially diluted in blocking buffer. An initial dilution of sera was 1:100, followed by 4-fold serial dilutions. After incubation with sera, plates were washed and incubated with detecting antibody, followed by wash and incubation with p-nitrophenyl phosphate substrate. Plates were read at 405 nm on a plate reader (Tecan, Switzerland). For the peptide binding assay, a peptide array of HA of (H1N1)pdm09 was obtained from BEI resources (NR-15433). Sera diluted in 1–200 in blocking buffer were analyzed by peptide ELISA as previously described (59). For evaluation of cross-reactivity of antibody, half-area 96-well-plates were coated with each protein (HAs of H1N1, H11N9, H12N5, H3N2, and H7N7, and NAs of H5N1, H10N8, H3N2, and H7N7), overnight at 4°C. Sera were diluted 1:100 in blocking buffer followed by 1:4 serial dilutions (dilution factors were from 100 to 40,9600). Plates were processed as described above. Phylogenetic relationships of HAs and NAs used in this assay is shown in Figure 9, Supplementary Figure 2. Amino acid sequences of the proteins were aligned by MUSCLE (Multiple Sequence Comparison by Log-Expectation) algorithm (60) using Influenza Research Database (https://www.fludb.org/brc/home.spg?decorator=influenza). Phylogenetic tree was constructed by Neighbor-joining method (61) using Molecular Evolutionary Genetics Analysis software MEGAX (https://www.megasoftware.net/) (62).

### Next-Generation Sequencing for B Cell Receptor Repertoire

The prime-boost model described above was used. Briefly, BALB/c mice were immunized with IIAV (H1N1)pdm09 plus the liposomal formulation of **1V270** and **2B182C** on days 0 and 21. Mice were sacrificed on day 28 and lymphocytes in the draining lymph nodes in the injected sides (inguinal lymph nodes) were collected. The lymphocytes preserved in RNAprotect® Cell Reagent (#76526, QIAGEN) were submitted to Repertoire Genesis Inc. (Osaka, Japan). For BCR analysis, total RNA was extracted from lymphocytes using RNeasy Mini Kit (Qiagen, Hilden, Germany) and the quality of RNA was confirmed by Agilent 2200 TapeStation System (Agilent, Santa Clara, CA). Next-generation sequencing (NGS) was performed with unbiased BCR repertoire analysis technology (Repertoire Genesis Inc., Osaka, Japan) according to the method with some modifications described in previous reports (63, 64). In brief, total RNA was converted to complementary DNA (cDNA) with Superscript III reverse transcriptase (Invitrogen, Carlsbad, CA). Then, double strand (ds)-cDNA was synthesized and an P20EA/P10EA adaptor was ligated to the 5′ end of the ds-cDNA and then cut with *Sph*I restriction enzyme. PCR was performed with KAPA HiFi DNA Polymerase (Kapa Biosystems, Woburn, MA) using P20EA and IgG constant region-specific primer mCG1 (GACAGGGMTCCAKAGTTCC). The second PCR was performed with P20EA and mCG2 (ACYGRCTCAGGGAARTAVCC) using the same PCR conditions. After Tag PCR amplification with mCG-ST1-R (TCGTCGGCAGCGTCAGATGTGTATAAGAGACAGCCYTTGACMAGGCAYCC) and P22EA-ST1-R, index (barcode) sequences were added by amplification with Nextera XT index kit v2 setA (Illumina, San Diego, CA). Sequencing was done with the Illumina Miseq paired-end platform (2 × 300 bp). Data processing, assignment, and data aggregation were automatically performed using repertoire analysis software originally developed by Repertoire Genesis, Inc. Normalized Shannon index (Pielou's index) (65, 66) was calculated based on the number of read of each unique sequence read (abundance data) with vegan 2.5-6 package of R version 3.5.3.

### Evaluation for Protection From Lethal Influenza Virus Challenge

BALB/c mice were i.m. vaccinated with formulated **1V270** and **2B182C** with IIAV (H1N1)pdm09 (3 μg/injection) on day 0 and intranasally challenged with homologous influenza A virus, (H1N1)pdm09 on day 21. Body weight and survival of mice were monitored. The immunization dose of IIAV of 3 μg/injection was determined to be protective for 30–50% of mouse from the challenge with homologous virus was previously determined. For influenza virus challenge, groups of mice were anesthetized by intraperitoneal injection of ketamine/xylazine (50 mg/kg//5 mg/kg) prior to intranasal challenge with 1 × 10^5^ (3 × LD_50_) cell culture infectious doses (CCID_50_) of [(H1N1)pdm09] virus in a 90-μL suspension.

### Virus

Influenza virus (H1N1)pdm09, strain designation 175190 for challenge study, was received from Dr. Elena Govorkova (Department of Infectious Diseases, St. Jude Children's Research Hospital, Memphis TN). The virus was adapted to replication in the lungs of BALB/c mice by 9 sequential passages in mice. Virus was plaque purified in Madin-Darby Canine Kidney (MDCK) cells and a virus stock was prepared by growth in embryonated chicken eggs and then MDCK cells.

### Determination of Lung Virus Titers and Lung Inflammation

Six days after virus challenge, the bronchioalveolar lavage (BAL) procedure was performed immediately after blood collection and was completed within 5–10 min postmortem. A volume of 0.75 mL of PBS was slowly delivered into the lung through the tracheal tube. Immediately after delivery, the fluid was slowly withdrawn by gentle suction and the samples were stored at −80°C. The procedure was repeated a total of three times and lavage fluids from each mouse were pooled. To determine lung virus titers, BAL samples were centrifuged at 2,000 g for 5 min. Varying 10-fold dilutions of BAL supernatants were assayed in triplicate for infectious virus in MDCK cells, with virus titers calculated as described previously (67). For determination of lung cytokine levels, a sample (200 μL) from each lung lavage was tested for MCP-1 and IL-6 using a chemiluminescent multiplex ELISA-based assay according to the manufacturer's instructions (Quansys Biosciences Q-Plex™ Array, Logan, UT).

### Statistical Analyses

Data obtained *in vivo* studies are presented as means with standard error of mean (SEM) and *in vitro* data are shown as means with standard deviation (SD). For *in vitro* data, one-way ANOVA with Tukey's *post-hoc* test was used for multiple comparison. For continuous/ordinal outcomes (antigen specific antibodies, immune cell populations, BCR diversities, lung virus titers, and lung cytokine levels), Kruskal-Wallis tests with Dunn's *post-hoc* test were applied. To compare two groups in mouse experiments, a two-tailed Mann-Whitney test was used. For body weight, last-value-carried-forward approach was used to impute missing values after a mouse was sacrificed, and the average weight over time was used as an outcome for comparison. A log rank test was used to test for a significant difference between Kaplan-Meier survival curves. Prism 5 software (GraphPad Software, San Diego, CA) was used. *P* < 0.05 was considered statistically significant.

## Data Availability Statement

The datasets presented in this study can be found in online repositories. The names of the repository/repositories and accession number(s) can be found below: https://www.ebi.ac.uk/arrayexpress/, E-MTAB-8870.

## Ethics Statement

The animal study was reviewed and approved by University of California San Diego, Institutional Animal Care and Use Committee.

## Author Contributions

FS-K, DC, MCo, and TH designed research, interpreted data and drafted the manuscript. FS-K, SY, FL, JS, and TM conducted experiments. DB and RS formulated compounds in liposomes. TM performed BCR analyses. FS-K, KM, and MP performed statistical analyses. NS, HC, MCh, and PC performed SAR studies including design and syntheses of compounds. All authors contributed to discussions and had opportunity to review and revise the manuscript.

## Conflict of Interest

DB and RS were employed by the company Inimmune Corp. TM was employed by the company Repertoire Genesis Inc. The remaining authors declare that the research was conducted in the absence of any commercial or financial relationships that could be construed as a potential conflict of interest.
