# Supplementary material for: A Novel Synthetic Dual Agonistic Liposomal TLR4/7 Adjuvant Promotes Broad Immune Responses in an Influenza Vaccine With Minimal Reactogenicity
Source: Front Immunol. 2020 Jun 19;11:1207. doi: 10.3389/fimmu.2020.01207 (PMC7318308; doi:10.3389/fimmu.2020.01207)

Supplementary Figure 1

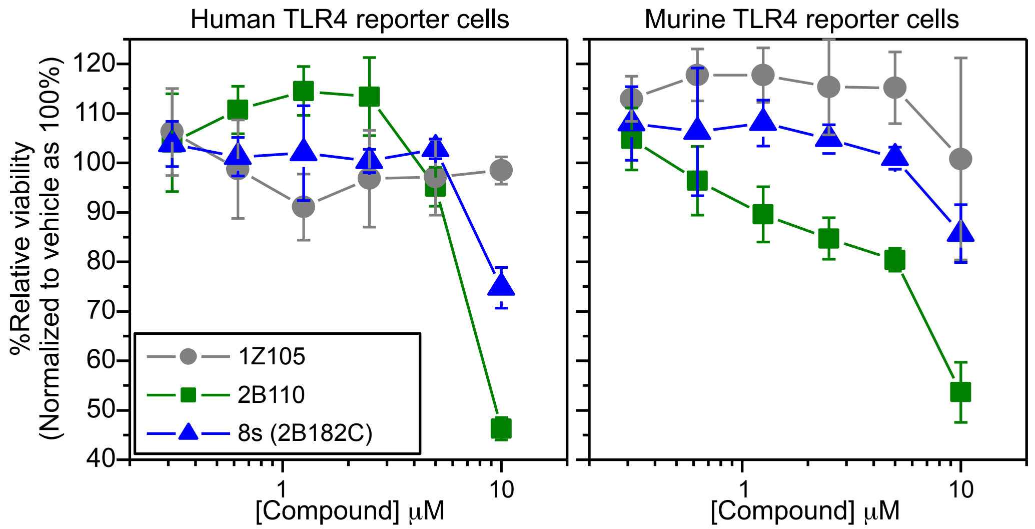

Supplementary Figure 1. Dose response curves for cell viability (toxicity) of lead compounds in human and murine TLR4-NF-κB reporter cells. Cellular toxicity evaluated by MTT assay is presented as % viability normalized to vehicle (0.5% DMSO) as 100%. While **1Z105** had minimal toxicity in both hTLR4 and mTLR4 reporter cells (HEK-Blue™ hTLR4 and HEK-Blue™ mTLR4, respectively), at 10μM **2B110** and compound **8s** (**2B182C**) exhibited toxicity. However, at lower concentrations in mTLR4 reporter cells compounds **2B110** was more toxic compared to **8s**.

Supplementary Figure 2

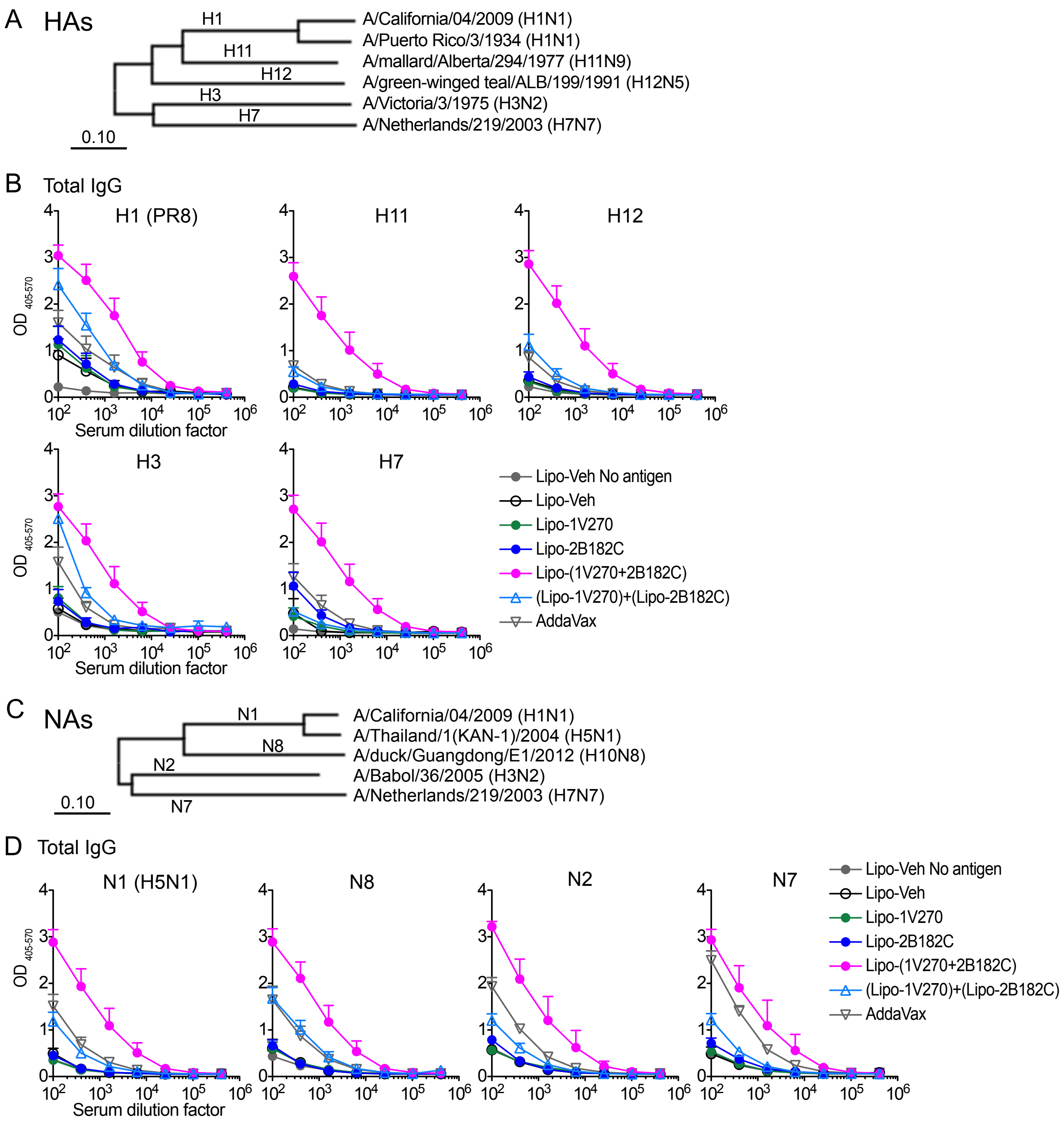

Supplementary Figure 2. ELISA for cross-reactivity of antibodies. (**A-D**) Phylogenetically distinct HA and NAs of influenza A viruses were tested. Phylogenetic relationships of HAs (**A**) and NAs (**C**) of influenza A viruses used in this study. Amino acid sequences of proteins used in ELISA were aligned by the MUSCLE algorithm using the Influenza Research Database. Phylogenetic tree was constructed by the neighbor-joining method using MEGAX software. (**B**) Total IgG titer curves for HAs of H1N1, H11N9, H12N5, H3N2 and H7N7 shown in Figure 9B. (**D**) Total IgG titer curves for NAs of H5N1, H10N8, H3N2, and H7N7 shown in Figure 9D. BALB/c mice (n=5/group) were immunized with IIAV plus Lipo-Veh, Lipo-**1V270**, Lipo-**2B182C**, Lipo-(**1V270+2B182C**), or (Lipo-**1V270**)+(Lipo-**2B182C**) on days 0 and 21, and were bled on day 28. Sera were diluted from 100 to 409600 and total IgG levels were evaluated by ELISA. Data shown are means ± SEM.

Supplemental Table 1. Reagents used in ELISA for hIL-8, mIL-12 and mIL-6

Reagents	Dilution factor	Source	Catalog #
Capture antibodies			
Purified mouse anti-human IL-8	250	BD Biosciences	554716
Purified rat anti-mouse IL-12	200	BD Biosciences	551219
Purified rat anti-mouse IL-6	100	BD Biosciences	554400
Detecting antibodies			
Biotin mouse anti-human IL-8	1000	BD Biosciences	554718
Biotin rat anti-mouse IL-12	1000	BD Biosciences	554476
Biotin rat anti-mouse IL-6	1000	BD Biosciences	554402
Other reagents			
Streptavidin, HRP	1000	Thermo FisherScientific	43-4323
KPL SureBlue^{TM} TMB Peroxidase Substrate		Seracare	5120-0077

Supplemental Table 2. Reagents used in flow cytometry analyses

**Antibodies (clone)**	**Dilution factor**	**Source**	**Catalog #**
Anti-CD86, APC/Cy7 (GL1)	200	BioLegend	105030
Anti-CD40, PE (1C10)	200	eBioscience	12-0401
Anti-CD3, BV510 (145-2C11)	200	BD Biosciences	563024
Anit-CD19, FITC (1D3)	500	BD Biosciences	553785
Anti-CD4, e450 (RM4-5)	1500	eBioscience	48-0042
Anti-CD95, PE/Cy7 (Jo2)	500	BD Biosciences	557653
Anti-CD138, APC (281-2)	200	BD Biosciences	558626
Anti-GL7, Pacific Blue (GL7)	350	BioLegend	144614
Anti-PD-1, APC (J43)	150	BD Biosciences	562671
Anti-CXCR5, Biotin (2G8)	50	BD Biosciences	551960
Anti-CD16/32 (FcR)	300	BD Biosciences	553142
Streptavidin PE	500	BD Biosciences	554061
Propidium Iodide Staining Solution	400	BD Biosciences	556463
Stain buffer		BD Biosciences	554657

Supplemental Table 3. Reagents used in ELISA for IgGs

**Reagents**		**Source**	**Catalog #**
Proteins for coating	*Concentrations*		
Influenza A H1N1 (A/California/04/2009) Hemagglutinin / HA Protein (His Tag)	100 ng/mL	Sino Biological	11055-V08H
Influenza A H1N1 (A/Puerto Rico/8/1934) Hemagglutinin / HA Protein (His Tag)	100 ng/mL	Sino Biological	11684-V08B
Influenza A H3N2 (A/Victoria/3/1975) Hemagglutinin / HA1 Protein (His Tag)	100 ng/mL	Sino Biological	40396-V08H1
Influenza A H7N7 (A/Netherlands/219/2003) Hemagglutinin / HA Protein (His Tag)	100 ng/mL	Sino Biological	11082-V08B
Influenza A H11N9 (A/mallard/Alberta/294/1977) Hemagglutinin / HA Protein (His Tag)	100 ng/mL	Sino Biological	11704-V08H
Influenza A H12N5 (A/green-winged teal/ALB/199/1991) Hemagglutinin / HA Protein (His Tag)	100 ng/mL	Sino Biological	11718-V08H
Influenza A H1N1 (A/California/04/2009) Neuraminidase / NA (Fc Tag)	100 ng/mL	Sino Biological	11058-V07B
Influenza A H5N1 (A/Thailand/1(KAN-1)/2004) Neuraminidase / NA (His Tag)	100 ng/mL	Sino Biological	40064-V07H
Influenza A H3N2 (A/Babol/36/2005) Neuraminidase / NA (His Tag)	100 ng/mL	Sino Biological	40017-V07H
Influenza A H10N8 (A/duck/Guangdong/E1/2012) Neuraminidase / NA Protein (His Tag)	100 ng/mL	Sino Biological	40352-V07B
Influenza A H7N7 (A/Netherlands/219/2003) Neuraminidase / NA Protein (His Tag)	100 ng/mL	Sino Biological	40202-V07H
Antibodies	Dilution factor		
IgG1-AP goat anti-mouse	2000	Southern Biotech	1070-04
IgG2a-AP goat anti-mouse	2000	Southern Biotech	1080-04
IgG-AP goat anti-mouse	2000	Southern Biotech	1030-04
p-Nitrophenyl Phosphate tablets (pNPP)		Sigma	N2770

Supplementary Methods

Chemistry:

Materials. Reagents were purchased as at least reagent grade from commercial vendors unless otherwise specified and used without further purification. Solvents were purchased from Fischer Scientific (Pittsburgh, PA) and were either used as purchased or redistilled with an appropriate drying agent. All the alkyne and boronic acid reagents were purchased from commercially available vendors. Compounds used for structure-activity studies were synthesized according to methods described below, **1Z105** (compound **42** in Reference 1[1]), **2B110** (compound **36** in Reference 2[2]), and advanced intermediate compound **7** (compound **30a** in Reference 2[2]) were synthesized using published literature.[1, 2] All compounds were identified to be at least 95% pure using HPLC.

Instrumentation. Analytical TLC was performed using precoated TLC silica gel 60 F_{254} aluminum sheets purchased from EMD (Gibbstown, NJ) and visualized using UV light. Flash chromatography was carried out using a Biotage Isolera One (Charlotte, NC) system. Microwave reactions were performed using Biotage Initiator+ (Charlotte, NC). Reaction monitoring and purity analysis were done using an Agilent 1260 LC/6420 Triple Quad mass spectrometer (Santa Clara, CA) with Onyx Monolithic C18 (Phenomenex, Torrance, CA) column. Purity of all final compounds was above 95% (also see LC-MS spectra in Supporting Information for all final compounds). The lead compound **2B182C** was analyzed by high resolution MS (HRMS) using an Agilent 6230 ESI-TOFMS (Santa Clara, CA), ^{1}H and ^{13}C NMR spectra were obtained on a Varian 500 with XSens probe (Varian, Inc., Palo Alto, CA). The chemical shifts are expressed in parts per million (ppm) using deuterated DMSO (DMSO-d_6) or $CDCl_3$ as NMR solvents.

Compound 2: Ethyl 3-amino-5-bromo-1-methyl-1*H*-indole-2-carboxylate. Compound **1** (500 mg, 1.77 mmol), sodium hydride (60% dispersion in mineral oil) (71 mg, 1.77 mmol) and DMF (2 mL) were added to a flame dried round bottom flask and stirred at room temperature. Iodomethane (110 µL, 1.77 mmol) was added to the reaction mixture and monitored by LC-MS. Upon completion, solvent was removed, and the residue was extracted with EtOAc. washed with brine and dried over $MgSO_4$. The solvent was then removed, and the resulting crude material was recrystallized in ethanol to give 396.7 mg of compound **2** as light brown solid (yield = 75.6%). ^{1}H NMR (500 MHz, $CDCl_3$) δ 7.68 (d, J = 1.47 Hz, 1H), 7.41 (dd, J = 1.83, 8.93 Hz, 1H), 7.13 (d, J = 9.05 Hz, 1H), 4.81 (br. s., 2H), 4.42 (q, J = 7.09 Hz, 2H), 3.88 (s, 3H), 1.44 (t, J = 7.09 Hz, 3H). MS for $C_{12}H_{14}BrN_2O_2$ $[M + H]^+$ calculated 297.0, found 296.9.

Compound 3b: Ethyl 3-amino-1-methyl-5-(pent-1-yn-1-yl)-1*H*-indole-2-carboxylate. Compound **2** (171 mg, 0.58 mmol), bis(triphenylphosphine)palladium(II) ($Pd(PPh_3)_2Cl_2$, 40 mg, 0.058 mmol), copper(I) iodide (4.38 mg, 0.023 mmol), diethylamine (3 mL), DMF (1 mL) and 1-pentyne (43 mg, 0.63 mmol) were added to a microwave vial and sealed. The vial was then evacuated under vacuum and flushed with argon gas. The reaction mixture was irradiated in a microwave reactor at 100 °C for 10 min. The resultant mixture was then extracted with EtOAc and brine and purified by C18-reverse phase column chromatography (60% MeOH with 0.1% trifluoroacetic acid and 40% water with 0.1% trifluoroacetic acid) to obtain 105 mg of compound **3b** as yellow solid (yield = 72%). ^{1}H NMR (500 MHz, $CDCl_3$) δ 7.63 (s, 1H), 7.37 (d, J = 8.80 Hz, 1H), 7.15 (d, J = 8.80 Hz, 1H), 4.85 (br. s., 2H), 4.41 (q, J = 7.09 Hz, 2H), 3.88 (s, 3H), 2.41 (t, J = 7.09 Hz, 2H), 1.61 - 1.71 (m, 2H), 1.44 (t, J = 7.09 Hz, 3H), 1.07 (t, J = 7.34 Hz, 3H). MS for $C_{17}H_{21}N_2O_2$ $[M + H]^+$ calculated 285.2, found 285.1.

Compounds **3a**, **3c-d** were obtained using the same protocol as for compound **3b** using different alkynes (trimethylsilylacetylene for **3a**, 1-heptyne for **3c**, and 1-dodecyne for **3d**). Compound **3a** was

obtained by an additional reaction step of deprotection of C-TMS group by 1M tetrabutylammonium fluoride (TBAF) solution in THF, followed by purification.

Compound 3f: Ethyl 3-amino-1-methyl-5-pentyl-1*H*-indole-2-carboxylate.
Compound **3b** (71 mg, 0.25 mmol) was subjected to reduction reaction on an Anton-Parr shaker apparatus with a catalytic amount of Pd on carbon (10%), H_2 gas (40 psi) and MeOH for 2h. Upon completion, the reaction mixture was filtered through celite and purified by column chromatography (8% EtOAc and 92% hexanes) to obtain 67 mg of compound **3f** as off-white solid (yield = 94%). ^{1}H NMR (500 MHz, $CDCl_3$) δ 7.32 (s, 1H), 7.14 - 7.22 (m, 2H), 4.83 (br. s., 2H), 4.41 (q, *J* = 7.09 Hz, 2H), 3.87 (s, 3H), 2.68 (t, *J* = 7.70 Hz, 2H), 1.65 (quin, *J* = 7.30 Hz, 2H), 1.44 (t, *J* = 7.21 Hz, 3H), 1.31 - 1.37 (m, 4H), 0.90 (t, *J* = 6.72 Hz, 3H). MS for $C_{17}H_{25}N_2O_2$ $[M + H]^+$ calculated 289.2, found 285.1.

Compounds **3e** and **3g** were obtained using the same protocol as for compound **3f**.

Compound 4b: Ethyl 1-methyl-5-(pent-1-yn-1-yl)-3-(3-phenylthioureido)-1*H*-indole-2-carboxylate.
Compound **3b** (75 mg, 0.26 mmol) was dissolved in ethanol with heat followed by the addition of phenylisothiocyanate (40 mg, 0.29 mmol). The reaction mixture was then heated under reflux with stirring for 8h and allowed to cool overnight. Solids were filtered, washed with ethanol, dried under vacuum to obtain 64.5 mg of compound **4b** as orange solid (yield = 59%). ^{1}H NMR (500 MHz, $CDCl_3$) δ 8.11 (s, 1H), 7.87 (s, 1H), 7.71 (br. s., 1H), 7.42 - 7.48 (m, 3H), 7.32 - 7.41 (m, 3H), 7.25 (t, *J* = 7.30 Hz, 1H), 4.41 (q, *J* = 7.09 Hz, 2H), 4.05 (s, 3H), 2.40 (t, *J* = 6.97 Hz, 2H), 1.65 (sxt, *J* = 7.24 Hz, 2H), 1.40 (t, *J* = 6.97 Hz, 3H), 1.07 (t, *J* = 7.34 Hz, 3H). MS for $C_{24}H_{26}N_3O_2S$ $[M + H]^+$ calculated 289.2, found 288.9.

Compounds **4a**, **4c-g** were obtained using the same protocol as for compound **4b**.

Compound 5b: 2-Mercapto-5-methyl-8-(pent-1-yn-1-yl)-3-phenyl-3,5-dihydro-4*H*-pyrimido[5,4-*b*]indol-4-one.
In a flame dried round bottom flask sodium ethoxide (20 mg, 0.29 mmol) was combined with anhydrous ethanol (1 mL). Separately, in a flame-dried flask, compound **4b** (25 mg, 0.06 mmol) dissolved in anhydrous ethanol was added to the above mixture and refluxed for 3h. The reaction was monitored by LC-MS and on completion, the solvent was removed, and the crude mixture was taken forward to the next step without further purification.

Compounds **5a**, **5c-g** were obtained using the same protocol as for compound **5b**.

Compound 6b: *N*-cyclohexyl-2-((5-methyl-4-oxo-8-(pent-1-yn-1-yl)-3-phenyl-4,5-dihydro-3*H*-pyrimido[5,4-*b*]indol-2-yl)thio)acetamide.
To the crude mixture obtained above of compound **5b** was added 2-chloro-*N*-cyclohexylacetamide (60 mg, 0.34 mmol) and stirred at room temperature until completion. The reaction mixture was then extracted with EtOAc, washed with H_2O and dried over $MgSO_4$. The resulting crude solid was purified by column chromatography (30% EtOAc and 70% hexanes) to obtain 16.9 mg of compound **6b** (yield = 55%).^{1}H NMR (500 MHz, $CDCl_3$) δ 8.17 (s, 1H), 7.56 - 7.62 (m, 4H), 7.40 (d, *J* = 8.56 Hz, 2H), 7.32 - 7.38 (m, 2H), 4.19 (s, 3H), 3.73 - 3.81 (m, 3H), 2.45 (t, *J* = 6.97 Hz, 2H), 1.89 (s, 2H), 1.68 (sxt, *J* = 7.30 Hz, 2H), 1.50 - 1.55 (m, 1H), 1.26 - 1.39 (m, 4H), 1.08 - 1.18 (m, 6H).

^{13}C NMR (126 MHz, $CDCl_3$) δ 167.7, 156.0, 153.8, 139.5, 137.1, 135.4, 131.3, 130.3, 129.9, 129.1, 124.0, 119.9, 119.7, 116.5, 110.2, 89.2, 80.7, 36.2, 32.8, 31.3, 29.7, 25.4, 24.6, 22.3, 21.4, 13.6. MS for $C_{30}H_{33}N_4O_2S$ $[M + H]^+$ calculated 513.2, found 513.1.

Compounds **6a**, **6c-g** were obtained using the same protocol as for compound **6b**.

Compound 8s: *N*-cyclohexyl-2-((8-(furan-2-yl)-5-methyl-4-oxo-3-phenyl-4,5-dihydro-3*H*-pyrimido[5,4-*b*]indol-2-yl)thio)acetamide.
2-Furanylboronic acid (64 mg, 0.57 mmol) and $Pd(PPh_3)_4$ (222 mg, 0.19 mmol) were combined in a microwave flask, sealed, evacuated and flask flushed with argon gas. Compound **7** (250 mg, 0.48 mmol) was dissolved in DMF (4 mL) and added to the reaction flask. Dissolve Na_2CO_3 (153 mg, 1.44 mmol) was dissolved in H_2O (1 mL) and added to reaction mixture and reaction mixture was irradiated in a microwave at 110 °C for 15 min. Solvent was then removed and the residue was dissolved in EtOAc, washed with brine and purified by column chromatography to give compound **8s**.
Compound **8s** (**2B182C**). *N*-cyclohexyl-2-((8-(furan-2-yl)-5-methyl-4-oxo-3-phenyl-4,5-dihydro-3*H*-pyrimido[5,4-*b*]indol-2-yl)thio)acetamide. ^1H NMR (500 MHz, DMSO-d_6) δ 8.42 (s, 1H), 8.29 (d, *J* = 7.83 Hz, 1H), 7.92 (dd, *J* = 1.47, 8.80 Hz, 1H), 7.68 - 7.82 (m, 2H), 7.54 - 7.65 (m, 3H), 7.38 - 7.50 (m, 2H), 6.95 (d, *J* = 3.18 Hz, 1H), 6.64 (dd, *J* = 1.71, 3.18 Hz, 1H), 4.11 (s, 3H), 3.86 (s, 2H), 3.45 - 3.57 (m, 1H), 1.75 (s, 2H), 1.56 - 1.66 (m, 2H), 1.45 - 1.53 (m, 1H), 1.16 - 1.29 (m, 4H), 1.02 - 1.12 (m, 1H). ^{13}C NMR (126 MHz, DMSO-d_6) δ 165.8, 155.3, 153.7, 153.4, 142.4, 139.2, 137.3, 135.9, 130.0, 129.6, 129.6, 124.1, 123.3, 119.9, 119.4, 115.1, 112.2, 111.6, 104.7, 48.1, 36.8, 32.5, 31.3, 25.2, 24.6. HRMS for $C_{29}H_{29}N_4O_3S$ $[M + H]^+$ calculated 513.1955, found 513.1948.

Compounds **8a-r**, **8t** were obtained using the same protocol as for compound **8s** using different boronic acids.

References:

1. Chan, M.; Hayashi, T.; Mathewson, R. D.; Nour, A.; Hayashi, Y.; Yao, S.; Tawatao, R. I.; Crain, B.; Tsigelny, I. F.; Kouznetsova, V. L.; Messer, K.; Pu, M.; Corr, M.; Carson, D. A.; Cottam, H. B. Identification of substituted pyrimido[5,4-b]indoles as selective toll-like receptor 4 ligands. *J Med Chem* **2013,** 56, 4206-4223.
2. Chan, M.; Kakitsubata, Y.; Hayashi, T.; Ahmadi, A.; Yao, S.; Shukla, N. M.; Oyama, S. Y.; Baba, A.; Nguyen, B.; Corr, M.; Suda, Y.; Carson, D. A.; Cottam, H. B.; Wakao, M. Structure-activity relationship studies of pyrimido[5,4-b]indoles as selective toll-like receptor 4 ligands. *J Med Chem* **2017,** 60, 9142-9161.

^{1}H NMR and LC-MS
Compound **2**

4/27/2020 1:01:51 PM

**Formula** $C_{12}H_{13}BrN_2O_2$	**FW** 297.1478

**Acquisition Time (sec)**	2.0486	**Comment**	Std proton	**Date**	Apr 27 2020	**Date Stamp**	Apr 27 2020
**File Name**	C:\Users\Mycoahhh\Documents\NMR\michan\2b188-proton-042720.fid\fid					**Frequency (MHz)**	499.83
**Nucleus**	1H	**Number of Transients**	32	**Original Points Count**	16415	**Points Count**	32768
**Pulse Sequence**	s2pul	**Receiver Gain**	36.00	**Solvent**	CHLOROFORM-d		
**Spectrum Offset (Hz)**	2997.3250	**Spectrum Type**	STANDARD	**Sweep Width (Hz)**	8012.82	**Temperature (degree C)**	30.000

^{1}H NMR (500 MHz, $CDCl_3$) δ 7.68 (d, *J* = 1.47 Hz, 1H), 7.41 (dd, *J* = 1.83, 8.93 Hz, 1H), 7.13 (d, *J* = 9.05 Hz, 1H), 4.81 (br. s., 2H), 4.42 (q, *J* = 7.09 Hz, 2H), 3.88 (s, 3H), 1.44 (t, *J* = 7.09 Hz, 3H)

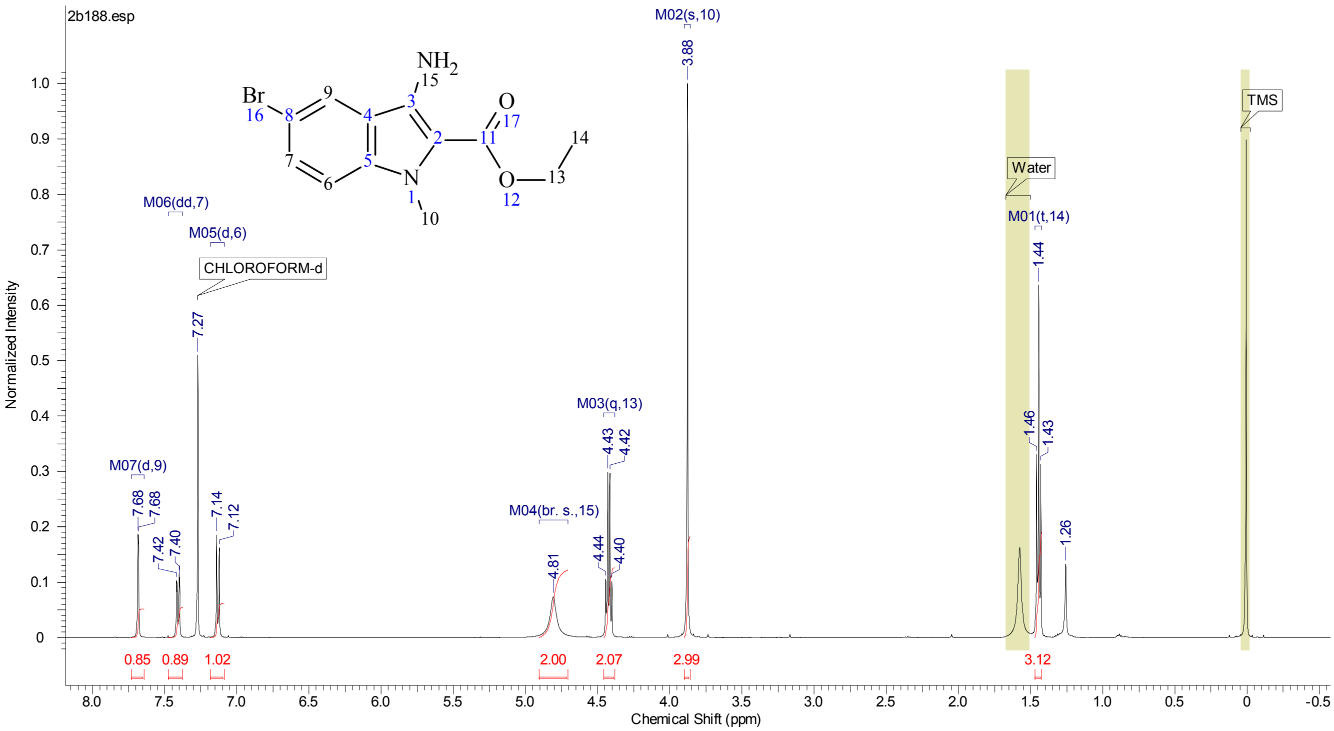

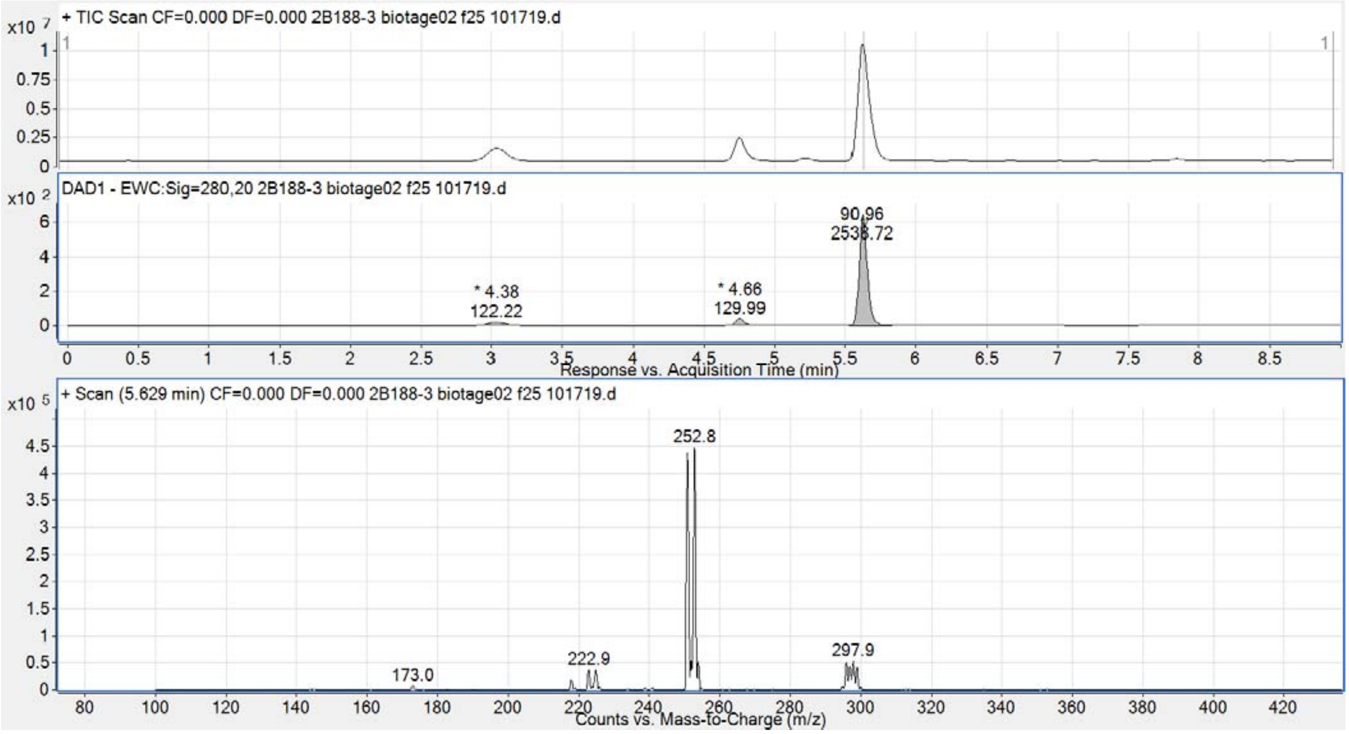

^{1}H NMR and LC-MS

Compound **3b**

4/27/2020 1:04:19 PM

**Formula** $C_{17}H_{20}N_2O_2$	**FW** 284.3529

Acquisition Time (sec)	2.0486	***Comment***	Std proton	***Date***	Apr 27 2020	***Date Stamp***	Apr 27 2020
File Name	C:\Users\Mycoahhh\Documents\NMR\michan\2b191g-proton-042720.fid\fid					***Frequency (MHz)***	499.83
Nucleus	1H	***Number of Transients***	32	***Original Points Count***	16415	***Points Count***	32768
Pulse Sequence	s2pul	***Receiver Gain***	36.00	***Solvent***	CHLOROFORM-d		
Spectrum Offset (Hz)	2997.3250	***Spectrum Type***	STANDARD	***Sweep Width (Hz)***	8012.82	***Temperature (degree C)***	30.000

^{1}H NMR (500 MHz, $CDCl_3$) δ 7.63 (s, 1H), 7.37 (d, *J* = 8.80 Hz, 1H), 7.15 (d, *J* = 8.80 Hz, 1H), 4.85 (br. s., 2H), 4.41 (q, *J* = 7.09 Hz, 2H), 3.88 (s, 3H), 2.41 (t, *J* = 7.09 Hz, 2H), 1.61 - 1.71 (m, 2H), 1.44 (t, *J* = 7.09 Hz, 3H), 1.07 (t, *J* = 7.34 Hz, 3H)

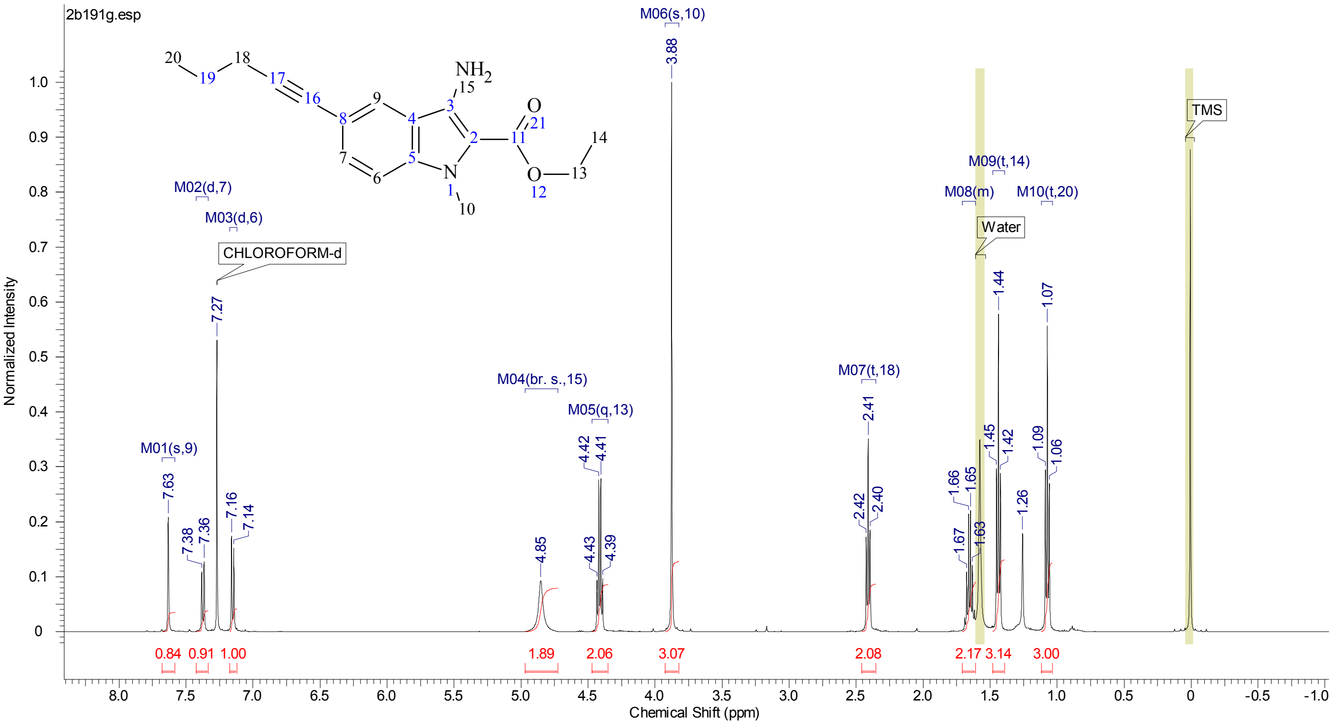

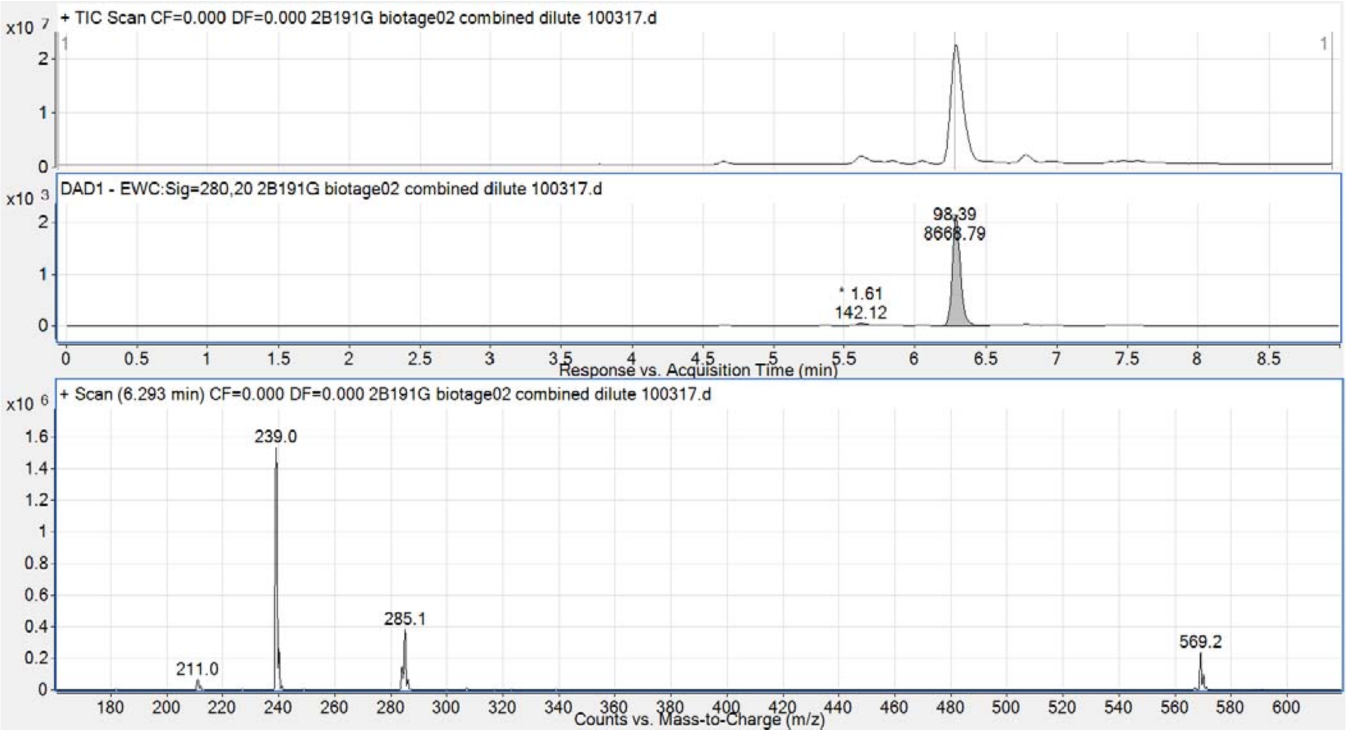

^{1}H NMR and LC-MS

Compound **3f**

4/27/2020 1:04:46 PM

Formula $C_{17}H_{24}N_2O_2$	FW 288.3847

**Acquisition Time (sec)**	2.0486	**Comment**	Std proton	**Date**	Apr 27 2020	**Date Stamp**	Apr 27 2020
**File Name**	C:\Users\Mycoahhh\Documents\NMR\michan\2b201-proton02-042720.fid\fid					**Frequency (MHz)**	499.83
**Nucleus**	1H	**Number of Transients**	64	**Original Points Count**	16415	**Points Count**	32768
**Pulse Sequence**	s2pul	**Receiver Gain**	20.00	**Solvent**	CHLOROFORM-d		
**Spectrum Offset (Hz)**	2997.3250	**Spectrum Type**	STANDARD	**Sweep Width (Hz)**	8012.82	**Temperature (degree C)**	30.000

^{1}H NMR (500 MHz, $CDCl_3$) δ 7.32 (s, 1H), 7.14 - 7.22 (m, 2H), 4.83 (br. s., 2H), 4.41 (q, J = 7.09 Hz, 2H), 3.87 (s, 3H), 2.68 (t, J = 7.70 Hz, 2H), 1.65 (quin, J = 7.30 Hz, 2H), 1.44 (t, J = 7.21 Hz, 3H), 1.31 - 1.37 (m, 4H), 0.90 (t, J = 6.72 Hz, 3H)

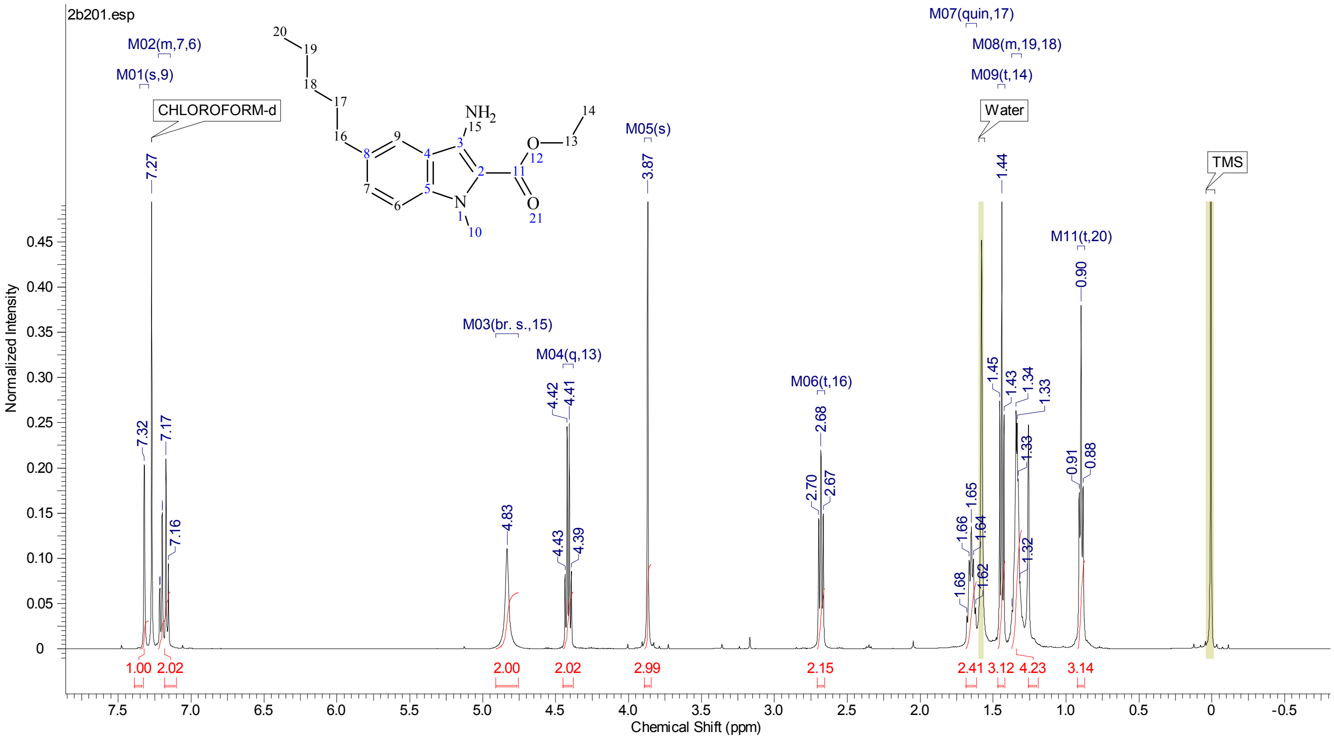

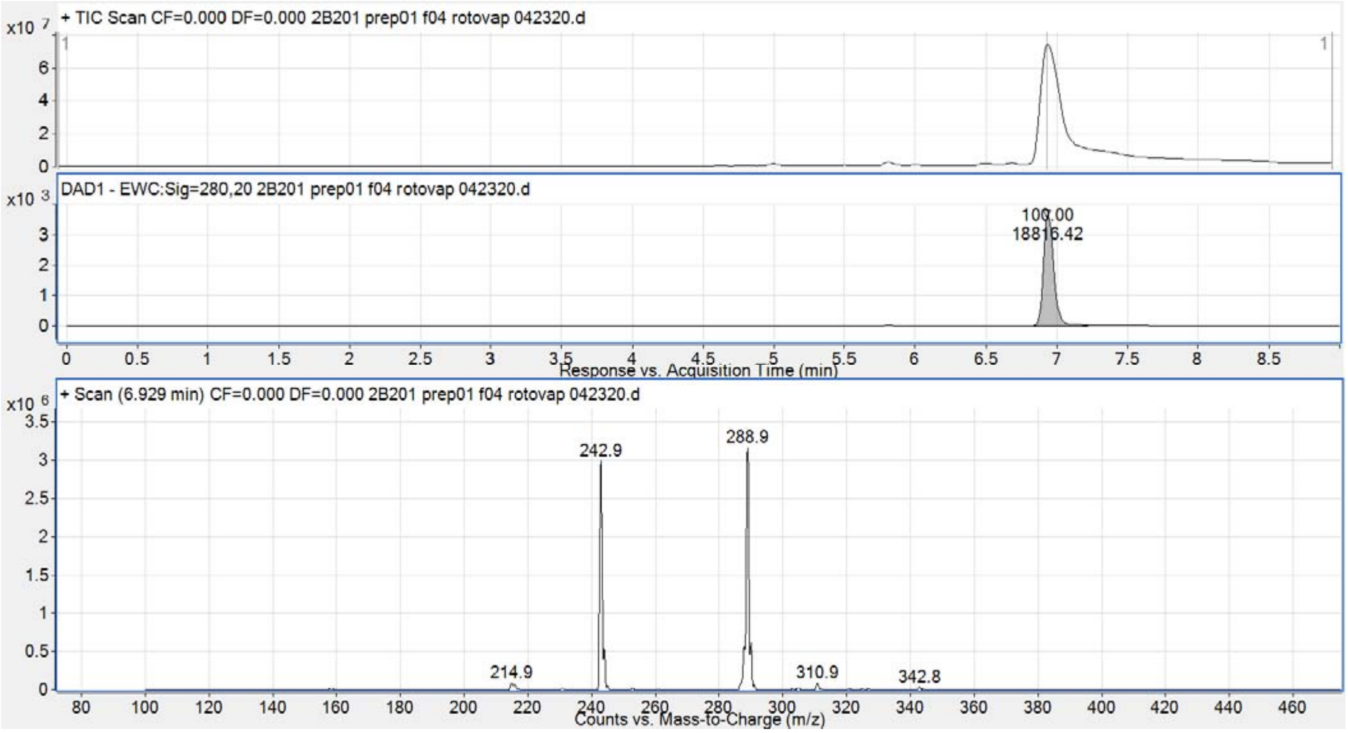

^{1}H NMR and LC-MS

Compound **4b**

4/27/2020 1:03:45 PM

Formula $C_{24}H_{25}N_3O_2S$	***FW***	419.5392

Acquisition Time (sec)	2.0486	***Comment***	Std proton	***Date***	Apr 27 2020	***Date Stamp***	Apr 27 2020
File Name	C:\Users\Mycoahhh\Documents\NMR\michan\2b198-proton-042720.fid\fid					***Frequency (MHz)***	499.83
Nucleus	1H	***Number of Transients***	24	***Original Points Count***	16415	***Points Count***	32768
Pulse Sequence	s2pul	***Receiver Gain***	36.00	***Solvent***	CHLOROFORM-d		
Spectrum Offset (Hz)	2997.5691	***Spectrum Type***	STANDARD	***Sweep Width (Hz)***	8012.82	***Temperature (degree C)***	30.000

^{1}H NMR (500 MHz, $CDCl_3$) δ 8.11 (s, 1H), 7.87 (s, 1H), 7.71 (br. s., 1H), 7.42 - 7.48 (m, 3H), 7.32 - 7.41 (m, 3H), 7.25 (t, J = 7.30 Hz, 1H), 4.41 (q, J = 7.09 Hz, 2H), 4.05 (s, 3H), 2.40 (t, J = 6.97 Hz, 2H), 1.65 (sxt, J = 7.24 Hz, 2H), 1.40 (t, J = 6.97 Hz, 3H), 1.07 (t, J = 7.34 Hz, 3H)

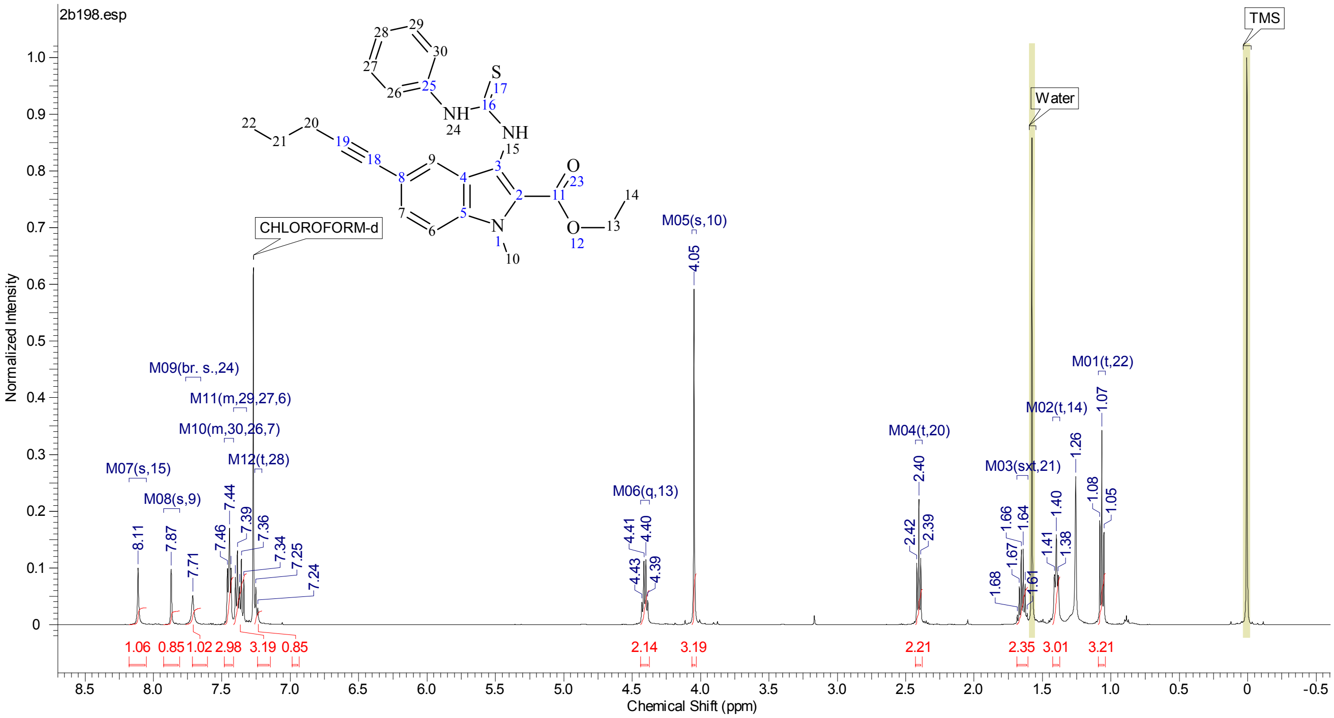

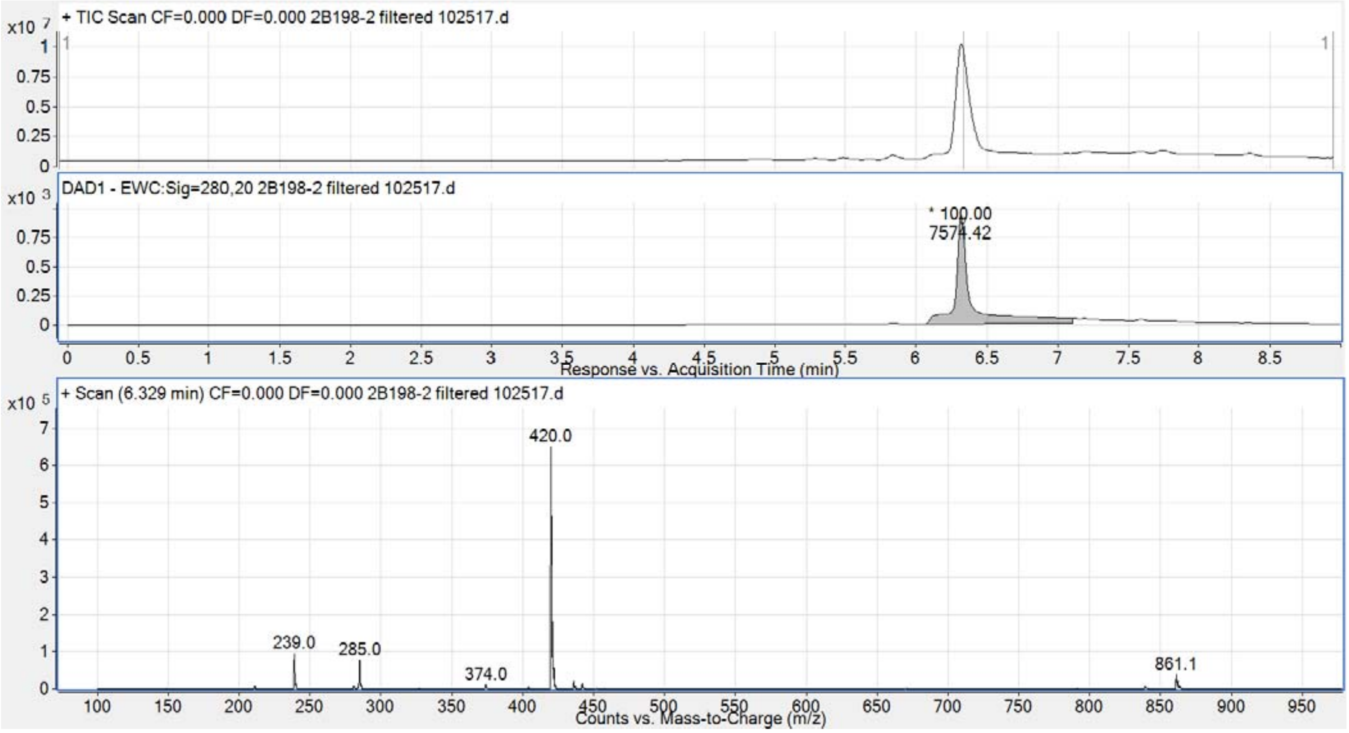

^{1}H and ^{13}C NMR
Compound **6b**

5/4/2020 9:59:36 AM

Formula $C_{30}H_{32}N_4O_2S$	*FW*	512.6657

Acquisition Time (sec)	2.0486	*Comment*	Std proton	*Date*	Apr 29 2020	*Date Stamp*	Apr 29 2020
File Name	C:\Users\Mycoahhh\Documents\NMR\michan\2B204-proton.fid\fid					*Frequency (MHz)*	499.83
Nucleus	1H	*Number of Transients*	16	*Original Points Count*	16415	*Points Count*	32768
Pulse Sequence	s2pul	*Receiver Gain*	34.00	*Solvent*	CHLOROFORM-d		
Spectrum Offset (Hz)	2998.0586	*Spectrum Type*	STANDARD	*Sweep Width (Hz)*	8012.82	*Temperature (degree C)*	30.000

^{1}H NMR (500 MHz, $CDCl_3$) δ 8.17 (s, 1H), 7.56 - 7.62 (m, 4H), 7.40 (d, J = 8.56 Hz, 2H), 7.32 - 7.38 (m, 2H), 4.19 (s, 3H), 3.73 - 3.81 (m, 3H), 2.45 (t, J = 6.97 Hz, 2H), 1.89 (s, 2H), 1.68 (sxt, J = 7.30 Hz, 2H), 1.50 - 1.55 (m, 1H), 1.26 - 1.39 (m, 4H), 1.08 - 1.18 (m, 6H)

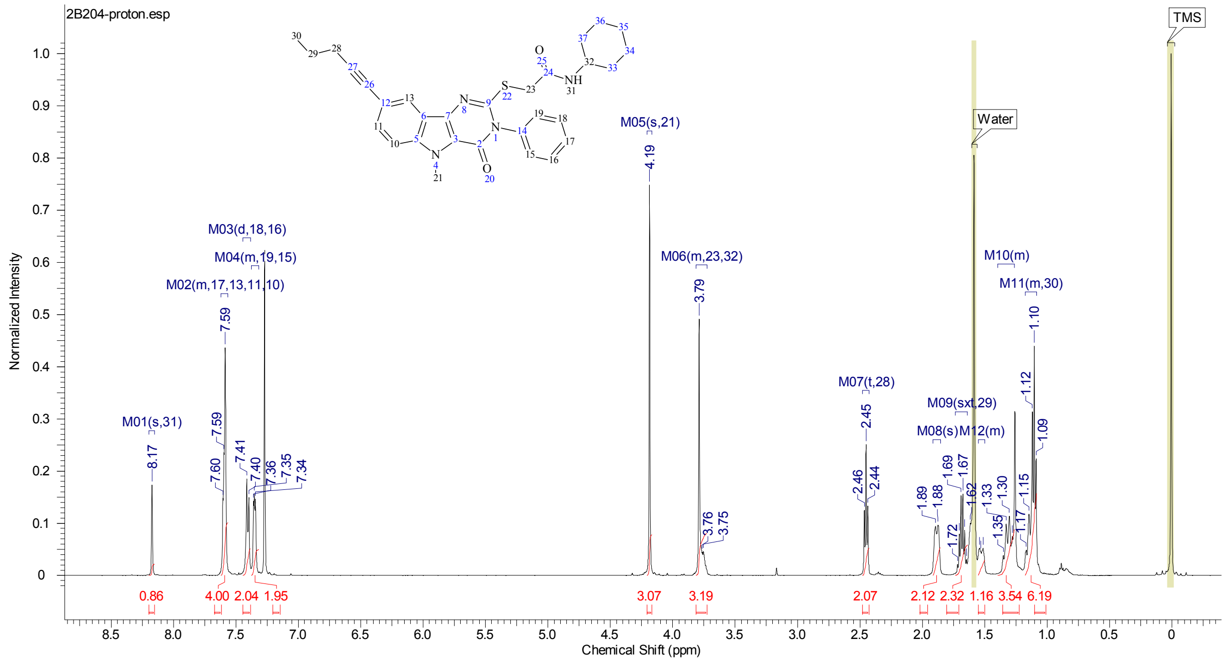

Acquisition Time (sec)	1.3005	*Comment*	Std carbon	*Date*	Apr 29 2020	*Date Stamp*	Apr 29 2020
File Name	C:\Users\nikunjshukla\Desktop\BAA1 Pyrimidoindole TLR4 Agonist\2B204-carbon.fid\fid					*Frequency (MHz)*	125.69
Nucleus	13C	*Number of Transients*	132	*Original Points Count*	39649	*Points Count*	65536
Pulse Sequence	s2pul	*Receiver Gain*	30.00	*Solvent*	CHLOROFORM-d		
Spectrum Offset (Hz)	13192.8906	*Spectrum Type*	STANDARD	*Sweep Width (Hz)*	30487.80	*Temperature (degree C)*	30.000

^{13}C NMR (126 MHz, $CDCl_3$) δ 167.7, 156.0, 153.8, 139.5, 137.1, 135.4, 131.3, 130.3, 129.9, 129.1, 124.0, 119.9, 119.7, 116.5, 110.2, 89.2, 80.7, 36.2, 32.8, 31.3, 29.7, 25.4, 24.6, 22.3, 21.4, 13.6

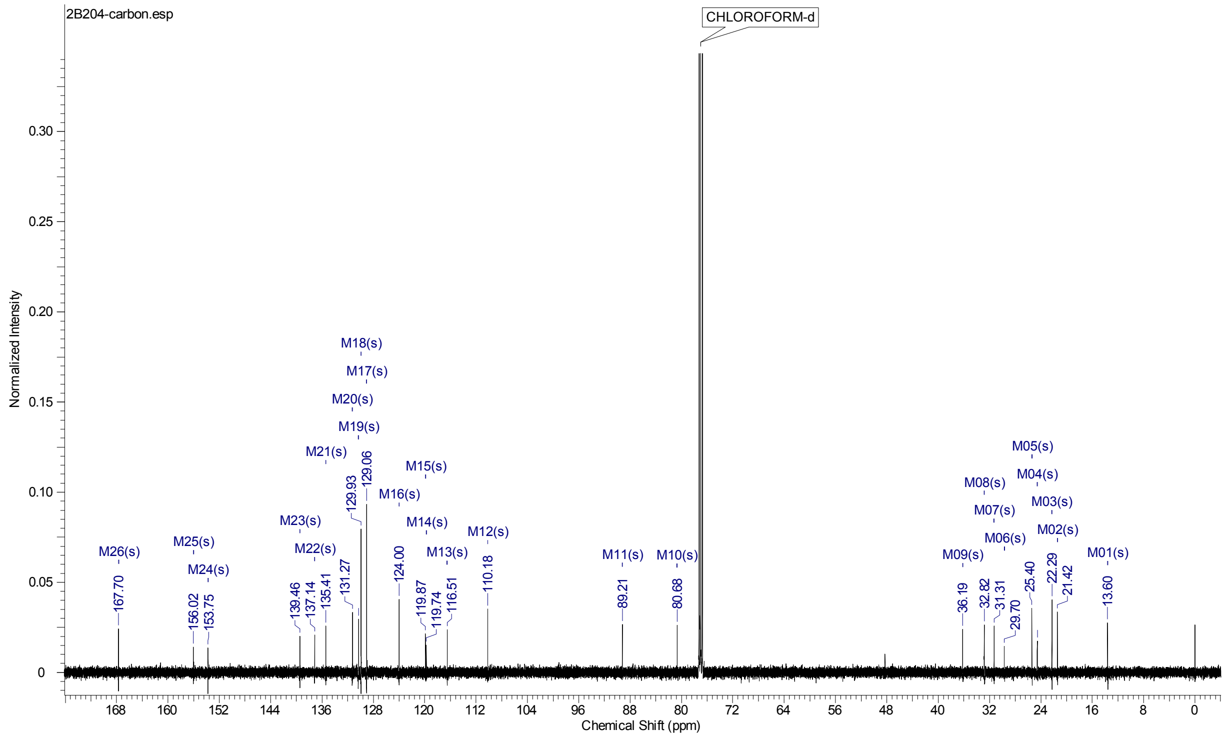

LC-MS

Compound **6a**

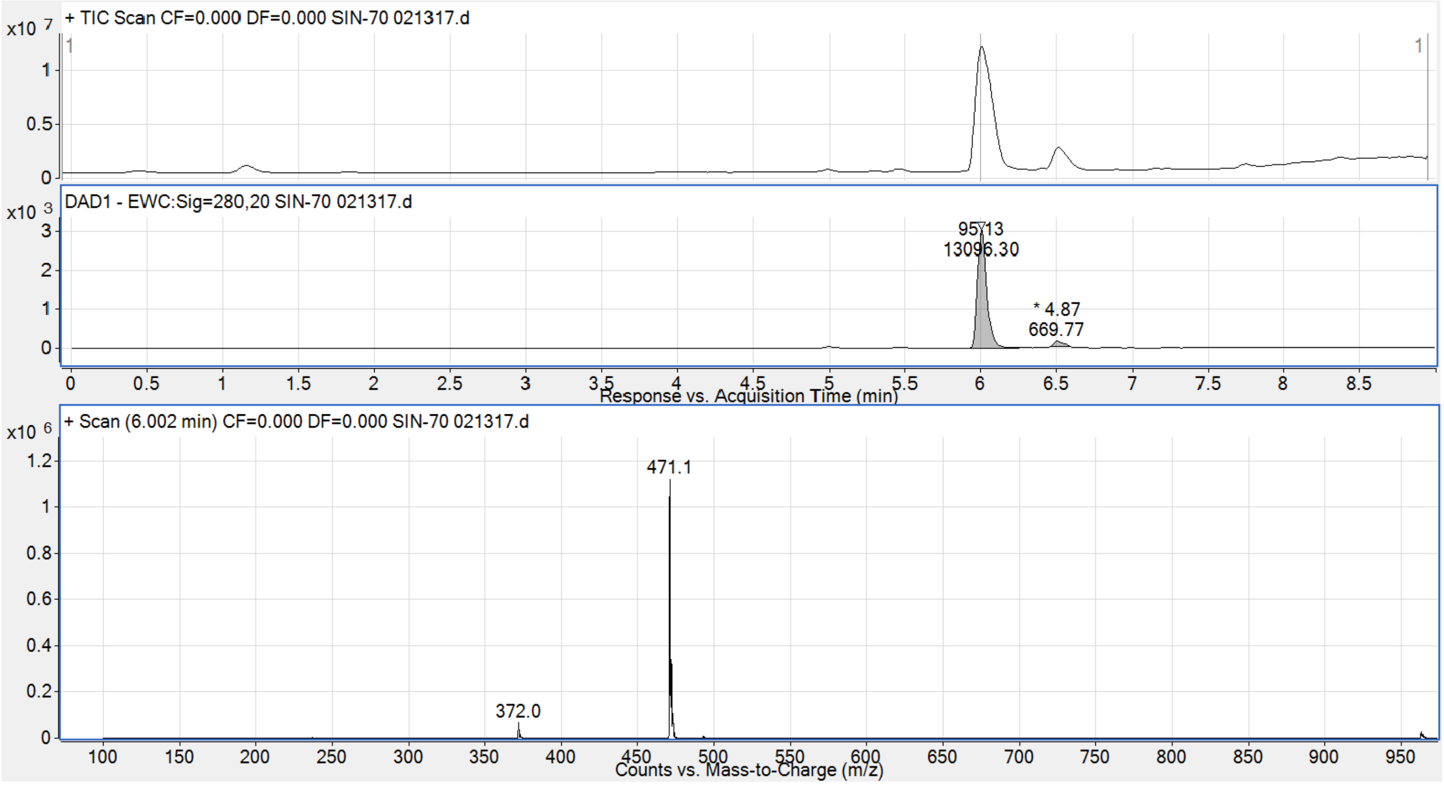

Compound **6b**

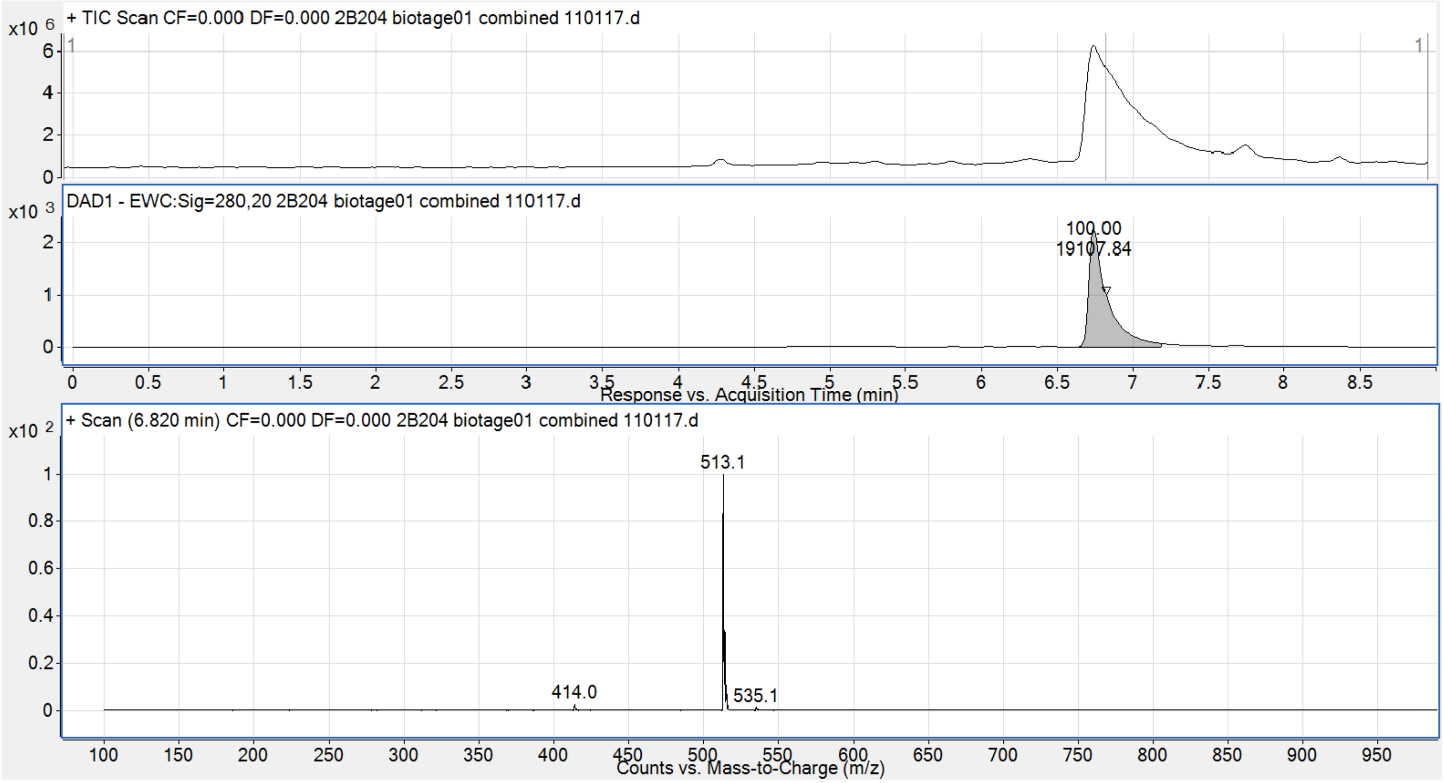

LC-MS

Compound **6c**

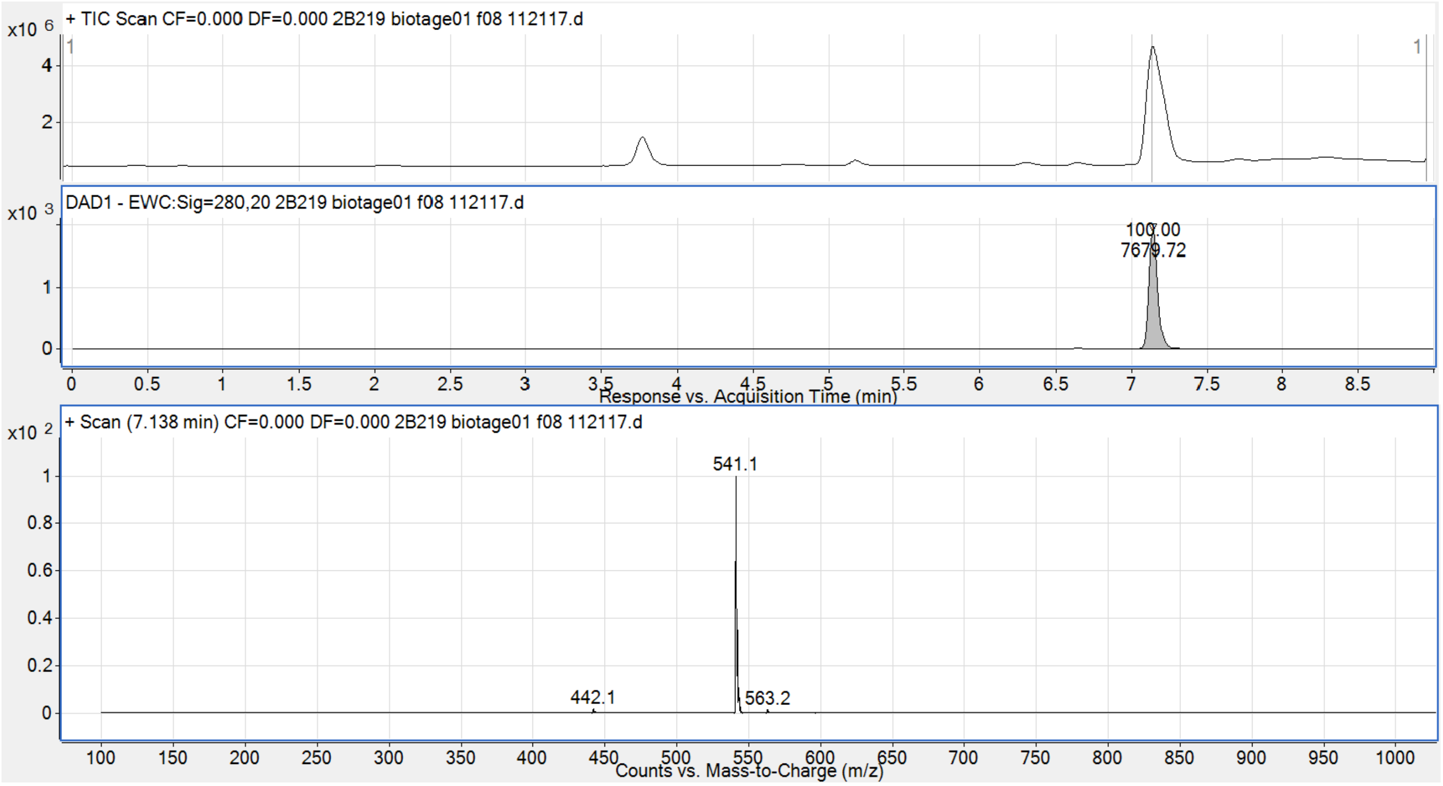

Compound **6d**

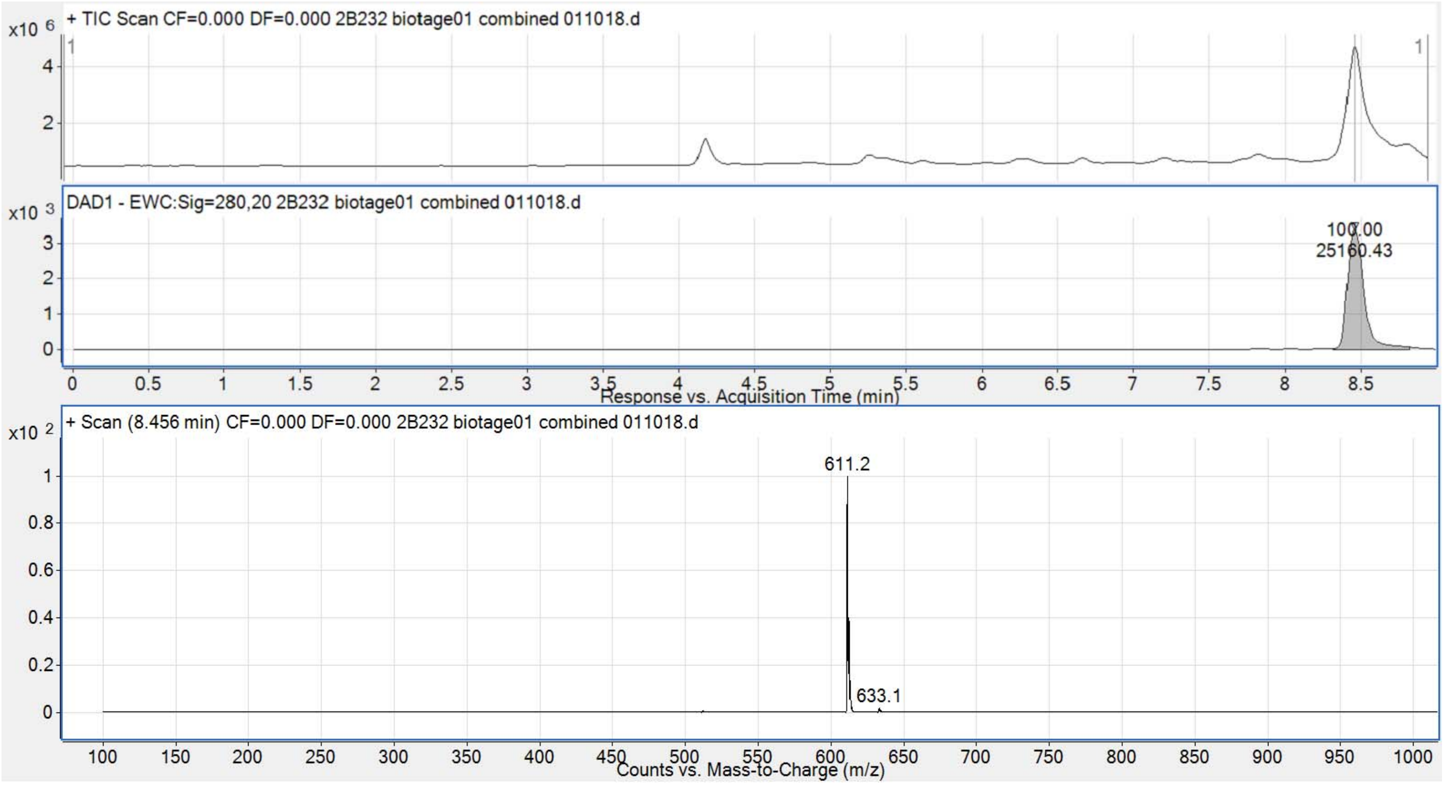

LC-MS

Compound **6e**

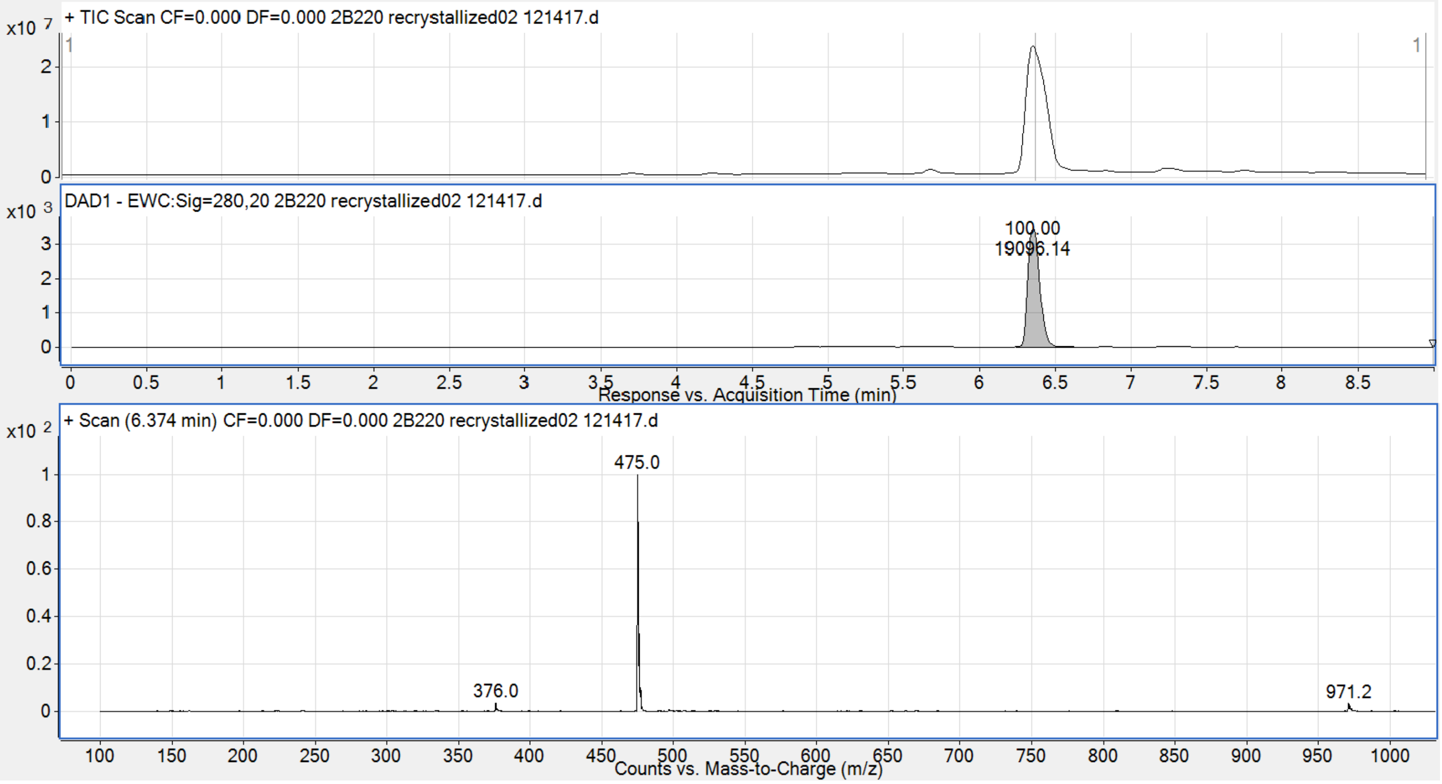

Compound **6f**

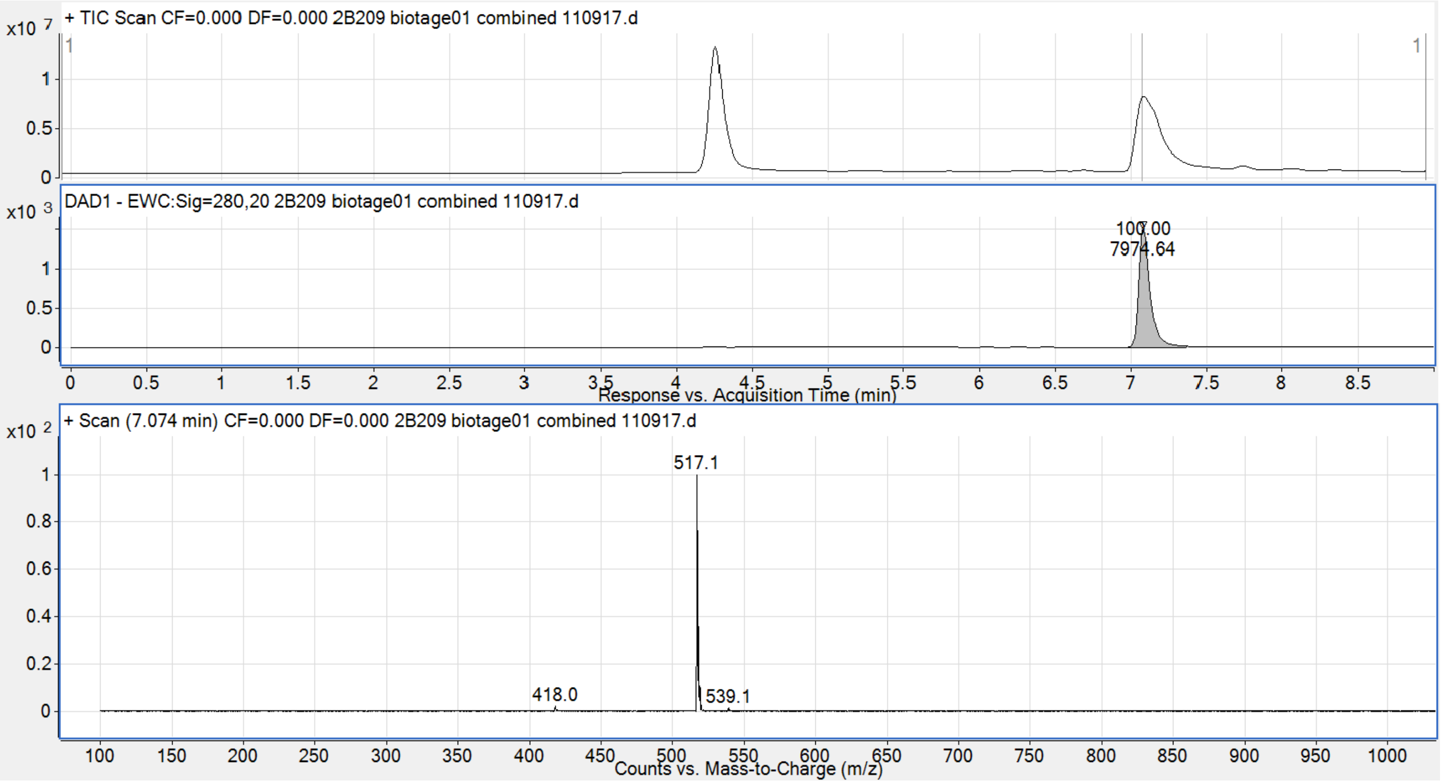

LC-MS

Compound **6g**

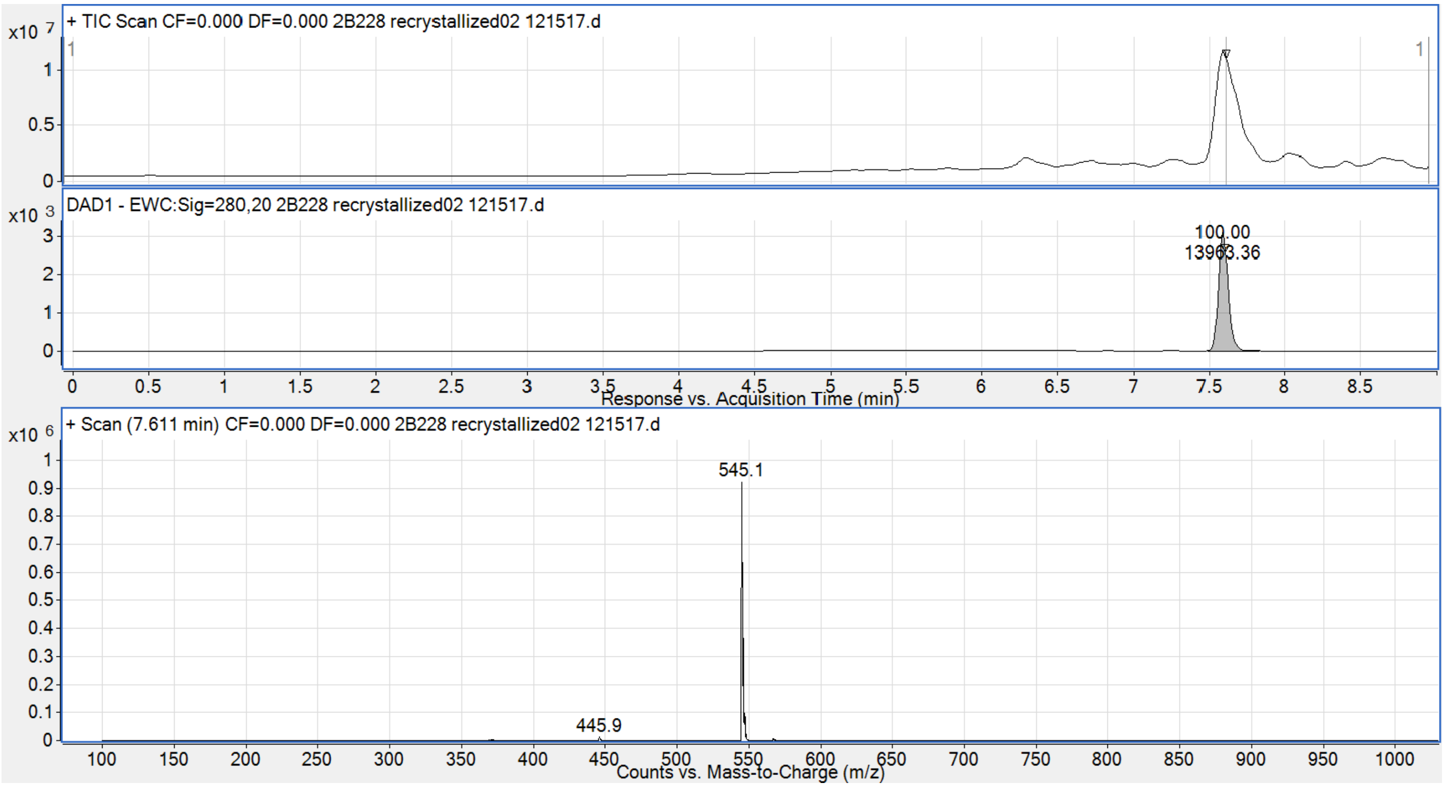

Compound **8a**

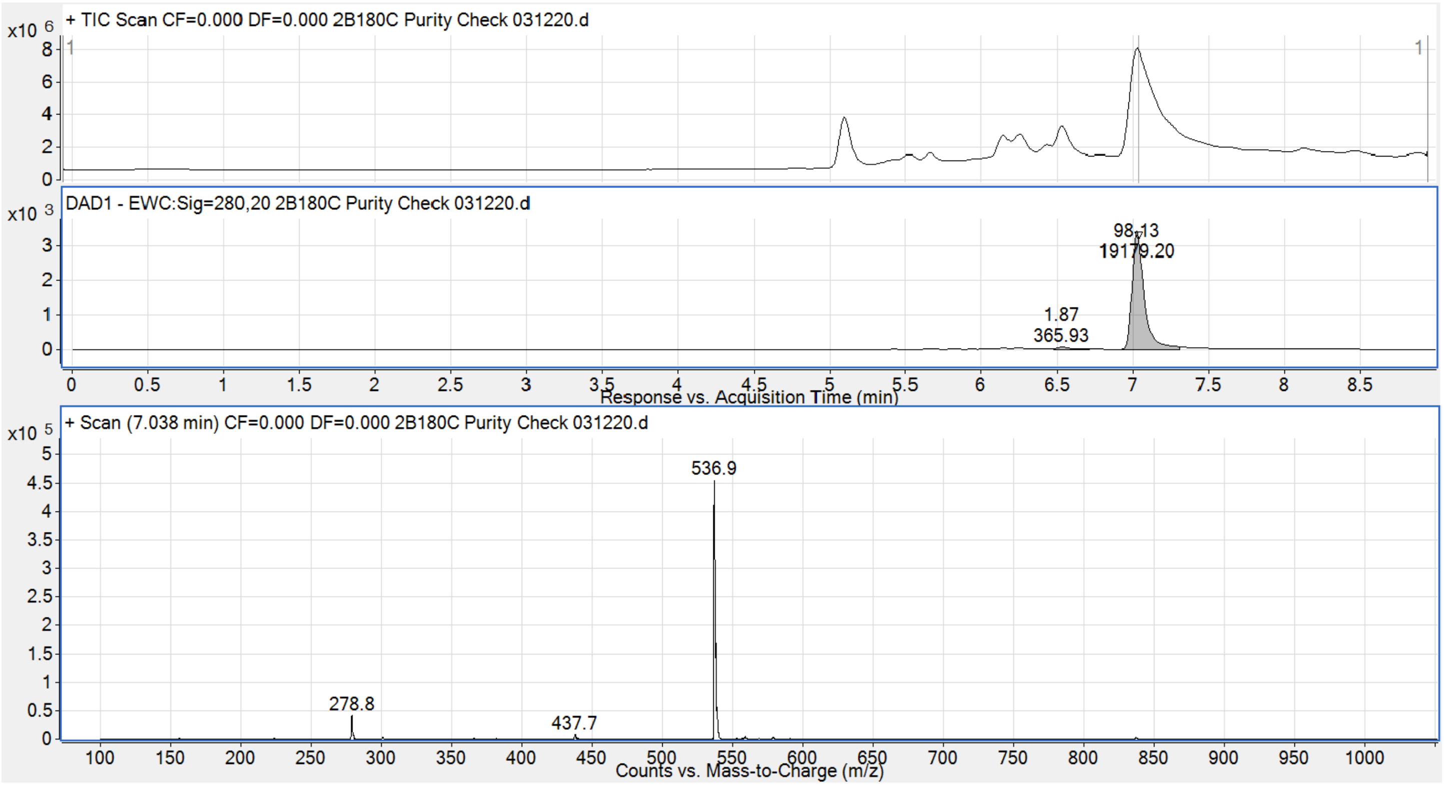

LC-MS

Compound **8b**

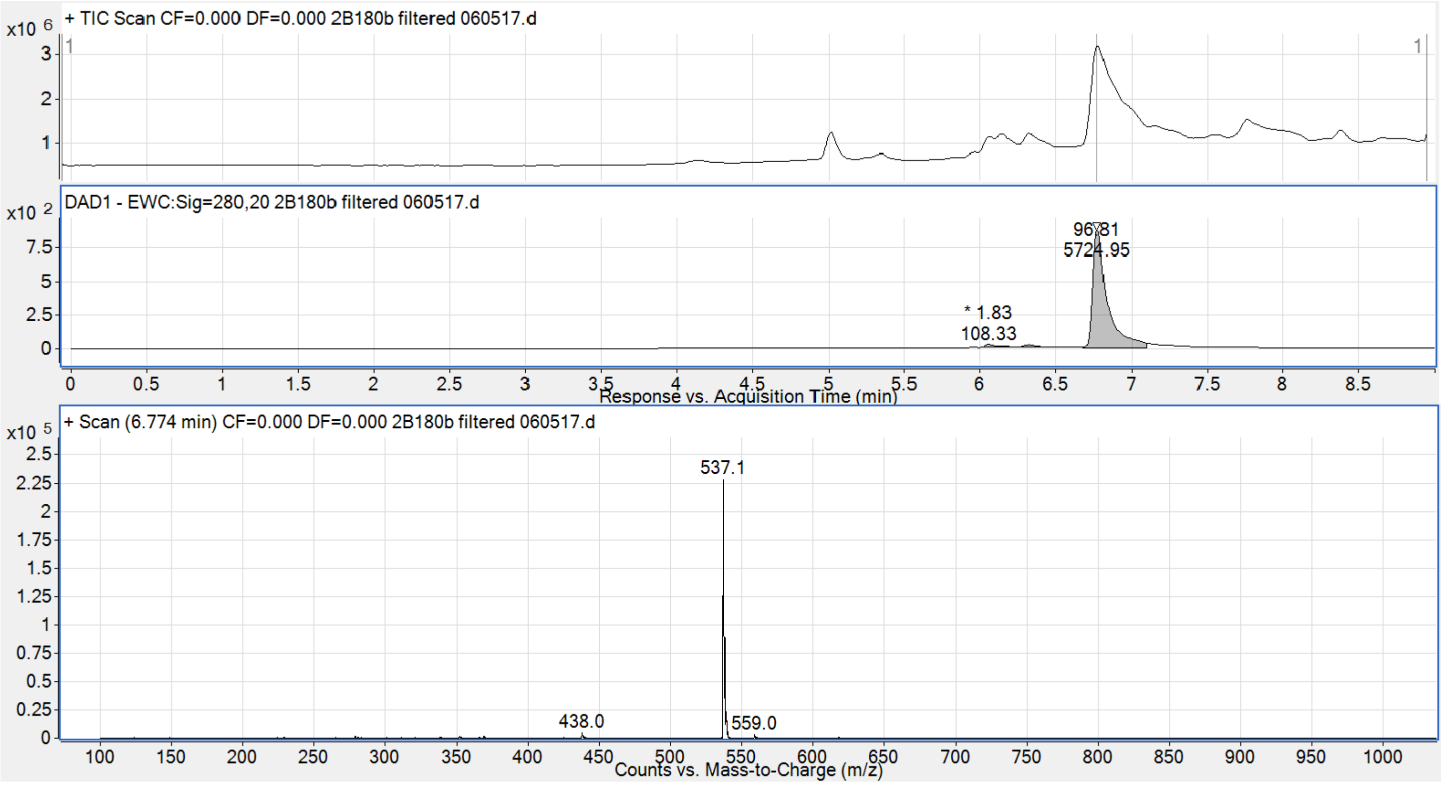

Compound **8c**

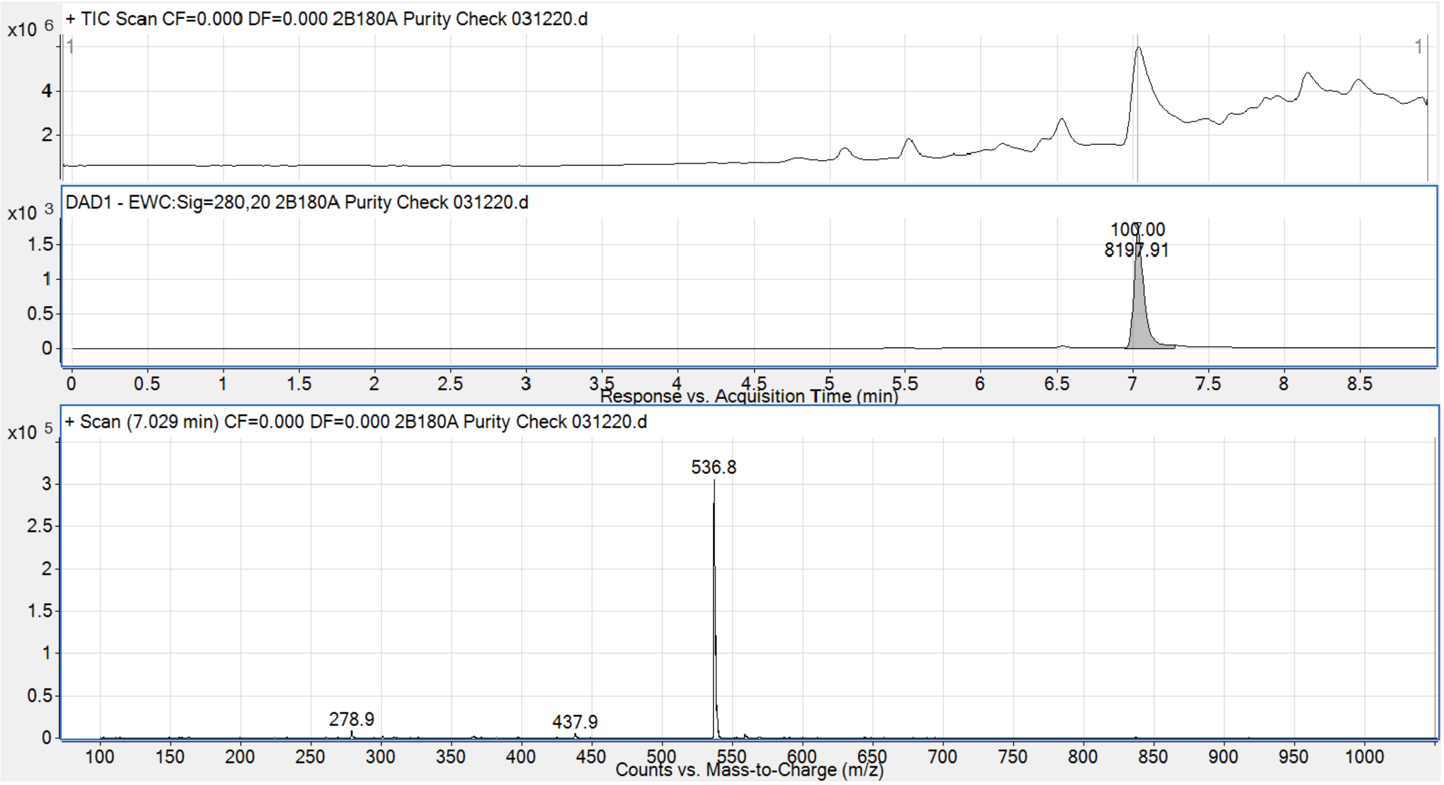

LC-MS

Compound **8d**

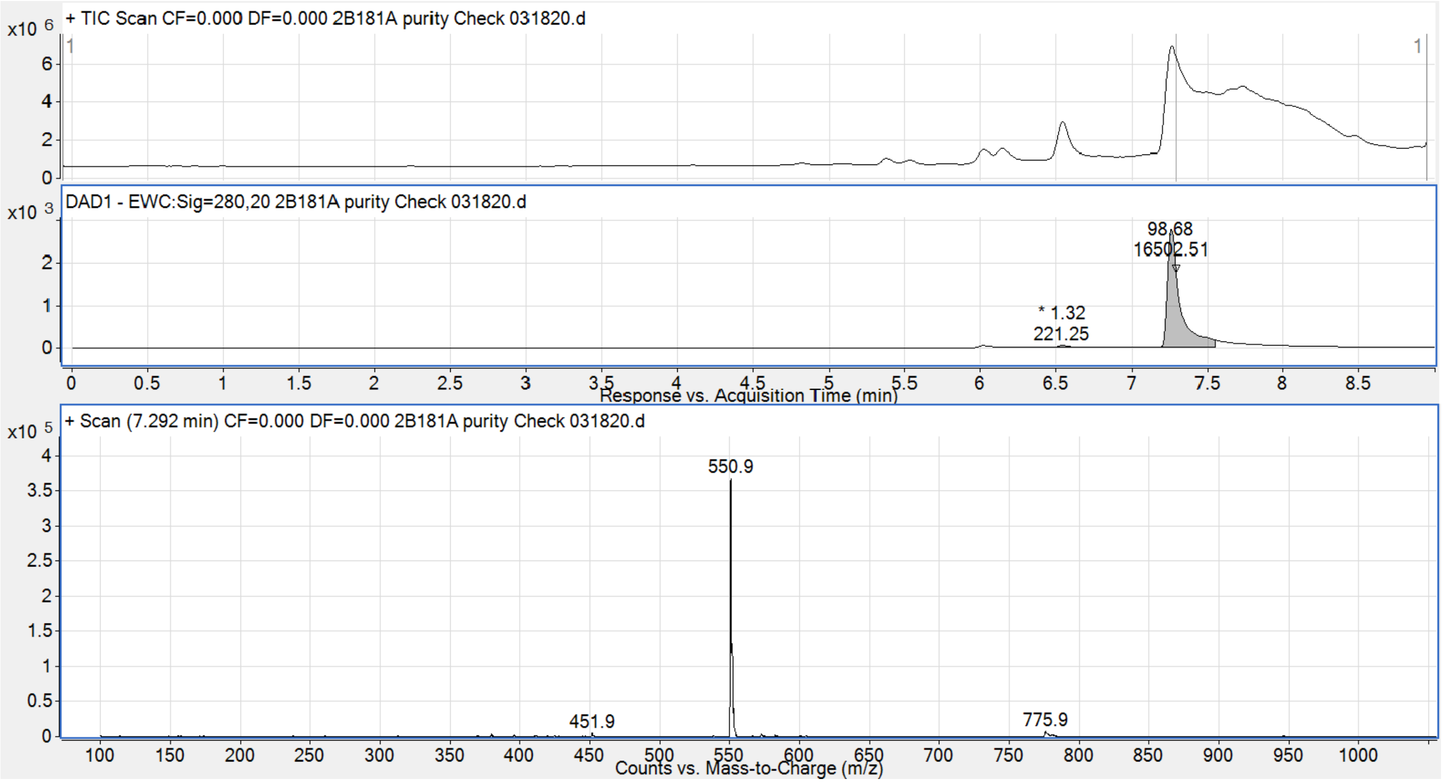

Compound **8e**

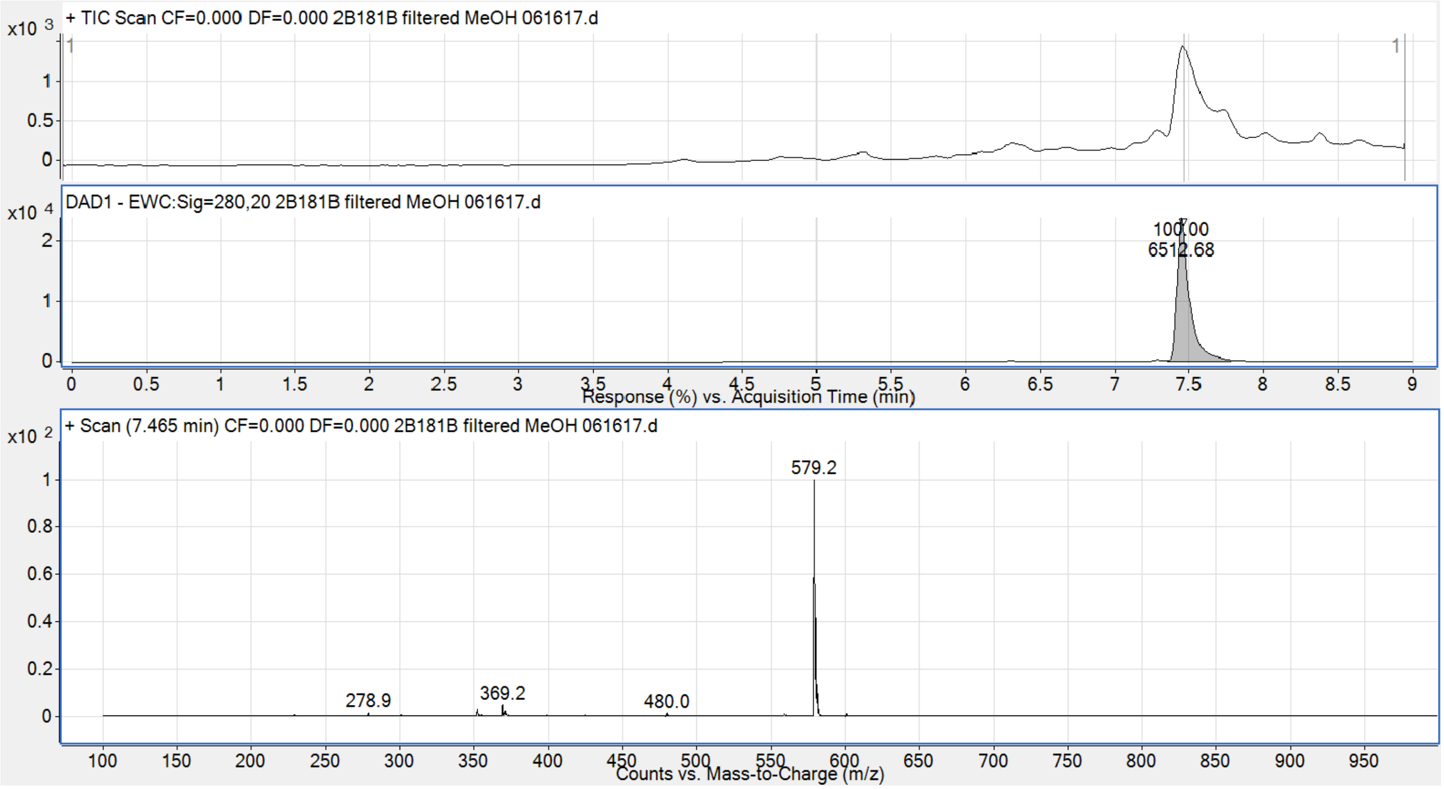

LC-MS

Compound **8f**

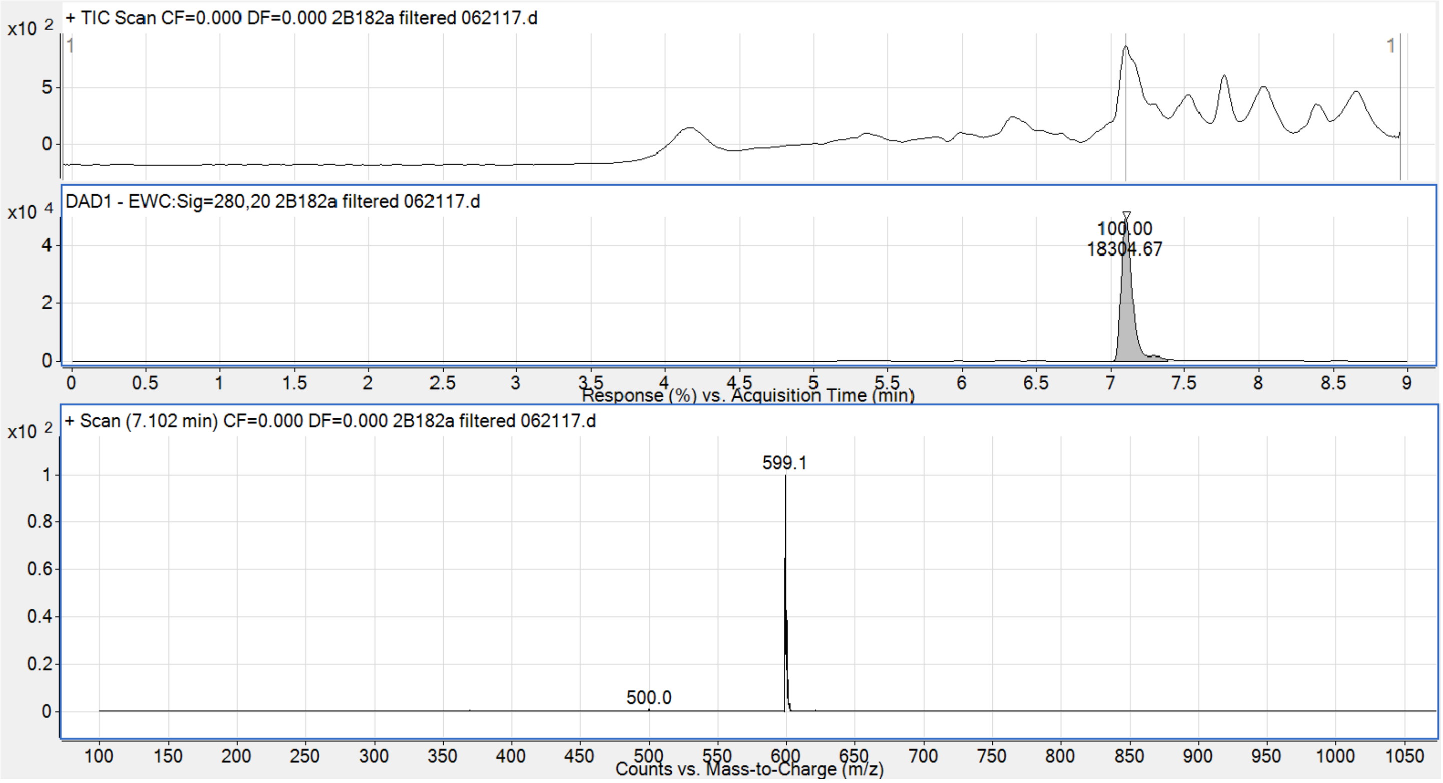

Compound **8g**

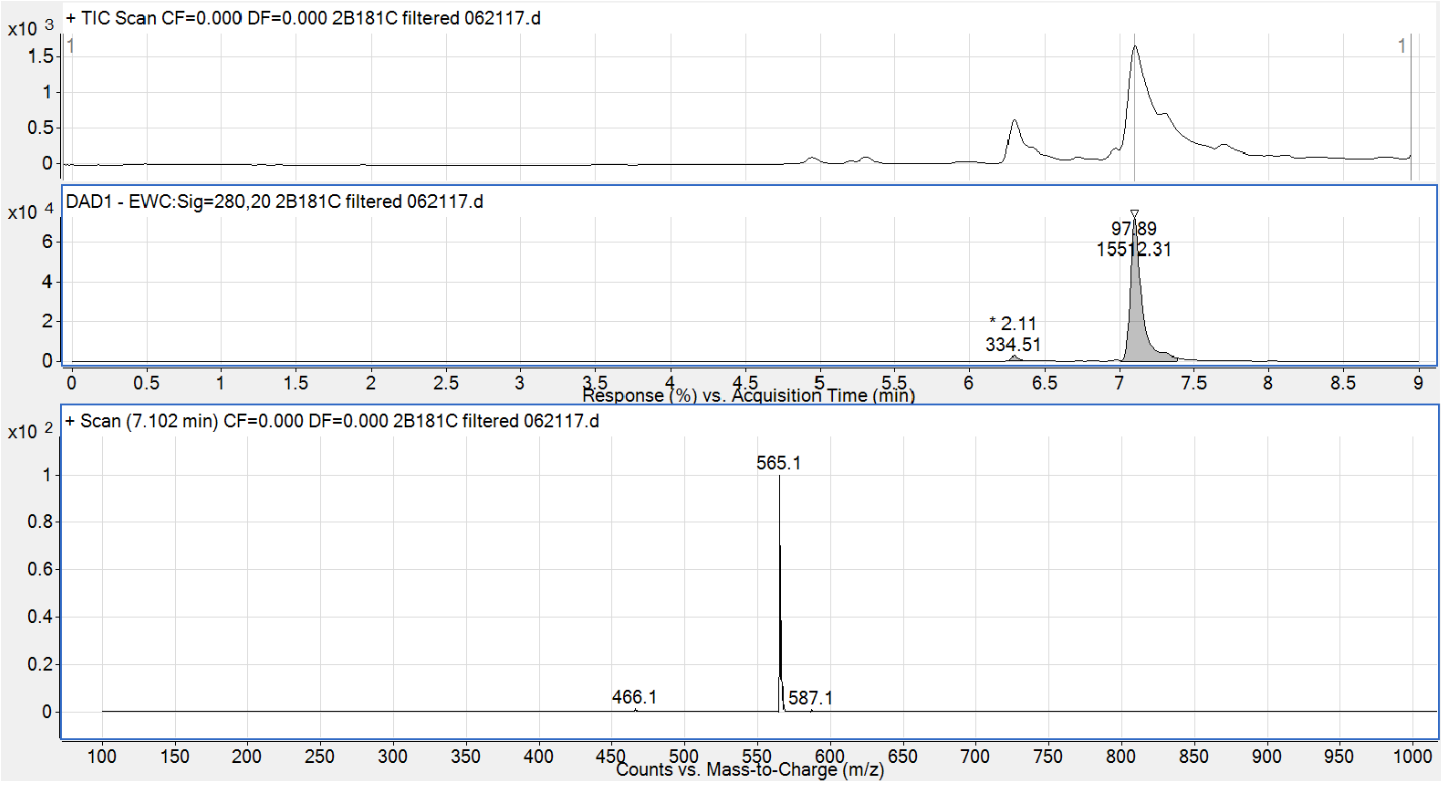

LC-MS

Compound **8h**

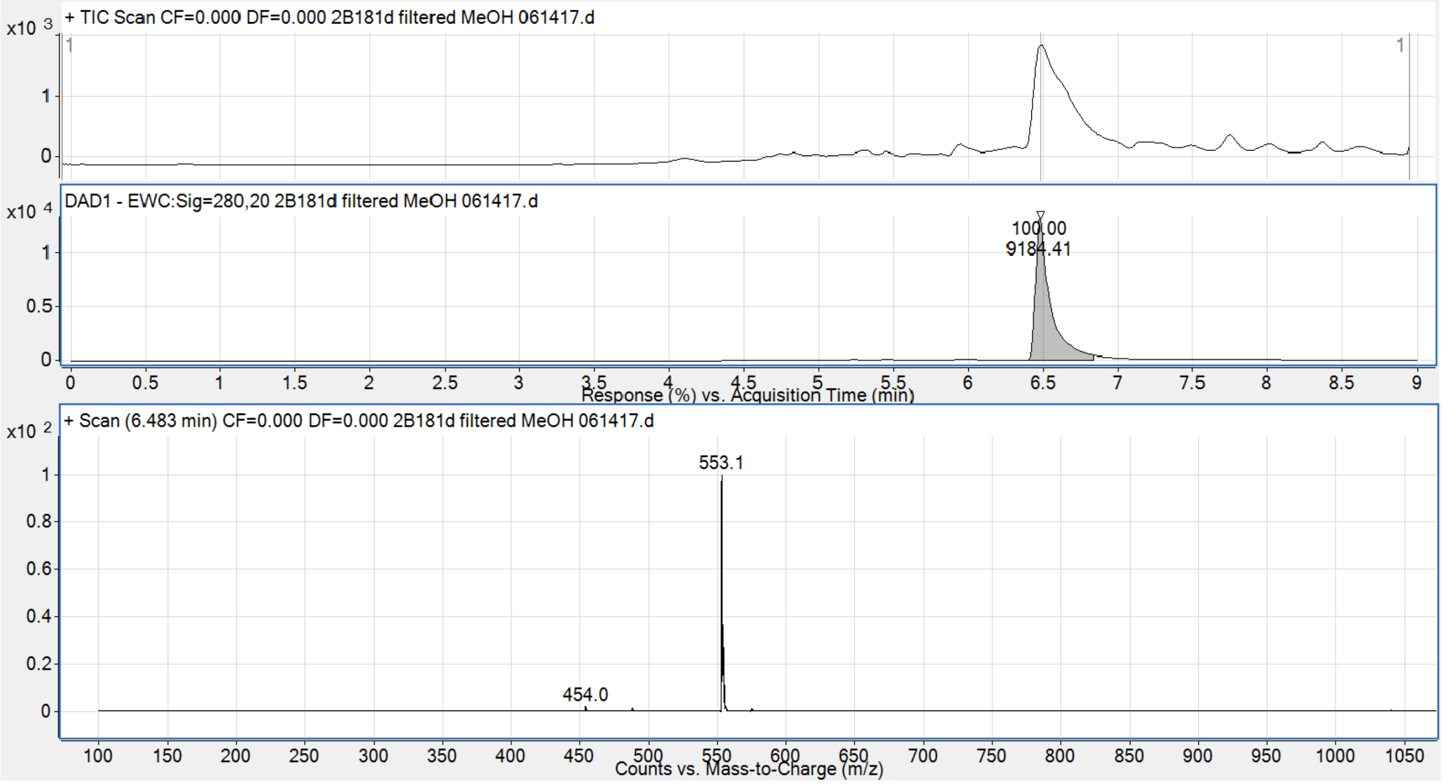

Compound **8i**

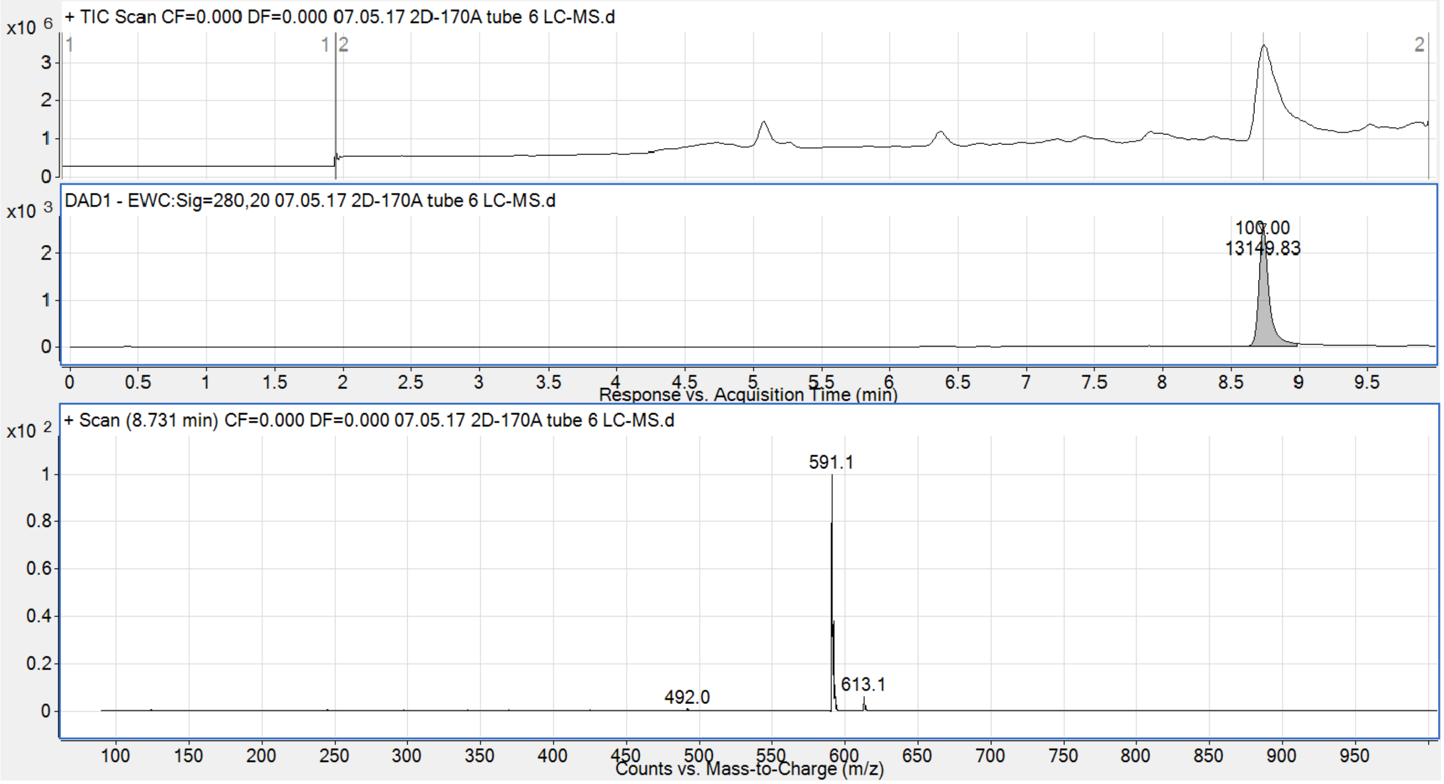

LC-MS

Compound **8j**

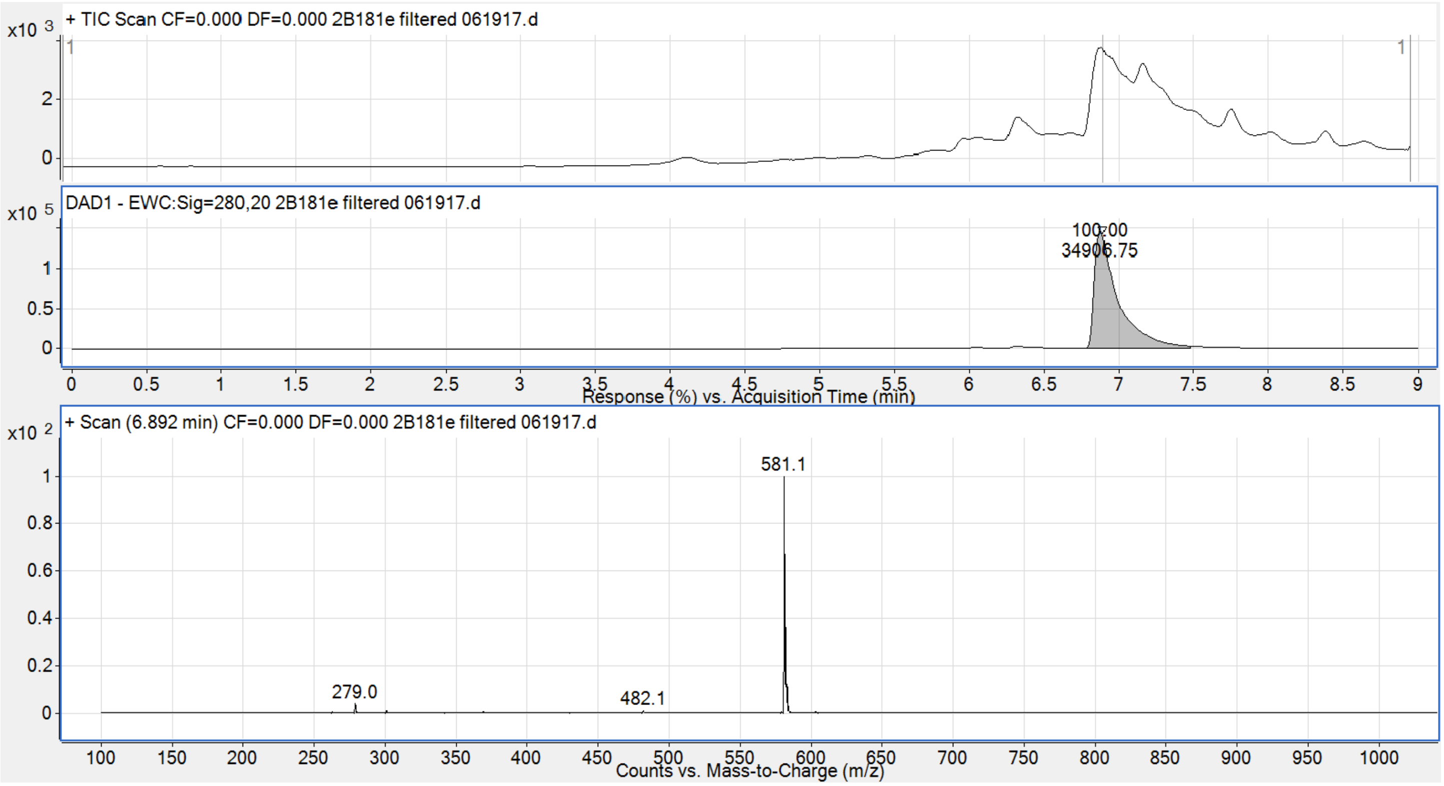

Compound **8k**

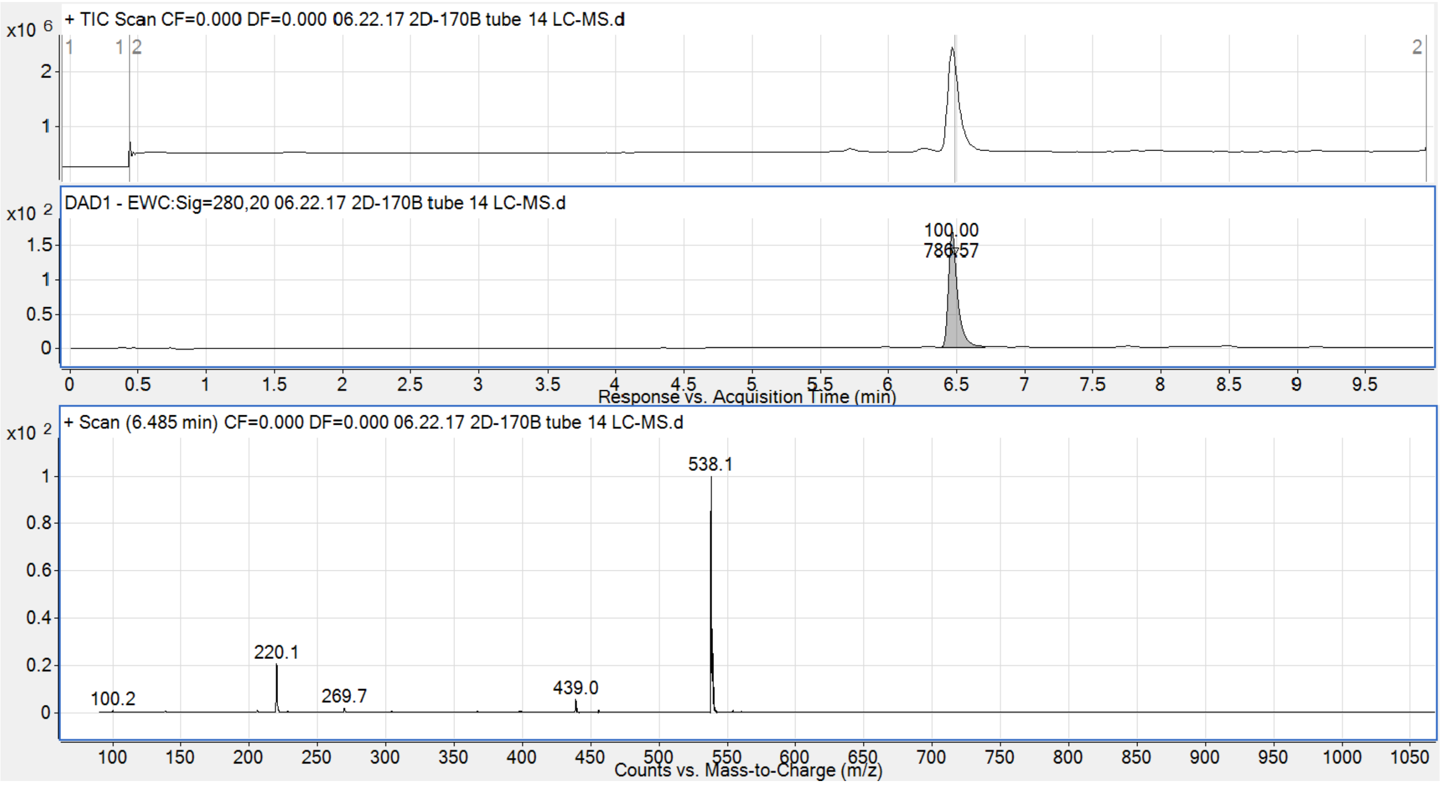

LC-MS

Compound **8l**

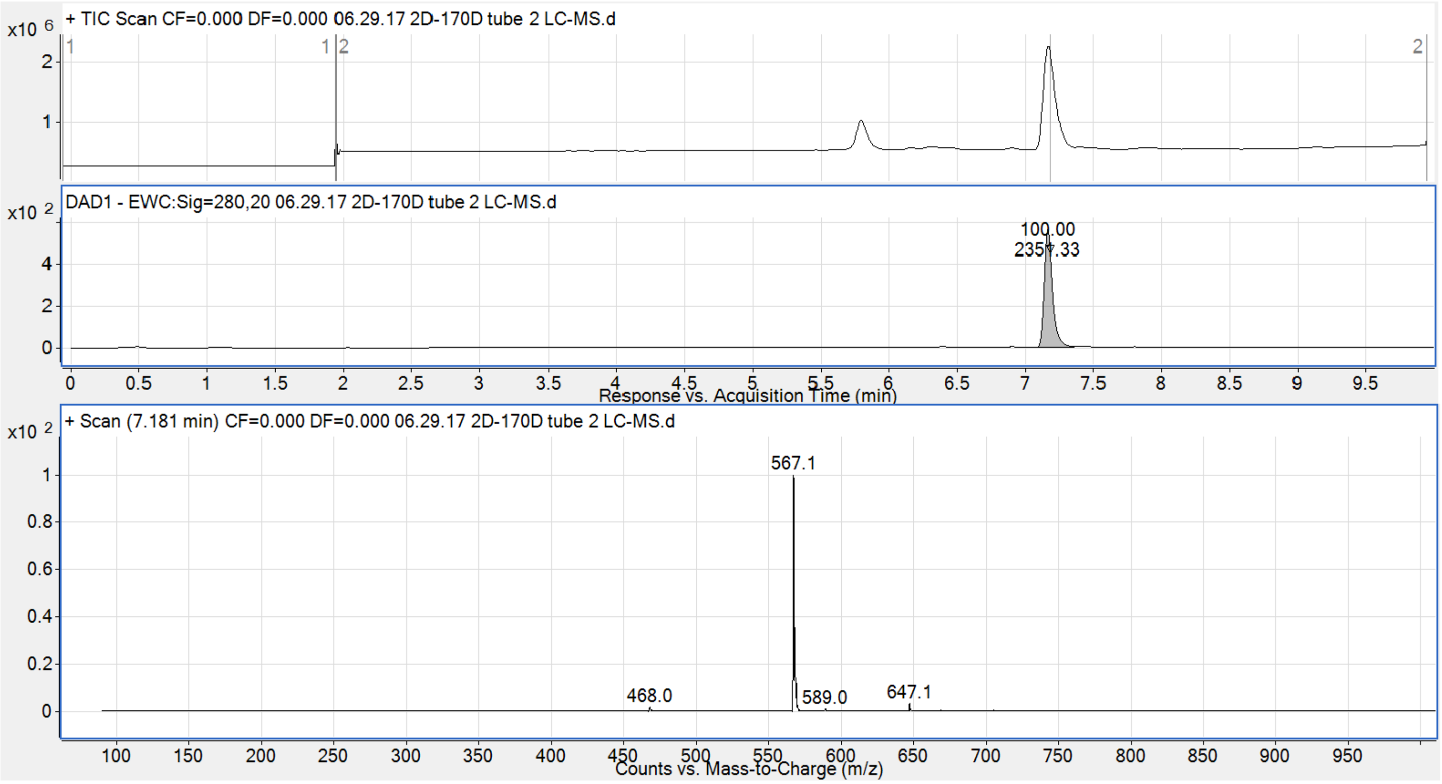

Compound **8m**

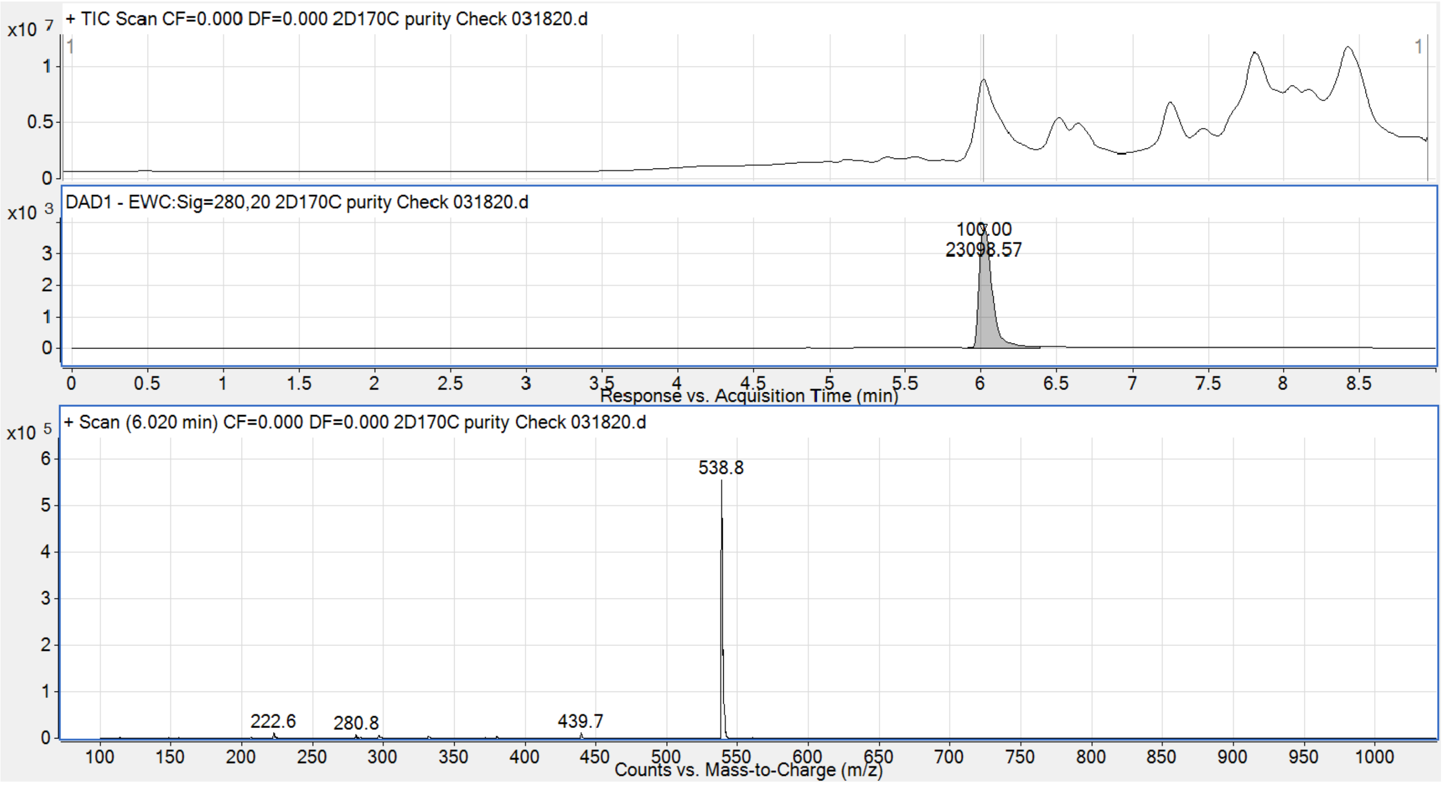

LC-MS

Compound **8n**

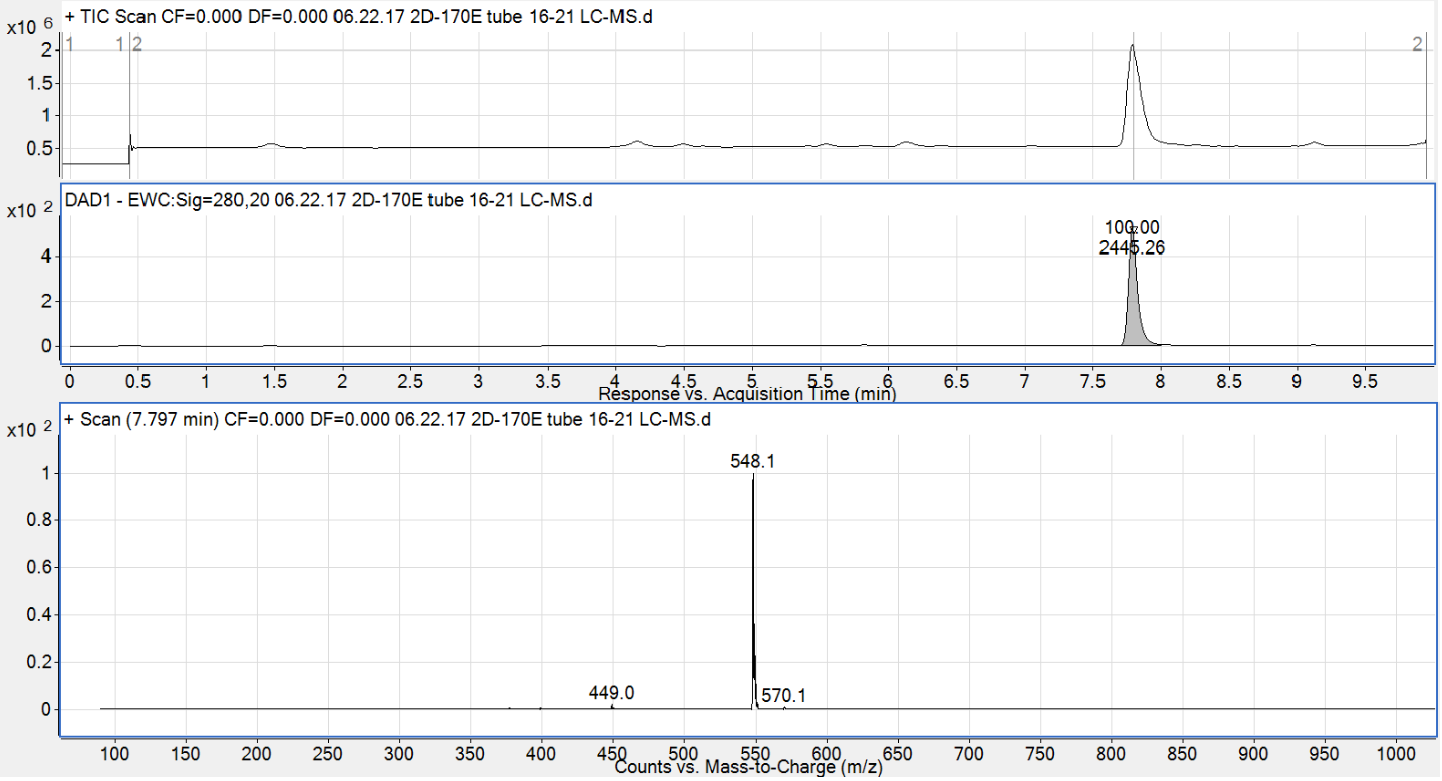

Compound **8o**

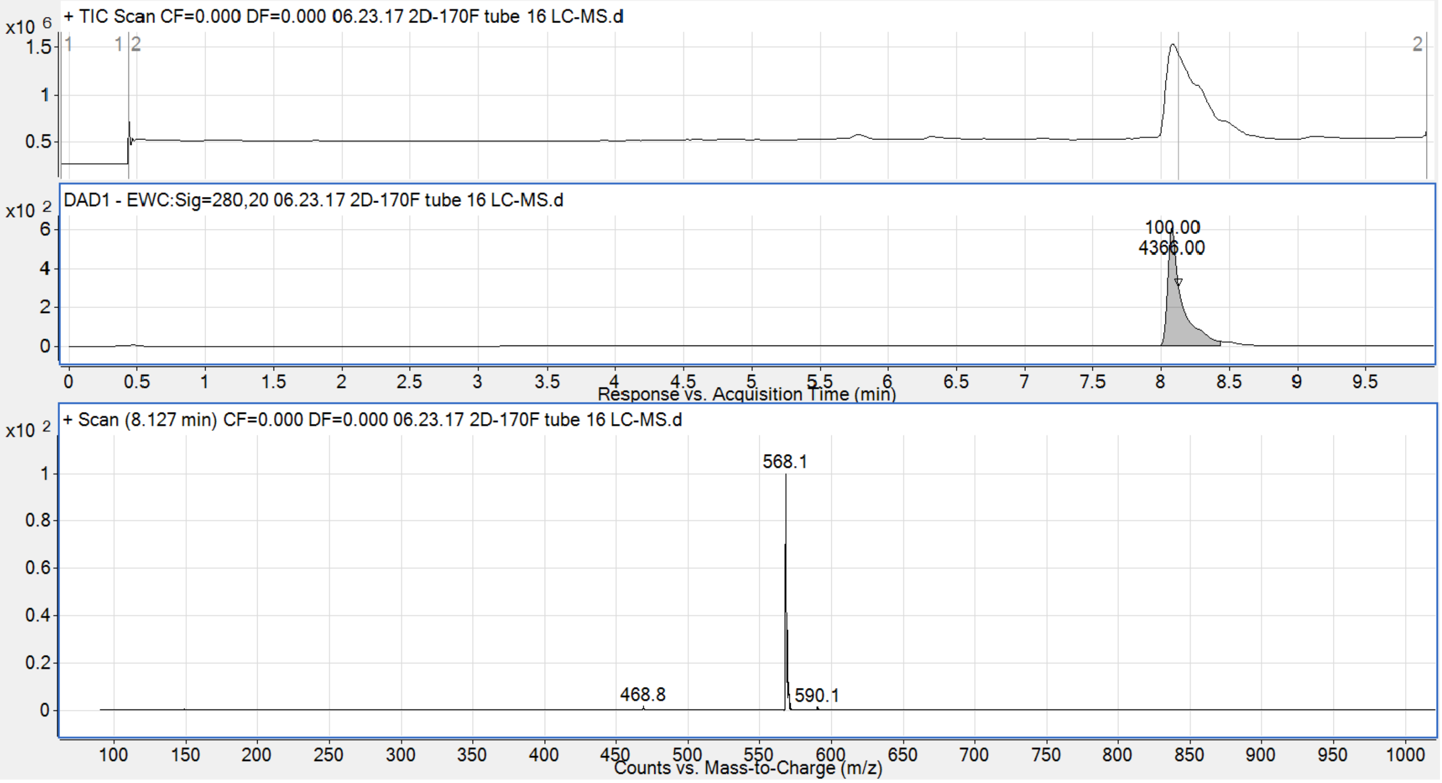

LC-MS

Compound **8p**

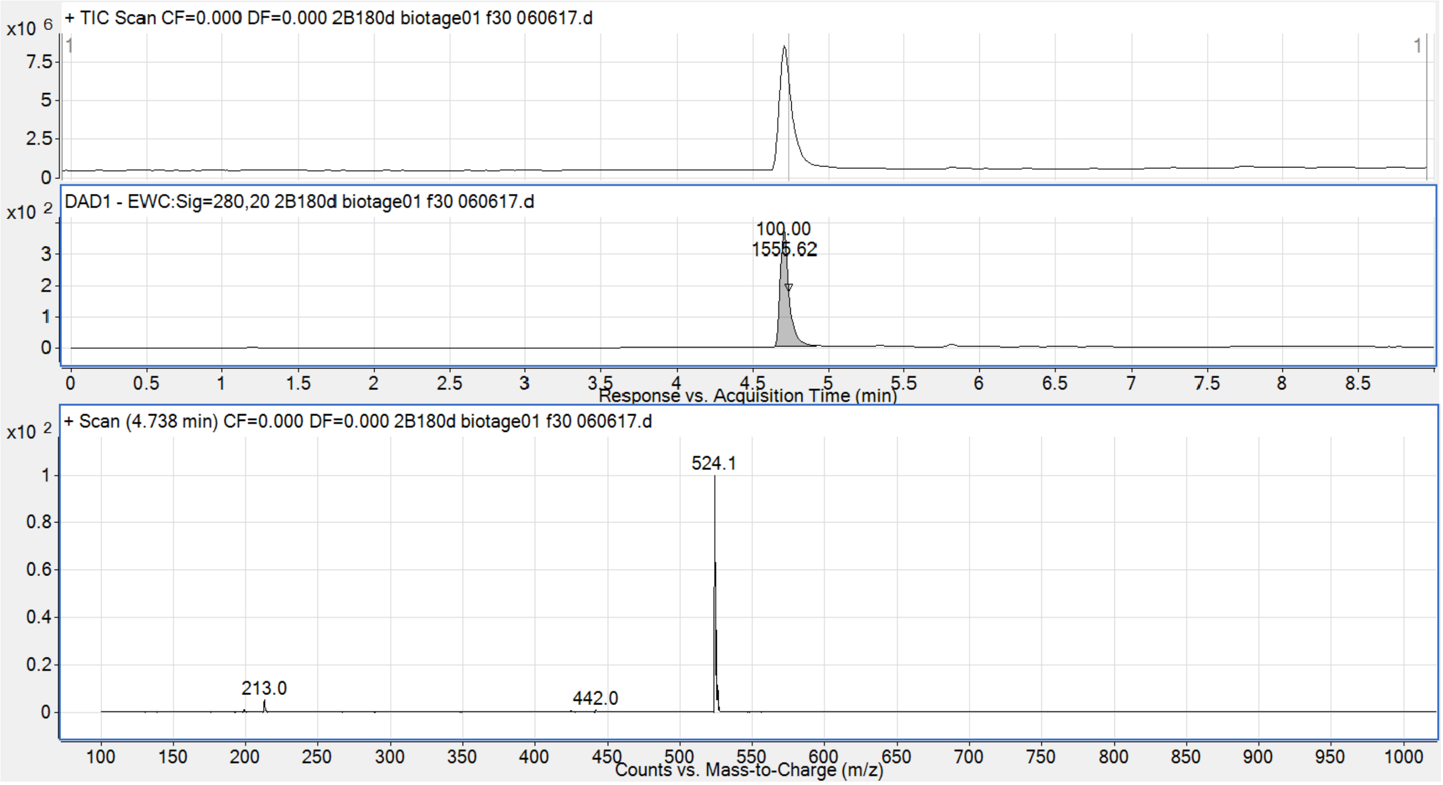

Compound **8q**

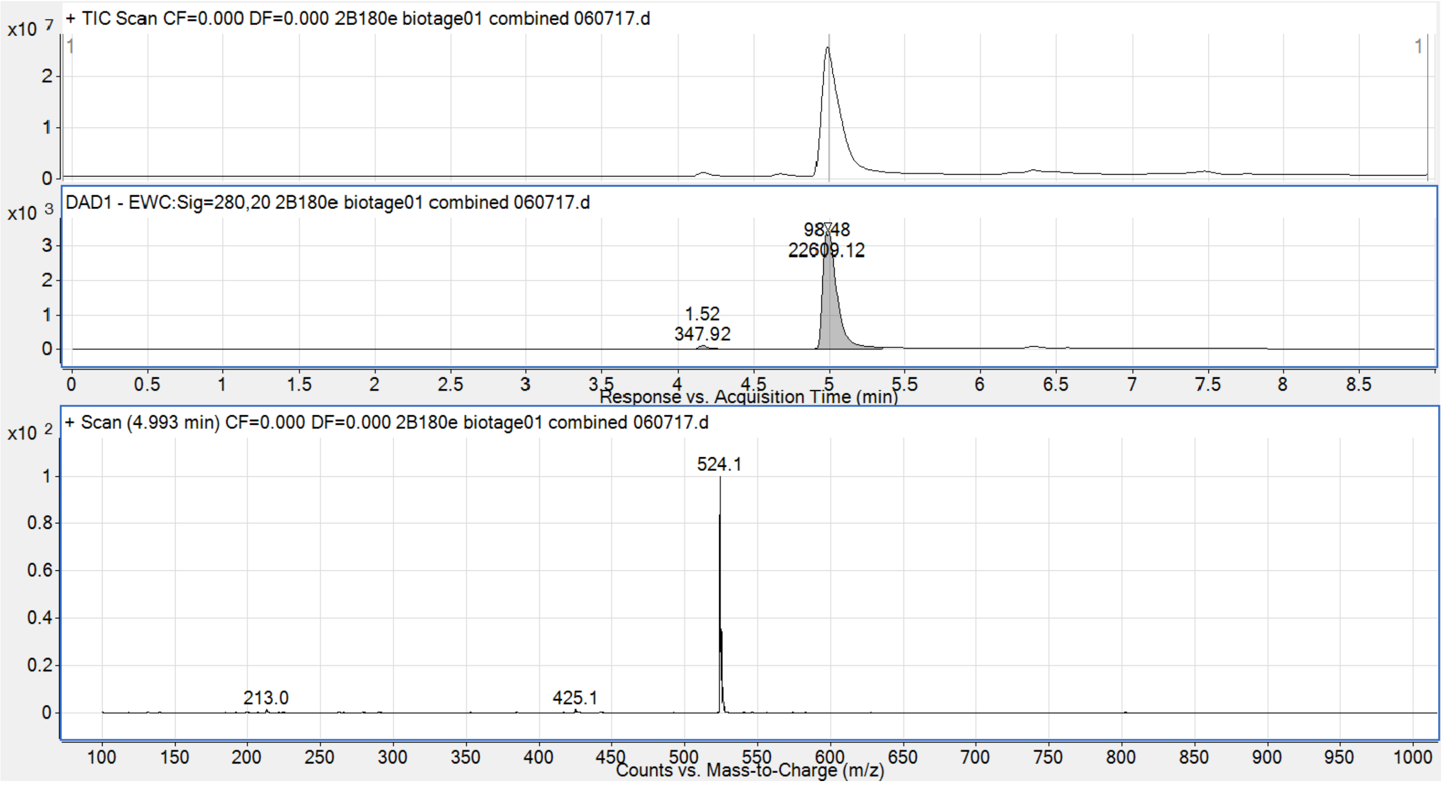

LC-MS

Compound **8r**

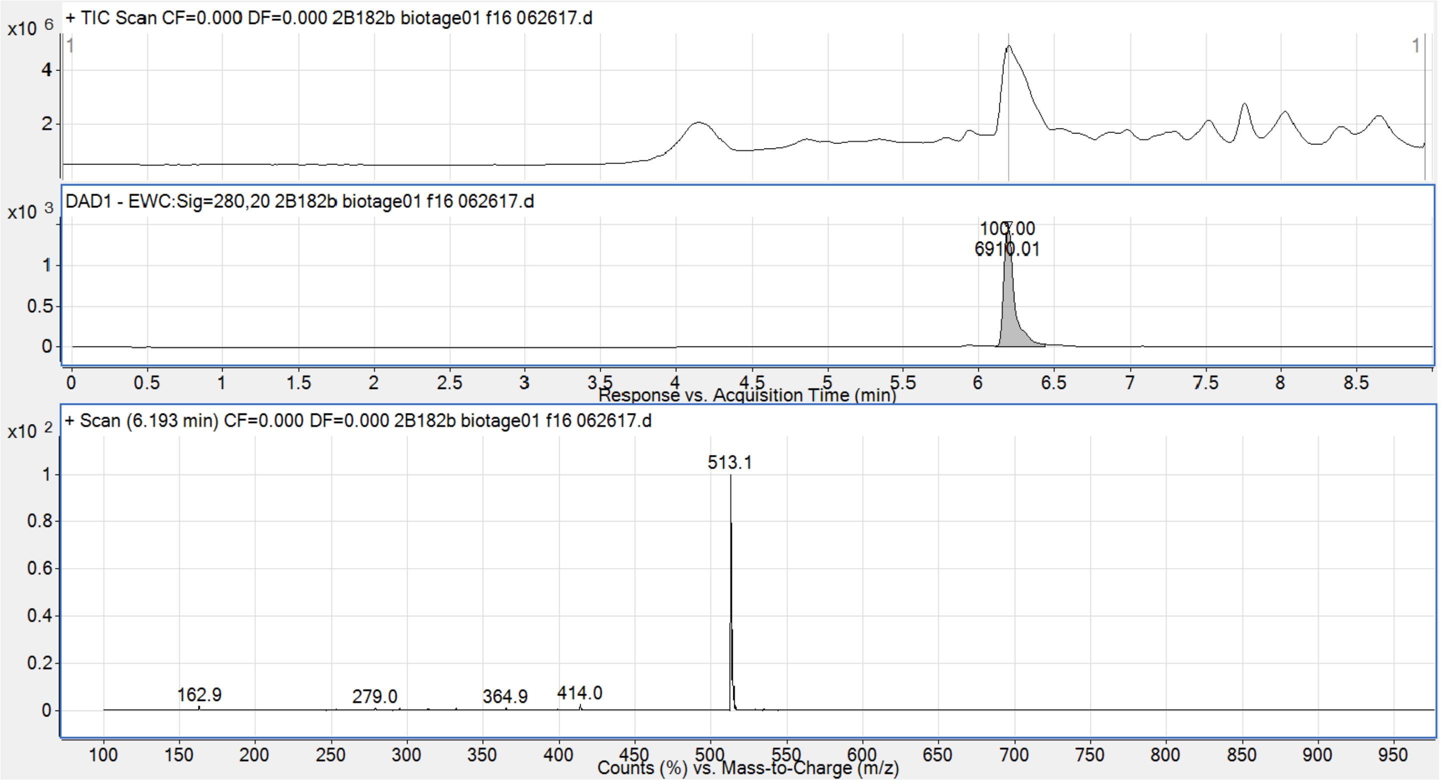

Compound **8t**

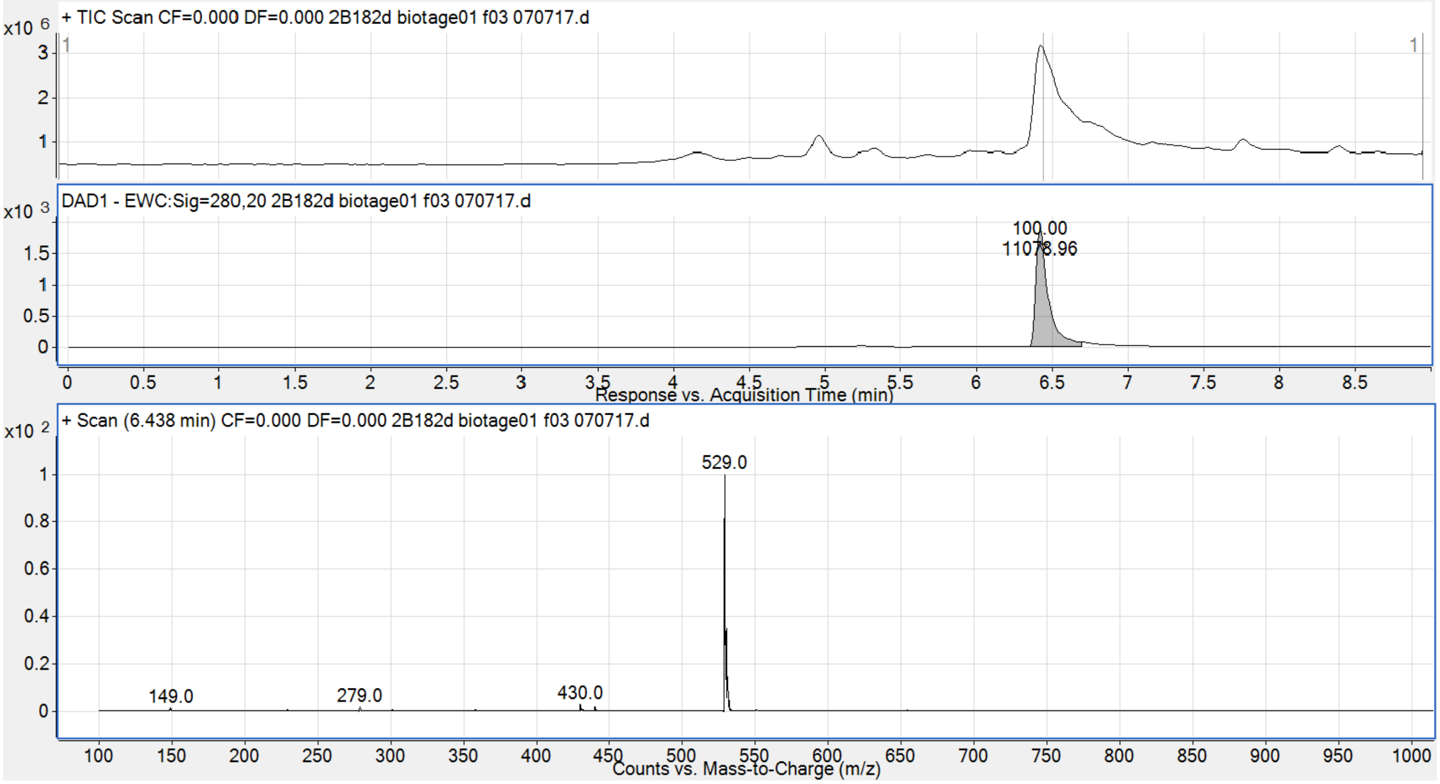

^{1}H and ^{13}C NMR

Compound **8s**

3/10/2020 9:49:17 AM

Formula	$C_{29}H_{28}N_4O_3S$	FW	512.6226

Acquisition Time (sec)	2.0486	Comment	Std proton	Date	Feb 28 2020	Date Stamp	Feb 28 2020
File Name	C:\Users\Mycoahhh\Documents\NMR\michan\2B182c-proton.fid\fid					Frequency (MHz)	499.83
Nucleus	1H	Number of Transients	16	Original Points Count	16415	Points Count	32768
Pulse Sequence	s2pul	Receiver Gain	20.00	Solvent	DMSO-d6	Spectrum Offset (Hz)	2999.0005
Spectrum Type	STANDARD	Sweep Width (Hz)	8012.82	Temperature (degree C)	30.000		

^{1}H NMR (500 MHz, DMSO-d_6) δ 8.42 (s, 1H), 8.29 (d, J = 7.83 Hz, 1H), 7.92 (dd, J = 1.47, 8.80 Hz, 1H), 7.68 - 7.82 (m, 2H), 7.54 - 7.65 (m, 3H), 7.38 - 7.50 (m, 2H), 6.95 (d, J = 3.18 Hz, 1H), 6.64 (dd, J = 1.71, 3.18 Hz, 1H), 4.11 (s, 3H), 3.86 (s, 2H), 3.45 - 3.57 (m, 1H), 1.75 (s, 2H), 1.56 - 1.66 (m, 2H), 1.45 - 1.53 (m, 1H), 1.16 - 1.29 (m, 4H), 1.02 - 1.12 (m, 1H)

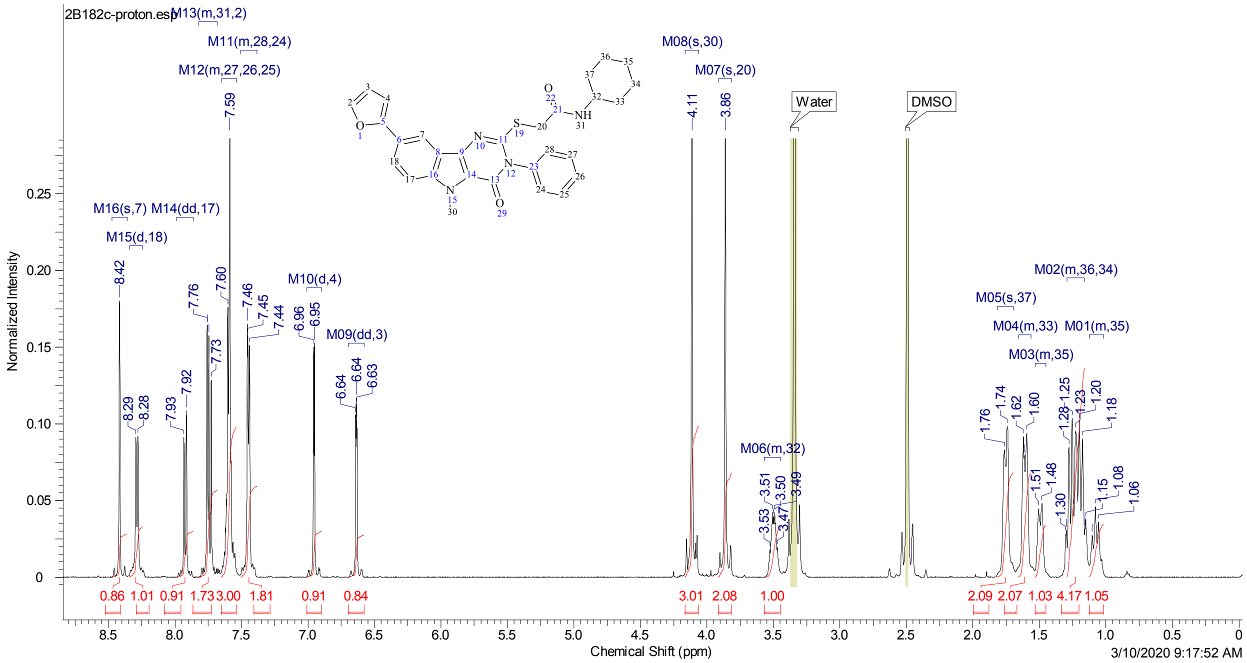

3/10/2020 9:17:52 AM

Formula	$C_{29}H_{28}N_4O_3S$	FW	512.6226

Acquisition Time (sec)	1.3005	Comment	Std carbon	Date	Feb 28 2020	Date Stamp	Feb 28 2020
File Name	C:\Users\Mycoahhh\Documents\NMR\michan\2B182c-carbon.fid\fid					Frequency (MHz)	125.69
Nucleus	13C	Number of Transients	128	Original Points Count	39649	Points Count	65536
Pulse Sequence	s2pul	Receiver Gain	30.00	Solvent	DMSO-d6	Spectrum Offset (Hz)	13144.4209
Spectrum Type	STANDARD	Sweep Width (Hz)	30487.80	Temperature (degree C)	30.000		

^{13}C NMR (126 MHz, DMSO-d_6) δ 165.8, 155.3, 153.7, 153.4, 142.4, 139.2, 137.3, 135.9, 130.0, 129.6, 129.6, 124.1, 123.3, 119.9, 119.4, 115.1, 112.2, 111.6, 104.7, 48.1, 36.8, 32.5, 31.3, 25.2, 24.6

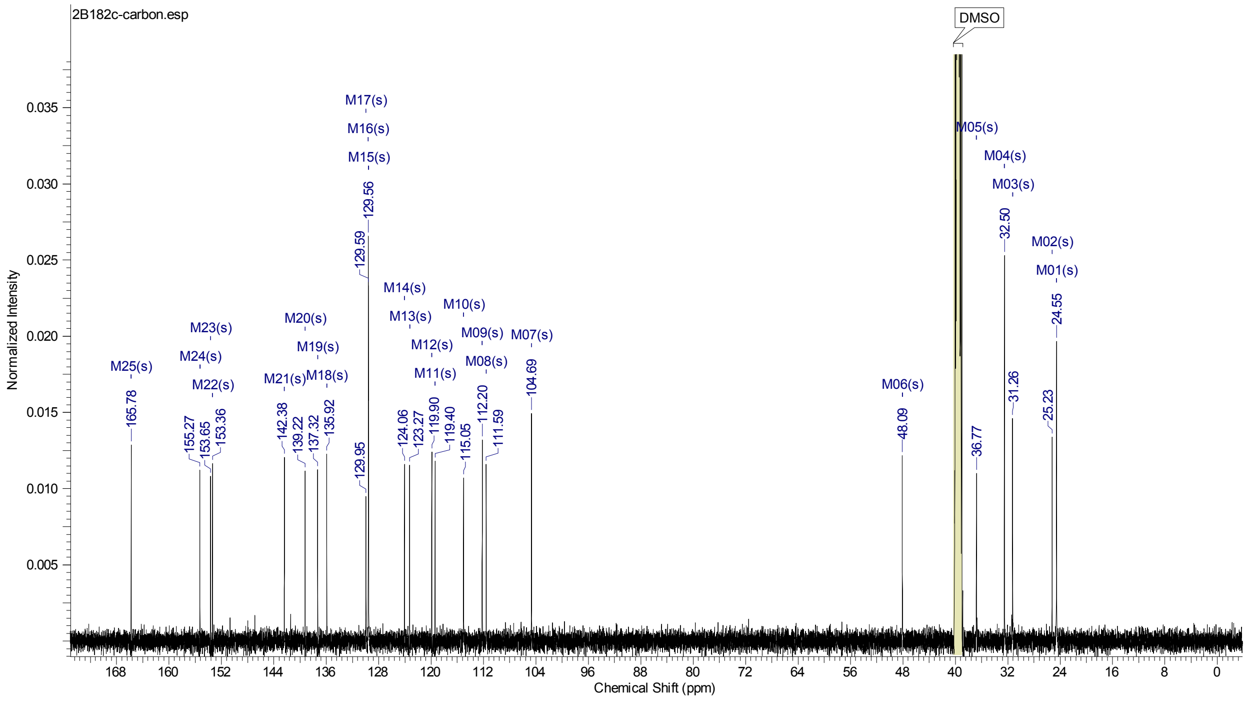

LC-MS and HRMS
Compound **8s**

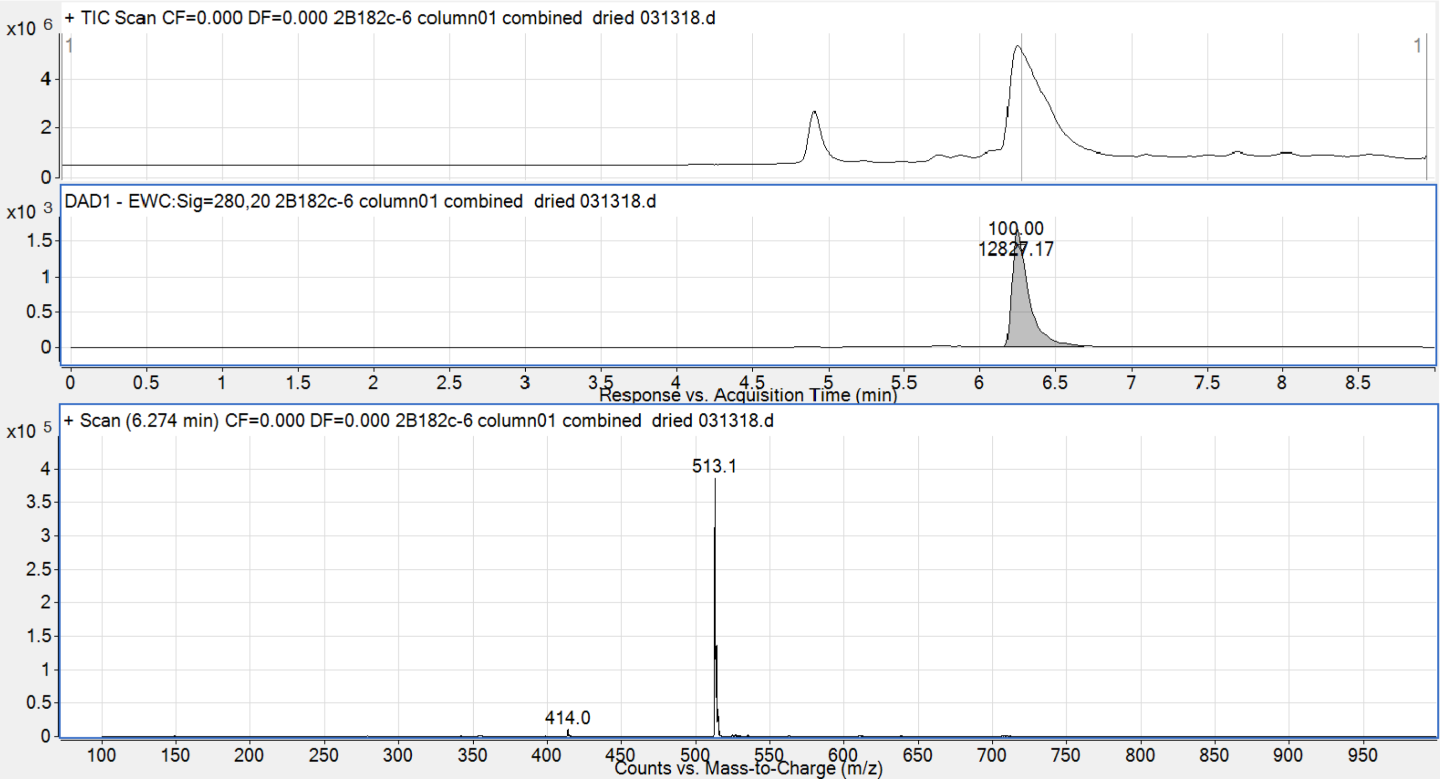

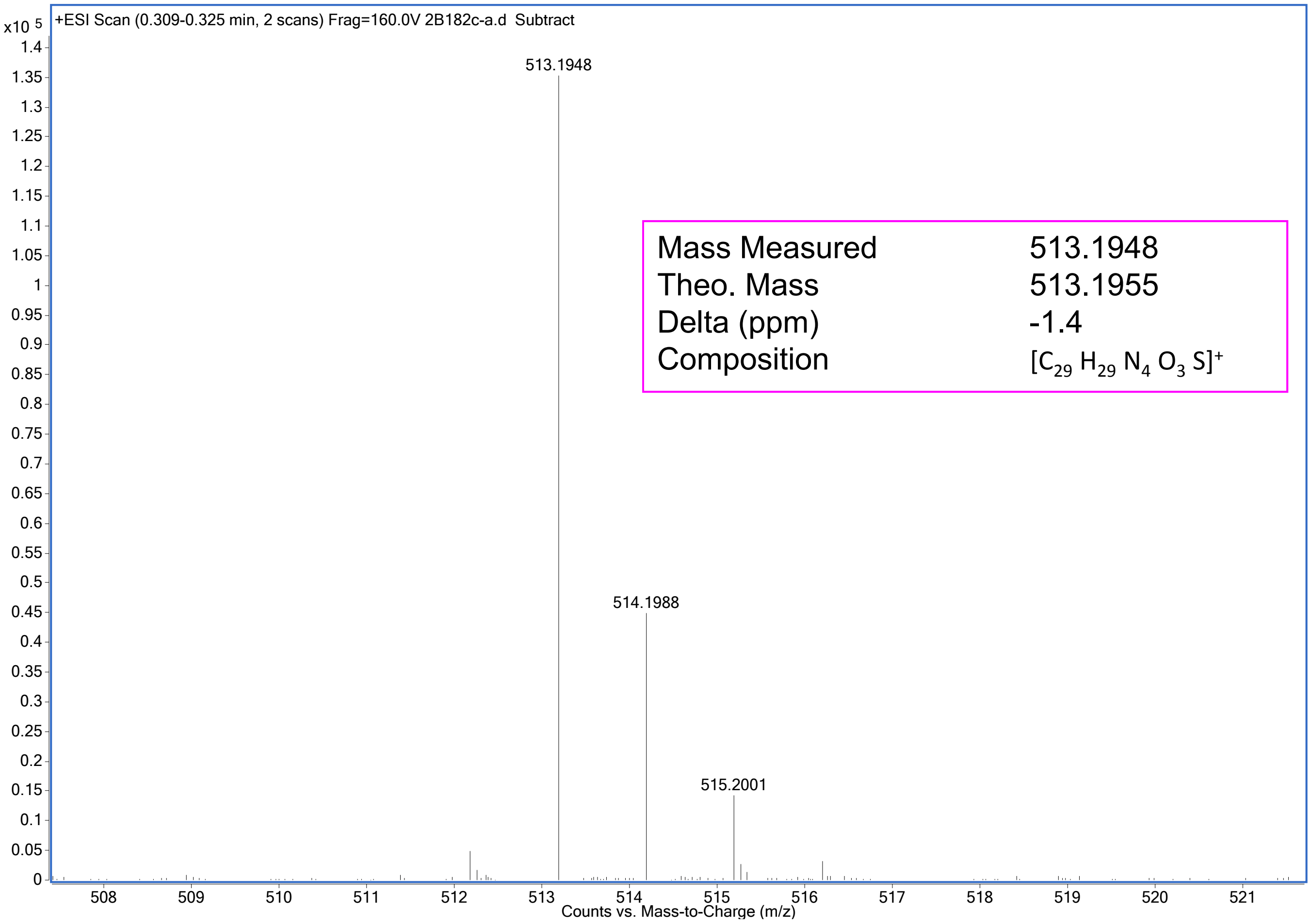

Supplement: Supplementary file 1 [file Data_Sheet_1.pdf]
